# Chinese consensus on the diagnosis and treatment of prolactinomas (2025 edition)

**DOI:** 10.1186/s41016-026-00437-7

**Published:** 2026-06-08

**Authors:** Shaojian Lin, Zhengyuan Chen, Lijin Ji, Linjie Wang, Wanyu Zhang, Xuqun Tang, Yue Wu, Qinlin Fan, Jun Li, Li Pan, Zhenwei Yao, Chengyan Deng, Hongying Ye, Sheyu Li, Huijuan Zhu, Yao Zhao, Yongfei Wang, Zhe Bao Wu, China Pituitary Adenoma Specialist Council

**Affiliations:** 1 Department of Neurosurgery, Center of Pituitary Tumor, Ruijin Hospital, Shanghai Jiao Tong University School of Medicine, Shanghai, 200025 China; 2 Department of Neurosurgery, Huashan Hospital, Shanghai Medical College, Fudan University, Shanghai, 200040 China; 3 Department of Endocrinology, Huashan Hospital, Fudan University, Shanghai, China; 4 Department of Endocrinology, Peking Union Medical College Hospital, Chinese Academy of Medical Sciences, Beijing, 100730 China; 5 Department of Gynecological Endocrinology, Peking Union Medical College Hospital, Chinese Academy of Medical Sciences, Beijing, China; 6 Department of Radiology, Huashan Hospital, Fudan University, Shanghai, China; 7 Department of Endocrinology, West China Hospital, Sichuan University, Chengdu, 610041 Sichuan China

**Keywords:** Prolactinoma, Consensus, Evidence-based medicine, Endoscopic endonasal surgery, Dopamine agonist, Pregnancy, Pituitary neuroendocrine tumor

## Abstract

Prolactinomas are the most prevalent functional pituitary adenomas. Significant advancements have been made in the diagnostic and therapeutic concepts for prolactinomas, driven by progress in surgical techniques and the accumulation of new clinical evidence-based medicine. Based on the 2014 version of the “Chinese Consensus on the Diagnosis and Treatment of Pituitary Prolactin Adenoma,” the China Pituitary Adenoma Specialist Council has revised this consensus in accordance with the latest evidence-based medical finding and clinical experience, aiming to provide standardized diagnosis and treatment recommendations for clinicians. The consensus systematically elaborates on the epidemiological characteristics, clinical manifestations, diagnostic criteria, differential diagnosis process, and treatment strategies for prolactinomas, and proposes individualized management suggestions for special populations (such as pregnant women, male patients, and refractory cases). Treatment decisions strongly advocate for comprehensive evaluation by a multidisciplinary team of experts, including neurosurgery, endocrinology, radiology, radiation oncology, pathology, ophthalmology, and obstetrics/gynecology. Through the formulation of recommendation questions, evidence summary and evaluation, formation of recommendation opinions, expert discussions, and integration with clinical practice, 36 recommendations have been developed covering aspects such as screening, assessment, diagnosis, treatment, and monitoring. This consensus balances clinical needs with international evidence-based standards, provides a highly reliable reference for diagnosis and treatment, and is expected to enhance the standardization and overall level of prolactinoma diagnosis and treatment in China.

**Practice guideline registration**

International Practice Guideline Registration for Transparency (PREPARE-2025CN1518).

**Supplementary Information:**

The online version contains supplementary material available at 10.1186/s41016-026-00437-7.

## Background

The population prevalence of pituitary prolactin (PRL) adenomas is approximately 37 per 100,000, with an annual incidence as high as 2.0–8.2 per 100,000, making them the most common functional pituitary neuroendocrine tumors (accounting for 46–66%) [1]. Women of reproductive age are the most affected group, with a female-to-male prevalence ratio of 10:1 in the 25–44 age group, while the difference is not statistically significant in individuals over 50 years of age (postmenopausal in women) [2, 3]. Clinical manifestations primarily include hypogonadotropic hypogonadism, galactorrhea, and weight gain due to hyperprolactinemia. Female patients may present with menstrual disorders, galactorrhea, and infertility, while male patients may exhibit hypogonadism, gynecomastia, and infertility. Secondly, patients may experience local mass effect symptoms such as headache, visual dysfunction, and other anterior pituitary hormone deficiencies [1, 4]. In 2014, the China Pituitary Adenoma Specialist Council (CPASC) formulated and published the “Chinese Consensus on the Diagnosis and Treatment of Pituitary Prolactin Adenoma (2014 Edition)” [5]. Over the past decade, significant changes have occurred in the diagnostic and therapeutic concepts for prolactinomas due to advancements in surgical techniques and updates in clinical evidence-based medicine. To improve and standardize clinicians’ understanding and management of this disease, the CPASC has reconvened an expert team to revise this new edition of the consensus.

## Consensus development methodology

### Consensus development process

Initiated by the CPASC in October 2024, this consensus followed the guidance from the US Institute of Medicine [6], the World Health Organization Handbook for Guideline Development [7], the Guiding Principles for the Formulation and Revision of Clinical Diagnosis and Treatment Guidelines in China (2022 Edition) [8], Formulation and Implementation of Evidence-Based Clinical Practice Guidelines, the Appraisal of Guidelines for Research and Evaluation (AGREE) II instrument [9], the Scientificity, Transparency, Applicability Rankings (STAR) checklist, and the Reporting Items for practice Guidelines in HealThcare (RIGHT) statement.

### Consensus working group

A total of 60 experts formed the working group, including the advisory committee, secretariats, evidence assessment team, clinical panel, and external reviewers. The multidisciplinary team (MDT) included clinicians from neurosurgery, endocrinology, obstetrics and gynecology, neuroradiology, radiation oncology, and evidence-based medicine methodologists. The consensus working group established a core team and clinical expert committee to provide evidence-based recommendations focused on key clinical questions related to the screening, diagnosis, and treatment of prolactinomas. The MAGIC China Center at West China Hospital of Sichuan University supported the consensus development process following the Grading of Recommendations Assessment, Development and Evaluation (GRADE) approaches for evidence certainty rating and formulation of recommendations. Conflicts of interest declaration is compulsory among all participants.

### Users and target population

The consensus informed neurosurgeons, endocrinologists, clinicians, gynecologists, and radiologists in treating patients with prolactinomas.

### Priority of guideline questions

The core team proposed candidate guideline questions through discussion in meetings and reference from previous consensuses before importance rating by clinical panels using 5-point Likert scale, with 5 being most important and 1 least important. The full panel rated the importance of outcomes using Delphi method and a 5-point Likert scale. The clinical panels prioritized guideline questions based on their importance and the capacity of the consensus expert team through a one-round Delphi survey (the 2nd round of survey was waived due to high consistency). Incorporating with methodologists, the core team translated the guideline questions with one or more evidence-based PICO (Population, Intervention, Comparison, Outcome) questions, which can be informed by synthesized evidence. The working group finalized 36 guideline questions for this consensus.

### Literature search

The working group searched the databases including China National Knowledge Infrastructure (CNKI), Chinese Scientific Journals Database (VIP), Wanfang Medical Database (Wanfang Data), China Biology Medicine disc (CBM), the US National Library of Medicine (MEDLINE), and the Excerpta Medica Database (Embase), covering publications from database inception to April 30, 2025. The Chinese search terms included “prolactinoma”, “prolactin-secreting adenoma”, “microprolactinoma”, and “macroprolactinoma”. The English search terms included “Prolactinoma,” “Prolactin-Secreting Pituitary Adenoma,” “Lactotroph Adenoma,” “Microprolactinoma,” and “Macroprolactinoma.” The working group limited the search to publications in Chinese and English and conducted on human subjects.

### Evidence grading and strength of recommendations

The evidence assessment team rated the certainty of evidence as high, moderate, low, or very low using the GRADE approaches (see Supplementary Table 1). They developed evidence summaries following the GRADE Evidence-to-Decision (EtD) framework, synthesizing benefits and harms of interventions, patient values and preferences, cost-effectiveness, resource utilization, intervention accessibility, feasibility, and healthcare equity. The consensus statements and recommendations as well as the strength of recommendations (see Supplementary Table 2) were finalized through dozens of rounds of core team meetings and two rounds of full panel meetings (virtual for the first round and face-to-face in the second round). For recommendations critical for practice, but not necessarily evidence-based, the consensus considered them as Good Practice Statements (GPS) [10]. The consensus was reached for an agreement over 80%.

### Diagnosis and differential diagnosis

#### Recommendation 1

Serum PRL measurement is recommended in all patients with any clinical suspicion: (1) child-bearing aged females presenting with menstrual abnormalities (irregularity, amenorrhea, or prolonged menstrual cycles), infertility, and/or galactorrhea; (2) males with decreased libido, erectile dysfunction, infertility, gynecomastia, and/or galactorrhea; (3) adolescents with delayed or arrested puberty, and children or adolescents with growth retardation; (4) imaging examinations suggestive of a sellar region lesion (evidence level: low; strength of recommendation: strong).

Among child-bearing aged female patients with hyperprolactinemia, 85–90% present with oligomenorrhea or amenorrhea, 69–84% with galactorrhea, and infertility is also a common clinical manifestation [11]. Among female patients with oligomenorrhea or secondary amenorrhea, 7.23–17.34% have hyperprolactinemia [12–16]. The main manifestations of gonadal dysfunction in male patients with hyperprolactinemia include sexual dysfunction, infertility, and gynecomastia (with or without galactorrhea) [17, 18]. In pediatric and adolescent patients with prolactinoma, in addition to delayed or arrested puberty, 15.0–25.9% of male cases may present with growth retardation [15, 19].

Patients with prolactinomas may seek medical attention due to symptoms associated with mass effect, such as headache and visual dysfunction [18]. For incidentally detected pituitary tumors identified on imaging examinations conducted for unrelated indications, evaluation of pituitary function—including serum PRL measurement—is recommended [20].

Under physiological conditions, PRL is secreted in a pulsatile manner; its serum levels rise during sleep and early morning hours, and reach nadir between 10:00 and 12:00 a.m. [21, 22]. In addition, PRL secretion can be stimulated by stressful events such as emotional agitation and strenuous physical activity, as well as the ingestion of a high-protein diet [23]. For the most reliable assessment of serum PRL levels, blood sampling should be performed in a resting state following an overnight fast and a minimum of 2 h of wakefulness [21, 24]. In patients with mildly elevated serum PRL levels (< 100 ng/ml or 2120 mU/L), a repeat PRL assay is recommended [1].

Although the overall quality of evidence supporting this recommendation is relatively low, current evidence indicates that serum PRL measurement for the diagnosis of pituitary prolactinoma can yield clinical benefits at a relatively low cost; hence, a strong recommendation is made [25].

#### Recommendation 2

In patients with hyperprolactinemia whose clinical manifestations are discordant with serum PRL levels or who lack typical clinical features, screening for macroprolactin is recommended (evidence level: low; strength of recommendation: weak).

Three forms of PRL exist in human serum: monomer (23,000 Da), dimer (40,000–60,000 Da), and macroprolactin (> 100,000 Da). The PRL monomer is the major component and possesses biological activity. Macroprolactin is a complex formed by immunoglobulin G (IgG) and PRL monomers, characterized by low renal clearance, a prolonged half-life, and lack of biological activity, yet it can be detected by standard assays [2]. The reported detection rate of macroprolactinemia among patients with hyperprolactinemia ranges from 5.32 to 22.90% [26–29]. Therefore, screening for macroprolactin is recommended in patients with discordant clinical and biochemical findings—i.e., marked elevation of measured PRL without corresponding clinical manifestations such as menstrual disturbance, infertility, or galactorrhea.

The polyethylene glycol (PEG) precipitation method is used clinically for screening. A PRL monomer recovery rate < 40% suggests macroprolactinemia [30].

#### Recommendation 3

In patients with pituitary macroadenoma who exhibit clinical features consistent with hyperprolactinemia despite normal or mildly elevated PRL levels, serum dilution and repeat PRL measurement are recommended to avoid missed diagnosis of prolactinoma by excluding the Hook effect (evidence level: low; strength of recommendation: weak). For patients with serum PRL levels exceeding the upper limit of the assay range (reported as > 200 ng/ml or 4240 mU/L), dilution testing is recommended to obtain the true absolute value for accurate monitoring of treatment response (evidence level: low; strength of recommendation: weak).

The Hook effect occurs when extremely high serum PRL concentrations saturate the capture antibody in two-site immunoradiometric assays, thereby reducing the availability of binding sites for the detection antibody, which results in falsely low PRL despite markedly elevated actual levels. The Hook effect is uncommon with modern chemiluminescent immunoassay [31]. A 2025 systematic review included 61 patients with macroprolactinoma and documented Hook effect. The smallest tumor in this cohort had a volume of 3.4 cm^3^ and a maximum diameter of 2.9 cm. The mean PRL concentration was 108.1 ng/ml before dilution and 38,526.9 ng/ml after dilution [32].

Therefore, in patients with pituitary macroadenoma who present with clinical manifestations of hyperprolactinemia but have normal or mildly elevated PRL levels (< 100 ng/ml or 2120 mU/L)—particularly those with a maximum tumor diameter ≥ 3 cm—the Hook effect should be suspected. A 1:100 dilution assay is recommended to confirm or exclude this phenomenon [24].

#### Recommendation 4

For patients with hyperprolactinemia, comprehensive medical history taking and physical examination are indicated. Prior to establishing a diagnosis of pituitary prolactinoma, it is imperative to exclude physiological, pharmacological, and other pathological etiologies unrelated to pituitary prolactinoma (Good Practice Statement).

Hyperprolactinemia may arise from a wide array of physiological, pharmacological, and pathological causes (see Supplementary Table 3) [1]. A detailed medical history should be elicited and a full physical examination conducted for all the patients, with priority given to ruling out pregnancy and obtaining a complete account of their medication history.

Prolactinoma represents the most prevalent pathological cause; however, it is critical to differentiate it from the following entities: systemic disorders (e.g., primary hypothyroidism, liver cirrhosis, and renal insufficiency); hypothalamic-pituitary diseases (e.g., pituitary stalk effect secondary to other types of pituitary macroadenomas, craniopharyngioma, germ cell tumors, histiocytosis, and intracranial hypotension); neurogenic causes (e.g., chest wall injury, spinal cord injury, and breast stimulation); inflammatory or destructive lesions (e.g., meningitis, tuberculosis, and cranial radiotherapy); ectopic PRL-secreting neoplasms (e.g., renal cell carcinoma, gonadoblastoma, ovarian teratoma, and perivascular epithelioid cell tumors); PRL receptor gene mutation.

#### Recommendation 5

It is recommended that the following high-risk groups undergo genetic mutation testing, with priority given to screening for mutations in the multiple endocrine neoplasia type 1 gene (*MEN1*) and the aryl hydrocarbon receptor-interacting protein gene (*AIP*): (1) young patients (age of onset < 20 years) with macroadenomas (especially giant adenomas with a diameter ≥ 4 cm); (2) those with a family history of familial isolated pituitary adenoma (FIPA) or multiple endocrine neoplasia (MEN) (evidence level: low; strength of recommendation: weak recommendation).

In the diagnosis and management of pediatric and adolescent patients with pituitary prolactinomas, special attention should be given to family history, recording the age of onset, tumor size, and invasiveness. The overall genetic mutation rate in pituitary prolactinoma patients is approximately 2.8%, while in children or adolescents (age < 20 years), the mutation rate is nearly 10% [33, 34]. Specifically, the MEN1 mutation rate in patients with giant adenomas is as high as 21.9% (7/32), and the AIP mutation rate is 18.8% (6/32) [35]. Currently, there is a lack of research data from the Chinese population. Different genetic mutation types exhibit significant clinical differences: patients with MEN1 mutations often present with dopamine agonist (DA) resistance and may concurrently develop other endocrine disorders such as hyperparathyroidism and pancreatic neuroendocrine tumors, necessitating early identification and long-term follow-up. Patients with AIP mutations tend to have larger pituitary tumor volumes and stronger invasiveness, requiring the development of a MDT treatment plan. For patients identified with genetic mutations, genetic counseling and screening for family members are recommended. Existing evidence indicates that patients with an age of onset ≥ 30 years and those with microadenomas have a low genetic mutation rate, making routine genetic testing of limited value for these populations [36, 37]. Thus, *MEN1/AIP* gene mutation testing is indicated for high-risk subgroups (age < 20 years with macroadenomas, familial history, or sporadic males < 18 years) but is not recommended for patients aged ≥ 30 years or those with microadenomas to avoid waste of medical resources.

### Imaging

#### Recommendation 6

Patients diagnosed with hyperprolactinemia after exclusion of physiological, pharmacological, and other systemic etiologies should undergo pituitary magnetic resonance imaging (MRI) (evidence level: low; strength of recommendation: weak). Pituitary dynamic contrast-enhanced MRI is recommended to improve the detection rate and localization accuracy of microprolactinomas (evidence level: very low; strength of recommendation: weak).

Pituitary MRI is the preferred imaging modality for diagnosis and monitoring prolactinomas, offering high soft tissue resolution and multi-planar imaging [38–40]. Non-contrast MRI has limited sensitivity in detecting microadenomas; therefore, contrast-enhanced pituitary MRI remains the most primary imaging method for detecting prolactinomas, especially microadenomas smaller than 1 cm. Computed tomography (CT) is primarily advantageous for visualizing bone structures and calcifications but provides inferior soft tissue contrast and is less effective in delineating adenomas. It is currently reserved mainly for patients with contraindications to MRI and for preoperative assessment of sphenoid sinus anatomy.

Routine pituitary MRI protocols include pre-contrast, sagittal, and coronal T1-weighted imaging (T1WI), T2-weighted imaging (T2WI), and post-contrast T1WI sequences [39]. Most prolactinomas present as microadenomas on MRI, appearing isointense or slightly hypointense on T1WI, and with hypoenhancement after contrast administration. On T2WI, lesions typically appear mildly hyperintense; however, male prolactinoma patients are more likely to demonstrate heterogeneous T2WI signal patterns (e.g., due to hemorrhage or necrosis); some studies suggest that T2WI heterogeneity may be associated with a suboptimal response to DA [38, 40, 41]. With advances in imaging technology, the use of 3D acquisition techniques (e.g., 3D fast spin-echo/gradient echo sequences) enables the acquisition of MRI images with slice thicknesses < 1 mm, thereby improving the detection of smaller microadenomas [42–44].

Domestic and international studies have confirmed that pituitary dynamic contrast-enhanced scanning, by obtaining rapid, sequential images during contrast passage, can improve the detection rate of microadenomas [40, 45]. The pituitary gland has a dual blood supply and enhances markedly in the early phase, while adenomas enhance more slowly and to a lesser degree than normal pituitary tissue. Dynamic contrast-enhanced MRI can better capture hemodynamic changes in both the pituitary and microadenomas, obtaining optimal contrast between them and enabling more precise delineation of the lesion.

#### Recommendation 7

The frequency of imaging follow-up for treated prolactinomas should be determined based on a comprehensive assessment of clinical symptoms, biochemical results, previous imaging features, and histopathological results (Good Practice Statement). If new symptoms such as visual dysfunction, headache, galactorrhea, or pituitary deficiency occur, an increased frequency of follow-up is recommended (Good Practice Statement).

Due to the risk of gadolinium retention in the body [46, 47], it is recommended to fully utilize clinical symptom follow-up and serum PRL monitoring to reasonably set the frequency of imaging follow-up to safely assess treatment efficacy, and prioritize the use of macrocyclic chelating agents over linear agents. Macroadenomas can undergo MRI re-evaluation 3–6 months after initiation of DA therapy. The follow-up interval for microadenomas should be individualized based on clinical manifestations and biochemical follow-up results [48]. In patients demonstrating a favorable response to DA, MRI may be repeated annually or at longer intervals. If drug resistance is suspected or new symptoms arise, such as visual disturbances, headache, galactorrhea, or pituitary deficiency, prompt MRI is necessary [49]. Postoperative MRI is recommended at 3 months for surgical patients. For tumors that are DA-resistant, partially resected, or exhibit high invasive potential, more frequent imaging surveillance is advised [50].

#### Recommendation 8

For prolactinoma patients with the following risk factors, preoperative CT angiography (CTA) or MR angiography (MRA) is suggested to screen for associated aneurysms: (1) invasive tumors; (2) tumor directly contacting the internal carotid artery; (3) middle-aged and elderly patients (> 50 years); (4) suspicious imaging signs of an aneurysm on head/pituitary MRI; (5) previous history of surgery for sellar region lesions, diagnosis of refractory pituitary tumor, and/or history of cerebrovascular disease (evidence level: very low; strength of recommendation: weak).

Based on domestic and international research, factors such as invasive pituitary tumors, direct contact between the tumor and adjacent arteries, and advanced patient age are associated with an increased incidence of coexisting intracranial aneurysms in patients with pituitary tumors [51, 52]. The incidence of aneurysms associated with pituitary tumors ranges from 2.3 to 8.3%, higher than that in the general population (approximately 3%) [53]. Among functional pituitary adenomas with associated aneurysms, prolactinomas and growth hormone-secreting adenomas are the most commonly observed subtypes, with prolactinomas accounting for 18.8–36.5% [53, 54]. The cavernous segment of the internal carotid artery is the most frequent location for associated aneurysms in these patients. When an aneurysm is closely attached with the tumor tissue, endoscopic endonasal resection of the pituitary tumor may pose a risk of inducing fatal aneurysm rupture [53, 55, 56]. Therefore, for prolactinoma patients with the aforementioned risk factors or suspicious signs of aneurysm on routine MRI (flow voids, pulsation artifacts), preoperative CTA or MRA is recommended to detect potential aneurysms.

### Treatment

The treatment goals for prolactinomas are to lower PRL levels, aiming to restore normal gonadal axis function, shrink or eliminate the tumor, and prevent recurrence. Treatment options include medication, surgery, and radiotherapy. DA are the first-line medical treatment for prolactinomas, including bromocriptine and cabergoline, which effectively reduce serum PRL levels, shrink adenoma size, and restore gonadal axis function. Surgery includes transsphenoidal and transcranial approaches. Currently, endoscopic endonasal transsphenoidal surgery has become the mainstream surgical approach for the vast majority of pituitary tumor patients. Radiotherapy includes stereotactic radiosurgery (SRS) and external beam radiotherapy (EBRT). Common SRS devices include Gamma Knife, linear accelerator-based systems like CyberKnife, and proton beam therapy. Current research on SRS for prolactinomas primarily comes from Gamma Knife studies, with limited reports on CyberKnife or proton therapy.

**Fig. 1:**
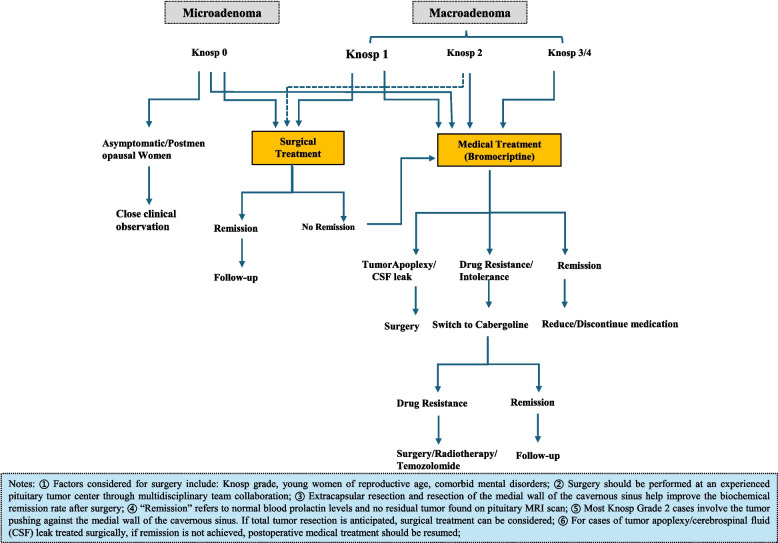
The treatment flowchart of prolactinoma

In clinical practice, it is recommended to establish a “patient-centered” MDT model involving experts from neurosurgery, endocrinology, radiology, radiation oncology, pathology, ophthalmology, and obstetrics/gynecology. This facilitates individualized and precise treatment for patients with complex and difficult cases. The treatment flowchart is shown in Fig. 1. Recommendations for medication, surgery, and radiotherapy are detailed below.

### Pharmacological treatment

#### Recommendation 9

DA therapy is effective in lowering serum PRL levels, improving clinical manifestations associated with hyperprolactinemia, and reducing adenoma size (evidence level: moderate; strength of recommendation: strong).

Retrospective studies show that bromocriptine can normalize PRL levels in 68% of prolactinoma patients and tumor reduction in 62% of patients [57, 58]. A review of literature from 2000 to 2018, including 1362 prolactinoma patients, found that the overall biochemical remission rate for patients taking cabergoline was 79.7%, with 84.0% for 541 microadenoma and 76.9% for 821 macroadenoma; 73.9% of patients reported tumor volume reduction [59–63]. The recommended dose for bromocriptine is 2.5–15 mg daily, divided into 2–3 doses (half-life 4–6 h). The recommended dose for cabergoline is 0.5–3.5 mg per week (half-life 80 h). As cabergoline is not yet available in mainland China, bromocriptine remains the first-choice DA.

Before initiating DA therapy, patients should be informed about potential common adverse effects, including gastrointestinal discomfort (nausea, vomiting, constipation, abdominal distension), headache, dizziness, fatigue, nasal congestion, and orthostatic hypotension. These often occur at the start of treatment, vary in severity among individuals, are tolerable for most patients, and rarely affect medication adherence. The overall incidence of adverse effects with bromocriptine is about 1.43 times than that of cabergoline [64]. Results from randomized controlled studies show that compared to cabergoline, bromocriptine treatment leads to more frequent gastrointestinal reactions: nausea incidence ~ 50% (vs. 31% for cabergoline), vomiting 10% (vs. 4% for cabergoline); other adverse effects are similar: headache 29% vs. 30%, dizziness 26% vs. 25%, gastrointestinal discomfort 20% vs. 15%; the proportion of patients discontinuing medication due to adverse effects was higher for bromocriptine than cabergoline (12% vs. 3%) [65]. It is recommended to initiate treatment with a low dose and gradually titrate upward to minimize gastrointestinal irritation and orthostatic hypotension. Taking the medication with meals or at bedtime can improve tolerance. Bromocriptine can be started at 0.625–1.25 mg/day, gradually increased to the effective dose; cabergoline is typically initiated at 0.25–0.5 mg/week, gradually increased. If drug intolerance occurs, switching to another DA or selecting an alternative treatment plan can be attempted [66, 67].

DA therapy may cause impulse control disorders (ICDs), such as pathological gambling, compulsive shopping, and hypersexuality. The reported incidence in the prolactinoma population varies widely (9.8–52%), with domestic reports ranging from 9.8 to 24.4%, possibly due to differences in diagnostic criteria and ethnicity. Patients requiring long-term DA therapy, especially males, young patients (< 40 years), those with a history of smoking/alcohol use, or psychiatric history like anxiety/depression, should be fully informed about the significantly increased risk and monitored for early identification. If ICD symptoms appear, consultation with psychiatry is recommended, prioritizing reduction of the DA dose or switching to another DA type; if symptoms are severe or persistent, other treatment options should be considered [68].

#### Recommendation 10

For patients with Knosp grade 0 or 1, well-defined microadenomas or macroadenomas, before initiating treatment, the MDT should fully inform the patient of the pros and cons of surgical versus pharmacological treatment options. Combined with patient preference, either surgery or DA can be chosen as the first-line treatment (evidence level: low; strength of recommendation: weak).

#### Recommendation 11

For patients with Knosp grade ≥ 2 prolactinomas, especially invasive macroprolactinomas or giant adenomas, DA therapy is the first choice (evidence level: low; strength of recommendation: weak).

For prolactinomas Knosp grade 2 or higher (see Supplementary Table 4), especially invasive and giant adenomas, choosing DA therapy first can lead to tumor volume reduction, relief of neurological compression symptoms, and decreased PRL levels in most cases [69]. A meta-analysis summarizing clinical characteristics of 196 giant prolactinoma patients (male:female ratio 3.6:1, median age 38 years, tumor diameter 53 mm) found that 82% received primary pharmacological treatment. After drug therapy, 88% had tumor shrinkage, 85% had visual improvement, and 51% achieved normal PRL levels. Patients who underwent surgery all had residual tumor and persistent hyperprolactinemia, and postoperative hypothalamic–pituitary–adrenal (HPA) axis and hypothalamic-pituitary-thyroid (HPT) axis deficiencies were more common than in the pharmacologically treated group, along with a certain rate of treatment complications [34].

Currently, the impact of DA therapy on inducing tumor fibrosis and whether preoperative DA treatment adversely affects surgery remains controversial. Most scholars believe that previous bromocriptine treatment may induce tumor fibrosis, leading to tumor hardening, decreased biochemical remission rates, and increased perioperative complications, while evidence for cabergoline is insufficient [70–73]. A domestic single-center cohort study of 290 patients showed a significantly higher rate of hard tumor consistency in the previous bromocriptine treatment group compared to the direct surgery group (27.8% vs. 9.8%). Cumulative bromocriptine dose > 206 mg and treatment duration > 2.5 months were associated with tumor hardening. Furthermore, compared to the direct surgery group, the previous bromocriptine treatment group had more perioperative complications and lower postoperative biochemical remission rates [74]. Therefore, attention should be paid to the potential for preoperative medication to increase tumor fibrosis, thereby reducing the gross total resection rate. It is recommended to assess efficacy shortly after starting medication (e.g., 3 months) and adjust the treatment plan if necessary. Conversely, some scholars believe that short-term preoperative bromocriptine treatment helps improve gross total resection rates without increasing perioperative complications [75, 76].

#### Recommendation 12

For microprolactinoma patients with no hyperprolactinemia related clinical manifestations, observation is suggested. Follow-up should include clinical symptoms, PRL levels, and pituitary MRI. Treatment should be initiated if symptoms of hyperprolactinemia appear or if the tumor enlarges (evidence level: very low; strength of recommendation: weak).

The natural history of untreated microprolactinoma patients showed rare significant or persistent tumor growth [77]. As estrogen is a stimulator of PRL, serum PRL levels physiologically decline after menopause. Among untreated women with microprolactinomas, 45% achieved normal serum PRL levels after menopause [78]. Postmenopausal women with microprolactinomas who received at least 2 years of DA therapy and maintained normal PRL levels had a 50–73% chance of maintaining normal serum PRL levels after DA withdrawal [79, 80]. For postmenopausal women found to have microprolactinomas, observation with periodic PRL level monitoring can be chosen [81–83]. Current evidence does not support treating asymptomatic microprolactinomas in postmenopausal women.

#### Recommendation 13

For patients resistant to bromocriptine, switching to cabergoline is recommended if available (evidence level: very low; strength of recommendation: weak). For patients resistant to cabergoline, discussion by a MDT is recommended to choose among surgical treatment, increasing the drug dose to the maximum tolerated level, radiotherapy, or comprehensive treatment including temozolomide (TMZ) (evidence level: very low; strength of recommendation: weak).

DA resistance is defined as failure to normalize PRL levels and failure to achieve ≥ 50% tumor volume reduction after at least 3–6 months of treatment with the recommended doses (bromocriptine 15 mg/day, cabergoline 2 mg/week). The incidence of bromocriptine resistance is 20–30%, while that for cabergoline is 10% [63, 84]. For bromocriptine-resistant patients, switching to cabergoline is recommended, starting at a dose of 2 mg per week. If PRL normalization is not achieved, the dose can be increased as appropriate (patient informed consent is required for off-label high-dose use). For cabergoline-resistant patients, MDT discussion is recommended to choose among surgery, increased drug dose, radiotherapy, or other comprehensive treatments.

#### Recommendation 14

Patients receiving long-term, high-dose cabergoline therapy are recommended to undergo periodic echocardiography to assess cardiac valve status (evidence level: low; strength of recommendation: weak).

Meta-analysis results indicate that the incidence of cabergoline-associated valvular heart disease in prolactinoma patients is only 0.11% [85]. A baseline echocardiogram before starting treatment is recommended to assess for pre-existing valve abnormalities. Routine echocardiography is recommended for prolactinoma patients requiring long-term use of high-dose cabergoline (e.g., > 2 mg/week), treatment duration > 5 years, age > 50 years, or those with cardiac auscultatory murmurs. Follow-up echocardiography every 2–3 years is suggested thereafter, but clinical context and exclusion of other causes of valvular disease (age, hypertension, other cardiovascular diseases, etc.) are necessary [86]. If cabergoline-associated valvulopathy is suspected, multidisciplinary consultation is recommended for definitive diagnosis, and the risks and benefits of continuing the medication should be carefully weighed; switching to bromocriptine or other treatment options (e.g., surgery) may be considered. Current research does not support routine echocardiographic monitoring for patients receiving bromocriptine treatment.

#### Recommendation 15

For microadenoma patients receiving DA therapy, after PRL levels normalize, continuing the current dose for 3–6 months is suggested (evidence level: low; strength of recommendation: weak). Subsequently, when PRL levels remain within the reference range, gradual dose reduction can be considered, monitoring PRL levels, and titrating the dose down to the minimum necessary to maintain normal PRL (evidence level: very low; strength of recommendation: weak). For macroadenoma patients, drug dose reduction should be assessed in conjunction with tumor shrinkage (evidence level: low; strength of recommendation: weak).

#### Recommendation 16

For prolactinoma patients on low-maintenance dose DA therapy for more than 2 years, with normal PRL levels and a notable reduction in tumor size or absence of a visible mass on MRI, drug withdrawal can be considered (evidence level: very low; strength of recommendation: weak).

#### Recommendation 17

Female prolactinoma patients who reach menopause and meet the above withdrawal criteria are encouraged to attempt drug withdrawal with close follow-up. If pituitary lesion enlargement is found during follow-up, DA therapy can be restarted after evaluation (evidence level: low; strength of recommendation: weak).

After symptom relief and normalization of PRL levels with medical treatment, the DA dose can be gradually reduced or the dosing interval extended. If the lowest effective dose is maintained stably for at least 2 years, with serum PRL levels remaining normal and imaging showing significant tumor shrinkage or no obvious residual tumor, drug withdrawal can be considered [87–89]. A meta-analysis of 19 studies involving 743 patients showed that the withdrawal remission rate for microadenoma patients (21%) was slightly higher than for macroadenomas (16%), patients treated for more than 2 years had a withdrawal remission rate of 34%, significantly higher than the 16% for those treated for a shorter duration, and the withdrawal remission rate for cabergoline (35%) was also higher than for bromocriptine (20%) [90, 91].

Compared to premenopausal women, postmenopausal women have a significantly lower risk of adenoma recurrence after DA withdrawal [79]. In postmenopausal women meeting strict withdrawal criteria, the long-term remission rate after withdrawal can reach 73%, with a PRL elevation recurrence rate in only 27%, most of them remain asymptomatic. An increase in serum PRL levels within 3–6 months after withdrawal can predict recurrence, while factors such as initial tumor volume, pre-withdrawal PRL levels, residual tumor size, and duration of medication are not associated with recurrence [80]. For cases of recurrence, DA therapy can be restarted after assessing clinical symptoms or tumor growth.

#### Recommendation 18

After drug withdrawal according to criteria, continued follow-up for related symptoms (menstrual cycle, galactorrhea, sexual function, etc.) is recommended. PRL levels should be checked every 3 months in the first year after withdrawal, then annually, or whenever symptoms appear. Pituitary MRI should be repeated if clinical symptoms appear alongside persistently elevated PRL levels. Patients who relapse after withdrawal can restart DA therapy. For those without clinical symptoms and no tumor growth on imaging, observation and follow-up can be chosen (evidence level: very low; strength of recommendation: weak).

Based on previous guideline recommendations, follow-up generally involves assessing clinical manifestations and monitoring PRL levels every 3 months in the first year after withdrawal, then annually thereafter. If PRL remains elevated, some studies recommend repeating MRI when PRL > 100 ng/ml or 2120 mU/L [24]. There is a lack of clinical studies specifically on patients with elevated PRL levels after withdrawal. A small study of 50 patients suggested that in those relapsed after withdrawal who exhibit no typical clinical manifestations and show no progressive enlargement of residual tumors, no tumor growth or progressive PRL elevation was observed after 30 months of follow-up without medical treatment [92]. Therefore, for patients with reproductive-age need, close monitoring of menstrual cycles and sexual function are needed after drug withdrawal. If PRL rises again, comprehensive assessment combining clinical presentation and tumor enlargement is needed before restarting DA therapy [93].

### Surgical treatment

#### Recommendation 19

Decisions regarding surgical management should be individualized following thorough clinical assessment and multidisciplinary discussion, with explicit respect for patient preference (Good Practice Statement).

#### Recommendation 20

Surgical intervention should be performed by an experienced pituitary surgical team with established expertise in pituitary surgery (Good Practice Statement).

When surgical treatment is considered, the following factors should be comprehensively evaluated: tumor size and morphology, serum PRL levels, responsiveness to medical therapy, overall health status, patient preference, and future fertility considerations [94]. Accordingly, surgical decision-making may require formulation by a MDT of experts, with full respect for patient preference and informed choice [1]. Surgery should be performed by a highly experienced pituitary surgical team to maximize surgical efficacy and minimize the risk of surgical complications. In the past decades, the advancement of endoscopic techniques, neuro-navigation and intraoperative Doppler ultrasonography, has been associated with a gradual reduction in perioperative complications. In experienced pituitary surgery centers, reported complications predominantly include cerebrospinal fluid rhinorrhea (0.7–2.0%), permanent diabetes insipidus (1.0–2.7%), and meningitis (1.4–3.0%), whereas serious events like visual impairment or internal carotid artery injury are uncommon (0–1.4%), and mortality rates are extremely low [74, 95–97].

Currently, endoscopic endonasal transsphenoidal surgery represents the predominant surgical approach, whereas transcranial or combined approaches are generally limited to selected giant, invasive cases that are refractory to medical therapy. Extracapsular resection technique has been increasingly adopted, given its potential association with improved biochemical remission and lower recurrence rates [98, 99]. Previous studies reported a pseudocapsule detection rate of 70.9% in prolactinomas, with tumor cell invasion within the pseudocapsule observed in 51.2% of cases [100]. Extracapsular tumor resection technique can significantly improve postoperative biochemical remission rates; however, it may be associated with an increased risk of hypopituitarism, thereby requiring substantial surgical expertise [101]. For tumors invading the medial wall of the cavernous sinus, selective medial wall resection may contribute to improved biochemical remission and lower recurrence rates. However, this technique is technically demanding and requires careful consideration of the risks of internal carotid artery injury, cranial nerve palsy, and significant blood loss [102].

#### Recommendation 21

For well-circumscribed microadenomas or macroadenomas (Knosp grade 0–1), surgery could be discussed as a first-line treatment option (evidence level: moderate; strength of recommendation: weak).

With advances in endoscopic techniques and the increasing adoption of extracapsular resection techniques, surgical treatment has demonstrated favorable outcomes in well-circumscribed microadenomas and macroadenomas (Knosp grade 0–1) [103].

In experienced pituitary surgery centers, immediate postoperative biochemical remission has been reported in 90–100% of patients with well-circumscribed microadenomas and in 66.7–81.0% of those with well-circumscribed macroadenomas, with an overall perioperative complication rate of less than 2% [74, 96, 104, 105]. A meta-analysis of 3564 medically treated patients and 1836 surgically treated patients showed that, in microadenomas, surgical management achieved long-term remission in approximately 83% of cases, compared with a long-term remission rate of only 36% following withdrawal of medical therapy [106]. In addition, curative surgery, compared with long-term medical therapy, may reduce the overall economic burden and improve quality of life [67, 107]. Therefore, surgical intervention can be considered a first-line treatment option for well-circumscribed microadenomas and macroadenomas (Knosp grade 0–1).

Surgical outcomes are influenced by multiple factors, including surgeon experience, tumor size, degree of invasiveness, Knosp grade, presence of a pseudocapsule, and preoperative PRL levels [108]. The Knosp grade has substantial predictive value for surgical efficacy; reported immediate postoperative remission rates range from 66.7 to 83.8% for Knosp grade 0–2 tumors, 33.3–58.8% for Knosp grade 3 tumors, and 0–41.7% for Knosp grade 4 tumors [95, 105]. Preoperative PRL levels are inversely correlated with remission rates and can serve as a reliable indicator for predicting biochemical remission [109]. Previous studies reported that when preoperative PRL levels are ≤ 200 ng/ml (4240 mU/L), postoperative remission rates can reach up to 92%, whereas remission rates decrease to approximately 40% when preoperative PRL levels exceed 200 ng/ml [106]. Among patients who achieve immediate postoperative normalization of serum PRL, long-term follow-up indicates that recurrent hyperprolactinemia occurs in approximately 3.5–18.7% of cases [96, 110]. Early postoperative PRL levels at the lower end of the normal range are generally associated with a low risk of recurrence. Notably, studies have reported that patients whose serum PRL levels decrease to ≤ 10 ng/ml (212 mU/L) on the first postoperative day experience no recurrence during 5-year follow-up, with long-term remission rates reaching 83.0–89.5% [97, 111].

#### Recommendation 22

For patients intolerant of or resistant to DA therapy, surgical treatment is recommended (evidence level: very low; strength of recommendation: weak).

#### Recommendation 23

For patients with prolactinoma apoplexy leading to rapid visual deterioration, surgical treatment is preferentially recommended (evidence level: very low; strength of recommendation: weak).

Surgical treatment is recommended for patients who are resistant or intolerant to DA therapy. Surgery may achieve biochemical remission, and tumor debulking might improve responsiveness to DA therapy in resistant cases, allowing dose reduction and mitigation of drug-related adverse effects [109]. Studies have shown that, in patients resistant to standard-dose cabergoline, a strategy of debulking surgery combined with medical therapy results in a greater reduction in serum PRL levels, higher biochemical remission rates (32.1% vs. 22.2%), and a lower required cabergoline dose (2.4 mg/week reduced to 1.4 mg/week) compared with dose escalation alone [112]. For patients with adenoma apoplexy accompanied by rapid or progressive visual decline, particularly when the tumor appears cystic or hemorrhagic on imaging, the anticipated efficacy of pharmacological treatment is poor, and prompt surgical intervention to relieve optic nerve compression may be considered [113].

#### Recommendation 24

In patients receiving DA therapy with suspected CSF rhinorrhea, nasal endoscopy should be considered, and measurement of β2-transferrin or β-trace protein may be performed in centers with appropriate facilities (evidence level: very low; strength of recommendation: weak). CSF rhinorrhea caused by the tumor or secondary to DA therapy warrants immediate surgical repair (Good Practice Statement).

Among patients receiving DA therapy, the incidence of CSF rhinorrhea is approximately 6.1%, occurring more frequently in male patients with invasive giant tumors [114]. Diagnosis can be established by nasal endoscopic examination or by detecting elevated levels of β2-transferrin or β-trace protein in nasal fluid [115]. A literature review of 36 cases of DA therapy–associated CSF rhinorrhea reported that 94% of cases involved invasive tumors, with a mean pretreatment tumor diameter of 3.6 cm. The mean baseline PRL level was 4917 ng/ml. CSF rhinorrhea most commonly occurred between 3 days and 4 months after initiation of therapy, although delayed onset up to 17 months has been reported in individual cases [116]. Accordingly, patients with invasive giant tumors should be fully informed of the risks of CSF rhinorrhea and secondary intracranial infection following DA therapy. Once CSF rhinorrhea is confirmed, DA therapy should be discontinued immediately and prompt surgical intervention undertaken, primarily aimed at histopathological confirmation and skull base reconstruction, followed by resumption of medical therapy postoperatively. Imaging examinations (e.g., high-resolution CT, CT cisternography, or MRI) are not recommended as first-line qualitative diagnostic tools for confirming CSF rhinorrhea, but they do have auxiliary diagnostic value; however, they may be used for precise leak localization when surgical intervention is planned. A literature review by Česák et al. [117] involving 60 cases of DA-associated CSF rhinorrhea demonstrated that leak repair was successful in 87.5% following endonasal or transcranial surgery, whereas 12.5% of cases resolved with conservative management, like DA withdrawal, strict bed rest, and lumbar drainage.

#### Recommendation 25

For women with macroadenomas who desire future fertility, debulking surgery may be considered as an alternative therapy in order to reduce the risk of symptomatic tumor enlargement during subsequent pregnancy (evidence level: low; strength of recommendation: weak).

A study comparing first-line medical therapy with primary surgical treatment in young female patients reported that, at the last follow-up (median follow-up duration of 90 months), 68% of patients in the surgical group achieved sustained long-term biochemical remission, whereas 64% of patients in the medically treated group required ongoing long-term therapy [118]. In addition, young women with reproductive aspirations may particularly benefit from surgical management, as experienced surgeons can achieve high remission rates while preserving gonadal function and facilitating restoration of fertility. In a retrospective analysis of 99 women of reproductive age, regular menstrual cycles were restored postoperatively in 76.5% of patients with microadenomas, and 82.3% of those with preoperative infertility successfully conceived and delivered after surgery [119]. For women with macroadenomas who desire pregnancy, debulking surgery performed prior to conception can effectively reduce the risk of symptomatic tumor enlargement during pregnancy. Previous studies have shown that symptomatic tumor growth occurred in 21% of macroadenoma patients managed with medical therapy alone, whereas the incidence was reduced to 4.7% among those who had undergone prior debulking surgery [120].

### Radiotherapy

#### Recommendation 26

Radiotherapy can be considered for prolactinomas under the following circumstances: (1) resistance and/or intolerance to DA therapy; (2) contraindications to general anesthesia for surgery, inability to achieve surgical resection, postoperative residue, or postoperative recurrence; (3) refractory or metastatic pituitary prolactinomas (evidence level: very low; strength of recommendation: weak).

Radiotherapy is one of the comprehensive treatment modalities for prolactinomas, including stereotactic radiosurgery (SRS) and external beam radiotherapy (EBRT), the latter including techniques such as 3D conformal radiotherapy and intensity-modulated radiotherapy. The local tumor control rate for prolactinomas treated with radiotherapy ranges from 68 to 100% [5, 120, 121], and the biochemical remission rate ranges from 30 to 80% [121–123]. The average time to PRL normalization takes 2–3 years, sometimes even 8–10 years or more [124, 125]. SRS involves delivering a high dose of radiation precisely to the target in a single or few (≤ 5) fractions or staged sessions under stereotactic guidance, more effectively killing tumor cells while better protecting surrounding normal tissues [123]. The time to endocrine remission after SRS such as Gamma Knife is shorter than with conventional EBRT (average 1–2 years). Except for tumors with extensive invasion, diffuse involvement of surrounding normal tissues, or metastatic prolactinomas, SRS is preferentially recommended [1, 24, 123, 126].

The 10–20-year cumulative risk of pituitary deficiency with conventional EBRT can exceed 50% [5]. Complication rates after Gamma Knife radiosurgery are lower than with conventional radiotherapy; the incidence of new pituitary deficiency ranges from 5 to 42%, peaking at 4–5 years, and the complication rate of optic and other cranial neuropathy is 1.0–4.5% [1, 24, 122, 123, 126]. Late adverse events after radiotherapy, such as cerebrovascular disease, neurocognitive dysfunction, and secondary tumors, are rare but cannot be ignored [5, 24, 126]. Before performing radiotherapy, patients should be informed about potential common adverse events, such as pituitary deficiency, optic and other cranial neuropathies; other rare complications such as CSF leakage, carotid artery injury, radiation-induced brain injury, or secondary brain tumors should also be fully disclosed, with early identification and appropriate management. Adverse events may even occur many years after radiotherapy, warranting lifelong follow-up.

#### Recommendation 27

Consider discontinuing DA 1–2 months before radiotherapy; if DA is needed after radiotherapy, it is suggested to restart DA 1 month after radiotherapy (evidence level: very low; strength of recommendation: weak).

Some retrospective studies demonstrated that DA may confer radioresistance to prolactinoma cells, potentially diminishing the efficacy of radiation, thus reducing the endocrine remission rate and prolonging the time to remission after radiotherapy [123, 126–130]. Therefore, discontinuation of DA during radiotherapy is suggested to maximize the benefit of radiotherapy intervention. Cohen-Inbar et al. [129] recommended discontinuing DA medications for 6–8 weeks around the time of Gamma Knife radiosurgery, while Hung et al. [130] advocated for temporary 6–8-week discontinuation of DA prior to SRS. However, in clinical practice, the dangers of a possible tumor regrowth after DA withdrawal (especially for invasive prolactinomas) should be fully balanced, requiring individualized management. PRL levels should be monitored regularly off DA treatment [124, 129, 131].

### High-risk prolactinomas

#### Recommendation 28

For male patients with prolactinomas who develop bromocriptine resistance, switching to cabergoline, combined with surgical debulking and/or radiotherapy, is recommended, along with close follow-up (evidence level: very low; strength of recommendation: weak).

Male prolactinoma is classified as one of the high-risk pituitary adenomas, among which macroadenomas are more common compared to females, and the incidence of giant adenomas is also higher [89, 132, 133]. A retrospective study involving 219 patients (145 females, 74 males) found that baseline PRL levels (2789 ± 573 ng/ml and average tumor diameter 26 ± 2 mm) in male patients were significantly higher than in females (292 ± 74 ng/ml and 10 ± 1 mm), with no correlation to age at diagnosis or symptom duration. The incidence of bromocriptine resistance (30% vs. 5%) and invasive macroadenomas (52% vs. 27%) were significantly higher in males than females [134, 135]. Some studies report male sex as a predictor of DA resistance in prolactinoma patients [136, 137]. For bromocriptine resistance, switching to cabergoline or combining with surgical debulking and/or radiotherapy is an option.

#### Recommendation 29

For patients with refractory or metastatic prolactinomas, a comprehensive treatment plan formulated by a MDT is recommended (Good Practice Statement).

#### Recommendation 30

The alkylating chemotherapeutic agent TMZ is recommended as the first-line chemotherapy regimen for refractory or metastatic pituitary prolactinomas. Treatment response should be assessed at 3 months, and the treatment course should be maintained for at least 6 months (evidence level: very low; strength of recommendation: weak).

Refractory pituitary tumors are defined as those with invasiveness (radiologically confirmed invasion of surrounding structures), resistance to standard therapies (persistent growth or recurrence after surgery, radiotherapy, and pharmacological treatment), rapid tumor growth in a short period, and histopathological features of high proliferative potential (Ki-67 proliferation index ≥ 3%, mitotic count > 2/10 HPF, positive p53 protein expression) [138, 139]. The diagnosis of metastatic pituitary neuroendocrine tumor applies when distant metastasis (intracranial or systemic) is present. Symptoms related to specific sites (neurological deficits, back pain, etc.) or a marked discrepancy between PRL level and pituitary tumor size should be evaluated for potential metastasis. Common metastatic sites include intracranial region, spine, liver, lymph nodes, bone, etc.

Treatment of refractory prolactinomas requires comprehensive assessment by a MDT to decide on cabergoline dose escalation, combined re-operation, or radiotherapy. If the tumor still progresses after standard therapy (DA, surgery, radiotherapy), TMZ is recommended as the first-line chemotherapeutic agent (ethics review and patient informed consent are required for off-label use). TMZ can improve overall and progression-free 5-year survival rates in responders, but only one-third of patients achieve complete or partial radiological response [139]. Assessment after 3 cycles can identify responders and non-responders, with responders advised to continue treatment for a total duration of at least 6 months [138]. A retrospective review of 94 patients with aggressive/metastatic prolactinomas treated with TMZ found that 58 (62%) had disease control or stabilization after TMZ treatment [140]. Patients receiving concurrent radiotherapy and TMZ had higher response rates [141]. Low O^6^-methylguanine-DNA methyltransferase (MGMT) expression (< 50%) suggests a higher likelihood of tumor response to TMZ (76% vs. 14%) [142]. The response rate in clinically functional pituitary tumors is higher than in non-functional tumors, independent of MGMT status. Among patients with complete response, partial response, and stable disease, progression occurred in 25%, 40%, and 48%, respectively, after a median follow-up of 12 months [141]. Mismatch repair protein deficiency (e.g., MSH6) may lead to TMZ resistance [143].

### Pregnancy

#### Recommendation 31

Women with microprolactinomas taking DA do not need to discontinue medication when attempting conception (evidence level: very low; strength of recommendation: weak). For women with macroadenomas, it is suggested to reduce the tumor size to that of a microadenoma before attempting conception (evidence level: very low; strength of recommendation: weak). For patients with resistant or persistently growing macroadenomas, surgical treatment followed by conception attempt is recommended (evidence level: very low; strength of recommendation: weak).

Pre-pregnancy patients should be assessed for the following conditions before attempting conception: PRL normalized, sustained shrinkage of the prolactinoma (traditionally reduced to microadenoma), resolution of visual field defects and other clinical symptoms. The timing of pregnancy in women with prolactinomas requires comprehensive assessment of disease control status, tumor size, and treatment response. Patients considering pregnancy should ideally achieve serum PRL normalization (resumption of regular menses also indicates PRL normalization), significant tumor volume reduction (especially for macroadenomas), and resolution of clinical symptoms. For microadenoma patients, the risk of tumor enlargement during pregnancy is only 1–5% [120], and they can attempt conception while taking DA medication [144–147].

For macroadenoma patients, treatment with DA or surgery is recommended beforehand, as studies show that treated macroadenoma patients have their risk of tumor enlargement during pregnancy reduced from 18 to 4.7% [148, 149]. Although serum PRL can decrease rapidly in medication-sensitive patients, with resumption of menses, tumor volume reduction takes time; therefore, barrier contraception is recommended during the initial treatment phase. Pre-pregnancy MRI should be performed to assess prolactinoma volume, and conception attempts should ideally begin only after the tumor has shrunk to microadenoma [149]. The spontaneous abortion rate in untreated hyperprolactinemic patients can reach 30–40%, decreasing to 10–20% after DA therapy, similar to the rate in the normal population [150]. The risk of tumor enlargement during pregnancy is significantly lower in patients who conceive after DA therapy compared to those inadequately treated before pregnancy [151].

For the management of pregnant patients with prolactinomas, MDT support before, during, and after surgery is crucial for optimizing maternal and fetal outcomes [1]. The MDT model has been shown to significantly improve outcomes in pregnant patients with pituitary adenomas. Based on current evidence, it is recommended that all pregnant women with prolactinomas, especially those with macroadenomas, or when tumor progression or apoplexy symptoms are detected, receive MDT support to ensure the best possible maternal and fetal outcomes [152].

Current evidence-based medicine indicates that DA use does not increase the risk of fetal miscarriage or malformations. Multiple large-scale clinical studies show that the fetal malformation rate associated with first-trimester bromocriptine exposure is 3.9%, not statistically different from the baseline risk of 2–4% in the general population. The spontaneous abortion rate with first-trimester bromocriptine exposure was 9.4%, not significantly different from the 8.1% rate in non-exposed women [153]. Cabergoline has been associated with a congenital malformation rate of 2.7% in international multicenter studies, with no specific pattern of malformations identified [154].

#### Recommendation 32

For pregnant women with microprolactinomas, discontinuation of DA therapy is recommended upon confirmation of pregnancy (evidence level: very low; strength of recommendation: weak).

For patients with microprolactinomas, current evidence and clinical guidelines generally recommend discontinuing DA upon confirmation of pregnancy, without the need to continue medication until the end of the first trimester. Multiple large cohort studies show that the risk of symptomatic tumor enlargement during pregnancy after discontinuation in microadenoma patients is only 1–5% [120], and there is no significant difference in pregnancy outcomes such as miscarriage or preterm birth rates between groups discontinuing in the first trimester and those continuing medication [1]. Microadenoma patients discontinuing medication only require clinical monitoring for symptoms like headache or visual changes; routine PRL monitoring or pituitary MRI during pregnancy is not necessary. Discontinuation in the first trimester for microadenoma patients does not increase maternal–fetal risks and reduces unnecessary drug exposure [148], but it is important to note that this recommendation applies only to patients whose tumors were controlled with DA therapy before pregnancy; cases not meeting treatment goals require individualized assessment and management.

#### Recommendation 33

For pregnant women with macroadenomas that were not effectively controlled before pregnancy, continuation of DA therapy throughout the pregnancy can be considered (evidence level: very low; strength of recommendation: weak).

For the pharmacological management of pregnant women with macroadenomas, an individualized strategy is recommended. DA use can be considered throughout pregnancy for macroadenoma patients not effectively controlled before pregnancy [146]. Multiple studies show that the risk of symptomatic tumor enlargement during pregnancy in untreated macroadenoma patients is as high as 15–35%, significantly higher than in microadenoma patients [120]. The European Society of Endocrinology guidelines recommend continuous DA therapy throughout pregnancy for patients with suprasellar extending macroadenomas, a history of tumor progression, or visual dysfunction [155]. During continuous treatment, the lowest effective dose (usually 50–70% of the pre-pregnancy dose) can be used, and the treatment plan should be re-evaluated 2 weeks after delivery [24].

#### Recommendation 34

Routine monitoring of serum PRL levels is not recommended for pregnant women with microadenomas during pregnancy (evidence level: very low; strength of recommendation: weak). For pregnant women with macroadenomas, if serum PRL levels rise suddenly during pregnancy (increase > 50% from baseline), it may suggest tumor growth, but this must be interpreted in conjunction with clinical symptoms (evidence level: very low; strength of recommendation: weak).

Regarding PRL monitoring strategies during pregnancy for prolactinoma patients, current evidence suggests differentiated approaches based on tumor size and clinical status. Microadenoma patients typically do not require routine PRL monitoring, as PRL rises physiologically during pregnancy (up to 10–20 times non-pregnant levels) and is not correlated with tumor activity [24]. Multiple studies show that even with significant PRL elevation (> 200 ng/ml or 4240 mU/L) in microadenoma patients, the risk of tumor progression remains below 5% [120]. For macroadenoma patients, PRL testing every 2–3 months is suggested. A sudden, significant rise in PRL (> 50% increase from baseline) may indicate tumor growth. PRL monitoring is more challenging in patients on continuous DA therapy, as the drug suppresses PRL secretion, potentially masking tumor growth. When symptoms like headache or visual dysfunction appear, imaging evaluation should be considered regardless of PRL level. It is suggested that microadenomas require only symptom monitoring; asymptomatic macroadenomas should have PRL checked every 3 months; those on medication or with symptomatic progression require combined PRL and imaging assessment. Note that different assay methods (e.g., macroprolactin interference) and individual variations affect result interpretation; using the same laboratory and method for serial testing is advised.

#### Recommendation 35

If significant tumor growth is suspected during pregnancy, accompanied by new symptoms such as headache, visual loss, or visual field defects, non-contrast MRI is recommended, and DA therapy should be restarted (evidence level: very low; strength of recommendation: weak). For cases unresponsive to medication or presenting with acute visual loss, impaired consciousness, or other critical conditions, surgery is a reasonable option (evidence level: very low; strength of recommendation: weak).

For pregnant patients with suspected tumor growth-related symptoms, a stepwise management strategy should be adopted based on symptom severity, gestational age, and tumor characteristics. When patients present with new headaches, visual impairment, or cranial nerve palsy, non-contrast pituitary MRI is the preferred imaging modality, its safety having been confirmed by multiple studies [156]. Physiological pituitary enlargement (height up to 10–12 mm) occurs in 15–20% of pregnant women and must be differentiated from true tumor growth. Regarding pharmacological treatment, DA remains the first choice. Studies show that 70–80% of cases show symptom improvement within 2–4 weeks after restarting or intensifying DA therapy [1]; even in the third trimester, the safety of DA therapy is considered superior to allowing tumor progression [155]. For the 20–30% of cases unresponsive to medication or those presenting with acute visual loss, impaired consciousness, or other critical situations, transsphenoidal pituitary surgery is a reasonable option, with higher safety for both mother and fetus if performed in the second trimester (13–27 weeks) [94]. Notably, 5–10% of invasive macroadenomas may require combined glucocorticoid therapy (especially when pituitary deficiency is present), but complications like gestational diabetes must be closely monitored [1].

#### Recommendation 36

Breastfeeding is encouraged postpartum. If DA drug therapy is required due to the medical condition, breastfeeding should be discontinued (evidence level: very low; strength of recommendation: weak).

Regarding postpartum lactation for prolactinoma patients, current clinical evidence indicates that the vast majority can breastfeed safely without significantly increasing the risk of tumor progression. Multiple studies confirm that physiological hyperprolactinemia induced by lactation (typically maintained at 50–200 ng/ml) is not clearly correlated with pathological tumor growth [1]. The incidence of tumor enlargement during 12 months of lactation is 1.2% for microadenoma and 4.3% for macroadenoma patients, both significantly lower than in non-lactating control groups [153, 157]. Regarding pharmacological intervention, 85–90% of microadenoma and 60–70% of macroadenoma patients do not require restarting DA therapy during lactation [155, 158]; however, for patients whose tumors were not controlled pre-pregnancy or who develop symptoms during lactation, DA remains the first-choice treatment. Regarding clinical monitoring strategy, breastfeeding while on DA therapy is not recommended.

### Summary and outlook

Due to limitations in the overall quality of evidence, some recommendations in this consensus have a low strength rating. Clinicians should develop individualized plans based on this consensus and specific circumstances in practice. Owing to limitations in medical resources, some tests and examinations recommended in this consensus may not be feasible in certain regions. Clinicians should adapt the recommendations to local practice realities. This consensus is based on literature and expert opinion, does not have legal force, and its content will be continuously updated with the evolution of medical evidence. Implementation should consider the specific clinical context comprehensively.

### Limitations of this consensus

Due to the overall limited quality of evidence, some recommendations in this consensus have a low strength rating but are still important for clinical practice. Clinicians should develop individualized plans based on this consensus and specific circumstances in practice. Owing to limitations in medical resources, some tests and examinations recommended in this consensus may not be feasible in certain regions. Clinicians should adapt the recommendations to local practice realities. This consensus is based on literature and expert opinion, does not have legal force, and its content will be continuously updated with the evolution of medical evidence. Implementation should consider the specific clinical context comprehensively.

## Supplementary Information

Supplementary Material 1.

Supplementary Material 2.

Supplementary Material 3.

Supplementary Material 4.

Supplementary Material 5.

## Data Availability

No datasets were generated or analysed during the current study.

## Abbreviations

PRL: Population prevalence of pituitary prolactin
CPASC: China Pituitary Adenoma Specialist Council
AGREE: Appraisal of Guidelines for Research and Evaluation
STAR: Applicability Rankings
RIGHT: Reporting Items for practice Guidelines in HealThcare
MDT: Multidisciplinary team
GRADE: Development and Evaluation
EtD: Evidence-to-Decision
IgG: Immunoglobulin G
PEG: Polyethylene glycol
MEN1: Multiple endocrine neoplasia type 1 gene
AIP: Aryl hydrocarbon receptor-interacting protein gene
MEN: Multiple endocrine neoplasia
MRI: Magnetic resonance imaging
DA: Dopamine agonist
CT: Computed tomography
T1WI: T1-weighted imaging
T2WI: T2-weighted imaging
CTA: CT angiography
MRA: MR angiography
SRS: Stereotactic radiosurgery
EBRT: External beam radiotherapy
HPA: Hypothalamic–pituitary–adrenal
HPT: Hypothalamic-pituitary-thyroid
TMZ: Temozolomide
