# Supplementary material for: Chinese consensus on the diagnosis and treatment of prolactinomas (2025 edition)
Source: Chin Neurosurg J. 2026 Jun 8;12:17. doi: 10.1186/s41016-026-00437-7 (PMC13248255; doi:10.1186/s41016-026-00437-7)
Supplement: Supplementary file 1 — Supplementary Material 1. [file 41016_2026_437_MOESM1_ESM.docx]

**Table 1 Definition of Certainty of Evidence Levels in This Consensus**

| **Certainty of Evidence** | **Definition** |
| --- | --- |
| High | The evidence is considered reliable and sufficient to support clinical decision-making; additional homogeneous studies are unlikely to change the results. |
| Moderate | The evidence is relatively reliable and can generally support clinical decision-making; additional homogeneous studies are unlikely to meaningfully change the results. |
| Low | The evidence is of limited reliability and can be considered as a reference for clinical decision-making; further studies are needed to confirm or refute its credibility. |
| Very Low | The evidence is unreliable and generally should not be used as a basis for clinical decision-making; further research is needed to support clinical decisions. |
