# Supplementary material for: Chinese consensus on the diagnosis and treatment of prolactinomas (2025 edition)
Source: Chin Neurosurg J. 2026 Jun 8;12:17. doi: 10.1186/s41016-026-00437-7 (PMC13248255; doi:10.1186/s41016-026-00437-7)
Supplement: Supplementary file 2 — Supplementary Material 2. [file 41016_2026_437_MOESM2_ESM.docx]

**Table 2 Definition of Strength of Recommendations in This Consensus**

| **Strength of Recommendation** | **Definition** |  |
| --- | --- | --- |
| Strong Recommendation For | For the vast majority of patients, the benefits of the test or intervention significantly outweigh the harms; the intervention should be adopted in most circumstances. |  |
| Weak Recommendation For | The benefits and harms of the intervention or test are approximately balanced, or the evidence is insufficient. Although the recommendation leans toward the intervention, clinicians and patients should engage in shared decision-making based on individual circumstances and preferences. |  |
| Weak Recommendation Against | The harms and benefits of the intervention or test are approximately balanced, or the evidence is insufficient. Although the recommendation leans toward the intervention, clinicians and patients should engage in shared decision-making based on individual circumstances and preferences. |  |
| Strong Recommendation Against | For the vast majority of patients, the risks of the test or intervention significantly outweigh the benefits; the intervention should not be adopted in most circumstances. |  |
| Good Practice Statement | The recommendation is of significant clinical importance but is not primarily based on empirical evidence, or the basis for the decision is not suitable for evaluation using the GRADE evidence quality assessment. |  |
|  | | |
