# Supplementary material for: Chinese consensus on the diagnosis and treatment of prolactinomas (2025 edition)
Source: Chin Neurosurg J. 2026 Jun 8;12:17. doi: 10.1186/s41016-026-00437-7 (PMC13248255; doi:10.1186/s41016-026-00437-7)
Supplement: Supplementary file 3 — Supplementary Material 3. [file 41016_2026_437_MOESM3_ESM.docx]

**Table 3 Aetiology of hyperprolactinaemia**

| **Physiological**  Pregnancy; breast or nipple stimulation; stress; sleep; coitus; exercise.  **Pathological**  **Hypothalamic-pituitary stalk damage**  Adenomas; craniopharyngioma; Rathke’s cleft cyst; suprasellar pituitary mass extension; meningioma; dysgerminoma; hypothalamic or pituitary metastases; granulomatous disorders; infiltrations; pituitary and/or brain irradiation; intracranial hypotension; trauma (pituitary stalk section, sellar surgery, severe head injury).  **Pituitary**  Prolactinoma; acromegaly; macroadenoma (compressive); idiopathic; plurihormonal adenoma; lymphocytic hypophysitis; parasellar mass.  **Non-pituitary disorders**  Ectopic prolactin secretion; primary hypothyroidism; chronic renal failure; cirrhosis; pseudocyesis; epileptic seizures; malnutrition; anorexia nervosa; chest (neurogenic, chest wall trauma, piercings, surgery, herpes zoster).  **Genetic**  Inactivating mutation in the gene encoding prolactin receptor (*PRLR*).  **Pharmacological**  **Dopamine receptor blockers**  Phenothiazines (chlorpromazine, perphenazine); butyrophenones (haloperidol); thioxanthenes; metoclopramide; domperidone; alizapride.  **Dopamine synthesis inhibitors**  α-Methyldopa.  **Catecholamine depleters**  Reserpine.  **Cholinergic agonists**  Physostigmine.  **Antihypertensives**  Labetalol; reserpine; verapamil.  **H2 antihistamines**  Cimetidine; ranitidine.  **Oestrogens**  Oral contraceptives (controversial).  **Anticonvulsants**  Phenytoin.  **Neuroleptics**  Chlorpromazine; risperidone; promazine; promethazine; trifluoperazine; fluphenazine; butaperazine; perphenazine; thiethylperazine; thioridazine; haloperidol; pimozide; thiothixene; molindone.  **Opiates and opiate agonists**  Heroin; methadone; apomorphine; morphine.  **Antidepressants**  Tricyclic antidepressants; selective serotonin reuptake inhibitors. |
| --- |
