# Supplementary material for: Chinese consensus on the diagnosis and treatment of prolactinomas (2025 edition)
Source: Chin Neurosurg J. 2026 Jun 8;12:17. doi: 10.1186/s41016-026-00437-7 (PMC13248255; doi:10.1186/s41016-026-00437-7)
Supplement: Supplementary file 4 — Supplementary Material 4. [file 41016_2026_437_MOESM4_ESM.docx]

**Table 4 Ptuitary tumor Knosp classification**

| **Knosp Grade** | **Definition** | **Imaging Landmarks (Coronal View)** |
| --- | --- | --- |
| Grade 0 | Normal morphology of the intracranial cavernous sinus with enhancement of the cavernous venous plexus. The tumor does not extend beyond the medial tangent line of the intracavernous internal carotid artery (ICA) segments C2-C4. | Tumor margin does not cross the tangent line along the medial wall of the intracavernous ICA. |
| Grade 1 | Tumor extends beyond the medial tangent line of the ICA C2-C4 segments but not beyond the intercarotid line (center line). The medial cavernous sinus venous plexus is absent. | Tumor crosses the medial wall tangent of the ICA but does not extend beyond the line connecting the centers of the bilateral ICA lumens (i.e., the line between the centers of the left and right ICAs). |
| Grade 2 | Tumor extends beyond the intercarotid line (center line) but not beyond the lateral tangent line of the ICA C2-C4 segments. May be accompanied by absence of the superior and/or inferior cavernous sinus venous plexuses. | Tumor extends beyond the line connecting the ICA centers but does not cross the tangent line along the lateral wall of the ICA. |
| Grade 3 | Encasement of the ICA (Subdivided into 3A and 3B). | Grade 3A: Tumor extends laterally beyond the lateral wall tangent of the ICA but does not fully encircle the ICA.  Grade 3B: Tumor extends inferiorly, invading the inferior compartment of the cavernous sinus (e.g., into the sphenoid sinus). |
| Grade 4 | Complete encasement and narrowing of the ICA | Tumor completely encases the intracavernous segment of the ICA, resulting in narrowing of the ICA lumen; OR tumor extends superiorly, completely encasing the supraclinoid (paraclinoid) segment of the ICA. |
