# Supplementary material for: Chinese consensus on the diagnosis and treatment of prolactinomas (2025 edition)
Source: Chin Neurosurg J. 2026 Jun 8;12:17. doi: 10.1186/s41016-026-00437-7 (PMC13248255; doi:10.1186/s41016-026-00437-7)
Supplement: Supplementary file 5 — Supplementary Material 5. [file 41016_2026_437_MOESM5_ESM.docx]

**GRADE Evidence Table**

Legend of Evidence Quality**：**

| **High** | **Moderate** | **Low** | **Very low** |
| --- | --- | --- | --- |

# **Part 1: Diagnosis and Differential Diagnosis**

**Recommendation 1: Serum prolactin measurement is recommended in all patients with any clinical suspicion:(1) Child-bearing aged females presenting with menstrual abnormalities (irregularity, amenorrhea, or prolonged menstrual cycles), infertility, and/or galactorrhea;(2) Males with decreased libido, erectile dysfunction, infertility, gynecomastia, and/or galactorrhea;(3) Adolescents with delayed or arrested puberty, and children or adolescents with growth retardation;(4) Imaging examinations suggestive of a sellar region lesion.(Evidence level: Low; Strength of recommendation: Strong)**

(Despite low-quality direct evidence for testing in each specific scenario, the panel made a strong recommendation based on the well-established pathophysiological link between hyperprolactinemia and these clinical presentations, the low risk and cost of the test, and the potential serious consequences of missing a diagnosis of prolactinoma)

**Recommendation 1a: Measure serum prolactin (PRL) levels in women of reproductive age with menstrual abnormalities (irregular menses, amenorrhea, prolonged cycles), infertility, and/or galactorrhea (Low certainty evidence).**

| Study ID | Study Design | Population | Risk of Bias | Indirectness | Inconsistency | Publication Bias | Certainty of Evidence | Findings |
| --- | --- | --- | --- | --- | --- | --- | --- | --- |
| Touraine P, et al. 2001 [1] | Retrospective, single-centre cohort study | Women with hyperprolactinaemia | Not serious | Not serious | Not serious | Not serious | Low | This study predominantly included young women presenting with menstrual disturbance (92%). Serum prolactin (PRL) levels were significantly higher in patients with amenorrhoea (165.1±30.1 ng/ml) compared to those with oligomenorrhoea (73.1±6.0 ng/ml). Correspondingly, pituitary tumours were larger in the amenorrhoeic group. Pituitary adenomas were documented in 60% of participants, with microadenomas (64%) being more common than macroadenomas (36%). A positive correlation between PRL concentration and tumour size was observed.  The evidence is of **low certainty** due to the observational nature of the study. Based on the low certainty evidence, testing for high prolactin **may be** considered in women of reproductive age who have menstrual problems, infertility, or unwanted milk production. |
| Reindollar RH, et al. 1986 [2] | Retrospective, single-centre cohort study | Women with secondary amenorrhoea | Not serious | Not serious | Not serious | Not serious | Low | This study evaluated women with secondary amenorrhoea (mean age at onset 26.4 years). The most common causes were hypothalamic suppression (33.5%), chronic anovulation with estrogen excess (28%), hyperprolactinaemia (14%), and ovarian failure (12%). A notable proportion of women (48%) with hyperprolactinaemia presented with drug-induced amenorrhoea. Conversely, among those with drug-related amenorrhoea, 17% also had hyperprolactinaemia.  In women who have stopped having periods, hyperprolactinaemia (high prolactin) was identified as a common cause. There was a notable overlap between drug-induced amenorrhoea and high prolactin levels. Based on the **low certainty evidence**, testing for high prolactin **may be** considered in women of reproductive age who have stopped menstruating. |
| Lee DY， et al. 2012 [3] | Retrospective, single-centre cohort study | Adolescents and young women with menstrual disorders | Not serious | Not serious | Not serious | Not serious | Low | In this study, participants were stratified into two age groups: 11-20 years (Group I) and 21-30 years (Group II). The prevalence of hyperprolactinaemia was low in both groups among those with primary amenorrhoea or oligomenorrhoea. In contrast, hyperprolactinaemia was a comparatively frequent cause of secondary amenorrhoea, with a significantly higher prevalence in Group II (13.8%) than in Group I (5.5%) (P = 0.001). Prolactinomas were also more common in Group II (P = 0.015). Among women presenting with abnormal uterine bleeding, hyperprolactinaemia was detected more frequently in Group II (9.4% vs. 2.6%, P < 0.001), although the distribution of other aetiological factors was similar between groups.  Based on the **low certainty evidence**, testing for high prolactin **may be** considered in adolescents and young women with menstrual disorders. |
| Zhang L, et al,2023 [4] | Retrospective, cross-sectional, single-centre study | Women with recurrent pregnancy loss (RPL) | Not serious | Serious | Not serious | Not serious | Very low | This study of women with recurrent pregnancy loss (RPL) reported a hyperprolactinaemia prevalence of 17.34% (85 out of 490 participants), indicating that a notable proportion of this high-risk cohort exhibits prolactin abnormalities.  The study population consisted exclusively of women with RPL. This population differs meaningfully from the broader target population addressed in the recommendation, which includes all women of reproductive age with menstrual disturbances or infertility. Thus, the certainty of the evidence was rated down by one level for **indirectness**.  Based on the **very low certainty evidence**, we **are very uncertain** about whether testing for high prolactin is useful in these women. |

**Recommendation 1b: Measure serum prolactin (PRL) levels in Men with unexplained decreased libido, erectile dysfunction, infertility, gynecomastia, or galactorrhea (Low certainty evidence).**

| Study ID | Study Design | Population | Risk of Bias | Indirectness | Inconsistency | Publication Bias | Certainty of Evidence | Findings |
| --- | --- | --- | --- | --- | --- | --- | --- | --- |
| Ke X, et al,2024 [5] | Retrospective, single-centre cohort study | Men with prolactin-secreting pituitary adenomas | Not serious | Not serious | Not serious | Not serious | Low | This cohort study of men with prolactinomas reported that the most common clinical manifestations were sexual dysfunction (41.7%), visual disturbances (35.8%), headache (35.0%), and gynaecomastia (18.9%). Among these, the symptoms relevant to the recommendation were frequently observed.  Based on this **low certainty evidence**, measuring prolactin levels **may be** considered in men with unexplained decreased libido, erectile dysfunction, infertility, gynaecomastia, or galactorrhoea. |

**Recommendation 1c: Measure serum prolactin (PRL) levels in adolescents with delayed or arrested puberty, or children/adolescents exhibiting growth retardation (Very low certainty evidence).**

| Study ID | Study Design | Population | Risk of Bias | Indirectness | Inconsistency | Publication Bias | Certainty of Evidence | Findings |
| --- | --- | --- | --- | --- | --- | --- | --- | --- |
| Yang Y et al,2024 [6] | Retrospective, single-centre cohort study | Children and adolescents with prolactin-secreting pituitary adenomas | Serious | Not serious | Not serious | Not serious | Very low | This retrospective, single-centre cohort study of 170 pediatric and adolescent patients with prolactinomas identified distinct sex-specific clinical patterns. The majority of girls presented with menstrual disturbances (86.7%). Among boys, the most common presentations were headache (42.6%), decelerated linear growth (25.9%), and delayed puberty (18.2%).  The certainty of the evidence was rated down by one level for **risk of bias**. The study was a retrospective, single-centre review, which introduces a high likelihood of selection bias and information bias. The way patients were identified and data were collected may not reliably represent all children and adolescents with these symptoms.  Because of serious concerns about how this study was conducted, we **are very uncertain** about whether measuring prolactin is helpful in children or adolescents with growth delay or delayed puberty. |

**Recommendation 1d: Measure serum prolactin (PRL) levels in patients with imaging findings suggestive of a sellar/parasellar pituitary lesion.**

| Study ID | Study Design | Population | Risk of Bias | Indirectness | Inconsistency | Publication Bias | Certainty of Evidence | Findings |
| --- | --- | --- | --- | --- | --- | --- | --- | --- |
| Kreutz J 2015 [7] | Retrospective cohort study | Patients with confirmed prolactinomas | Serious | Serious | Not serious | Not serious | Very low | This retrospective cohort study of 82 patients with histologically verified prolactinomas (41 males, 41 females) reported that all lesions were initially detected by pituitary MRI. The study found that specific MRI signal characteristics (on T2-weighted imaging) were significantly associated with patient sex, tumor size, and serum prolactin levels (P < 0.001). The authors concluded that MRI was essential for excluding other causes of hyperprolactinaemia and for confirming the diagnosis.  The certainty of the evidence was rated down by two levels in total:  **Risk of bias:** The study had a retrospective design with inherent risks of selection and information bias, limiting the reliability of its conclusions.  **Indirectness:** The study population consisted entirely of patients with confirmed prolactinomas. This creates a significant indirectness issue when applying the evidence to the target population of the recommendation, which is all patients with imaging findings merely suggestive of a pituitary lesion.  **Very low certainty evidence** showed that in patients already diagnosed with a prolactinoma, MRI was used to find the tumor and its features were linked to prolactin levels. We **are very uncertain** about using MRI findings alone to decide when to measure prolactin in patients with a possible pituitary lesion seen on imaging. |

References:

1. Touraine P, Plu-Bureau G, Beji C, et al. Long-term follow-up of 246 hyperprolactinemic patients[J]. Acta Obstet Gynecol Scand, 2001, 80 (2): 162-8. doi: 10.1034/j.1600-0412.2001.080002162.x.
2. Reindollar R H, Novak M, Tho S P, et al. Adult-onset amenorrhea: a study of 262 patients[J]. Am J Obstet Gynecol, 1986, 155 (3): 531-43. doi: 10.1016/0002-9378(86)90274-7.
3. Lee D Y, Oh Y K, Yoon B K, et al. Prevalence of hyperprolactinemia in adolescents and young women with menstruation-related problems[J]. Am J Obstet Gynecol, 2012, 206 (3): 213.e1-5. doi: 10.1016/j.ajog.2011.12.010.
4. Zhang L, Du Y, Zhou J, et al. Diagnostic workup of endocrine dysfunction in recurrent pregnancy loss: a cross-sectional study in Northeast China[J]. Front Endocrinol (Lausanne), 2023, 14 1215469. doi: 10.3389/fendo.2023.1215469.
5. Ke X, Chen X, Wang L, et al. Experience in the Treatment of Male Prolactinomas: A Single-Center, 10-Year Retrospective Study[J]. Neuroendocrinology, 2024, 114 (12): 1077-89. doi: 10.1159/000541495.
6. Yang Y, Ke X, Duan L, et al. Clinical Characteristics and Outcomes of Prolactinomas in Children and Adolescents: A Large Retrospective Cohort Study[J]. J Clin Endocrinol Metab, 2024, 109 (9): e1741-e9. doi: 10.1210/clinem/dgad769.
7. Kreutz J, Vroonen L, Cattin F, et al. Intensity of prolactinoma on T2-weighted magnetic resonance imaging: towards another gender difference[J]. Neuroradiology, 2015, 57 (7): 679-84. doi: 10.1007/s00234-015-1519-3.

**Recommendation 2: For patients with hyperprolactinemia whose clinical manifestations do not match the serum PRL level or who lack related clinical manifestations, screening for macroprolactinemia is recommended. (Evidence grade: Low, weak recommendation)**

| Study ID | Study Design | Population | Risk of Bias | Indirectness | Inconsistency | Publication Bias | Certainty of Evidence | Findings |
| --- | --- | --- | --- | --- | --- | --- | --- | --- |
| Bayraktar N，2024 [1] | Retrospective, multicentre cohort study | Patients diagnosed with hyperprolactinaemia | Not serious | Not serious | Not serious | Not serious | Low | This cohort study explains that macroprolactin, a high molecular weight complex, is biologically inactive and thus does not cause the typical symptoms of hyperprolactinaemia (e.g., amenorrhoea, galactorrhoea, decreased libido). It notes that conventional immunoassays cannot differentiate macroprolactin from active, monomeric prolactin, leading to a condition termed pseudohyperprolactinaemia in patients with elevated serum prolactin but no corresponding clinical manifestations. The authors advocate for routine laboratory screening for macroprolactin in all hyperprolactinaemic samples and enhanced clinician collaboration to improve diagnostic accuracy.  Based on **low certainty evidence**, screening for macroprolactin **may be** considered in patients with high prolactin levels who do not have the expected symptoms. |
| Sharma LK, et al ,2021 [2] | Retrospective, multicentre cohort study | Patients diagnosed with hyperprolactinaemia | Not serious | Not serious | Not serious | Not serious | Low | This retrospective cohort study reported a prevalence of macroprolactinaemia of 13.7% among patients with hyperprolactinaemia. The study highlights the utility of screening for macroprolactin in patients who are asymptomatic or whose symptoms do not correlate with elevated serum prolactin levels. The authors suggest that such screening can effectively identify pseudohyperprolactinaemia and prevent unnecessary treatment.  Based on **low certainty evidence**, screening for macroprolactin **may be** considered in patients who have no symptoms or whose symptoms do not match their prolactin level, as it can help avoid unneeded treatment. |
| Ke X, et al 2023 [3] | Cross-sectional diagnostic accuracy study | Patients with hyperprolactinaemia and healthy controls | Not serious | Serious | Not serious | Not serious | Very low | This study reported that the prevalence of macroprolactinaemia among hyperprolactinaemic patients was 7.45% when measured by gel-filtration chromatography (GFC) and 5.32% by polyethylene glycol (PEG) precipitation. The study specifically enrolled patients whose clinical symptoms were discordant with their serum prolactin levels. Among this targeted subgroup (n=23), the prevalence of macroprolactinaemia confirmed by GFC was 21.7% (5/23), significantly higher than in patients without such discordance (P < 0.05). The authors concluded that patients with macroprolactinaemia typically present with atypical or absent symptoms, only modest prolactin elevation, and often have normal pituitary imaging or only a microadenoma. They recommended that PEG precipitation screening be reserved for patients in whom macroprolactinaemia is clinically suspected.  The certainty of the evidence was rated down by one level for **indirectness**. The study population was specifically selected to include only patients with a discordance between symptoms and prolactin levels. While this directly supports screening in that specific subgroup, it provides less direct evidence for applying the recommendation to the broader population of all “patients with hyperprolactinaemia whose clinical manifestations do not match the serum PRL level or who lack related clinical manifestations”, as some in this broader group may not have been as clearly discordant.  **Very low certainty evidence** showed that a harmless form of high prolactin (macroprolactin) was more common in patients whose symptoms did not match their prolactin level. We **are very uncertain** about whether all patients with high prolactin but no symptoms need this specific screening test, as the study focused on a highly selected group. |
| Hu Y, et al,2021 [4] | Cross-sectional prevalence study | Patients with hyperprolactinaemia | Not serious | Not serious | Not serious | Not serious | Low | This cross-sectional study of 1,140 patients with hyperprolactinaemia specifically enrolled individuals whose clinical presentation was inconsistent with serum prolactin levels. Within this cohort, the prevalence of macroprolactinaemia was 22.9% (261/1,140). Prevalence was highest (34.4%) among patients with total prolactin levels below 50 ng/mL. Patients with macroprolactinaemia typically presented with mild or atypical symptoms (e.g., isolated menstrual irregularity in 38.1%), whereas those with true hyperprolactinaemia more frequently exhibited galactorrhoea, amenorrhoea, or visual disturbances. Microadenomas were detected in only 17% of macroprolactinaemia cases, compared to 59.7% in true hyperprolactinaemia. The authors recommend routine screening for macroprolactin in hyperprolactinaemic patients with atypical features, unknown etiology, or negative pituitary imaging.  **Low certainty evidence** showed that in patients whose symptoms did not match their high prolactin level, about 23 out of 100 had a harmless form (macroprolactin). This was especially common when prolactin levels were only slightly high. These patients often had mild symptoms and usually did not have a pituitary tumor on imaging. Based on this low certainty evidence, screening for macroprolactin **may be** considered in patients with high prolactin levels who have symptoms that are not typical. |
| Che Soh NAA, et al ,2020 [5] | Systematic review and meta-analysis | Patients with hyperprolactinaemia | Not serious | Not serious | Not serious | Not serious | Low | This systematic review and meta-analysis of 67 international studies highlights the clinical and diagnostic implications of macroprolactinaemia. It reports a pooled prevalence of 18.9% among all hyperprolactinaemic samples. The review explains that macroprolactin has minimal biological activity and thus rarely causes classic hyperprolactinaemia symptoms (e.g., amenorrhoea, galactorrhoea). This leads to common scenarios of elevated prolactin without symptoms or symptoms disproportionate to prolactin levels, which should raise suspicion for macroprolactinaemia. The authors emphasize that clinical features alone cannot reliably distinguish true hyperprolactinaemia from pseudohyperprolactinaemia caused by macroprolactin, and that undetected macroprolactinaemia can lead to unnecessary investigations and treatments. They advocate for routine screening using the polyethylene glycol (PEG) precipitation assay.  Based on this **low certainty evidence**, screening for macroprolactin **may be** considered in patients whose symptoms do not match their prolactin level, as it can prevent unneeded tests and treatments. |

References:

1. Bayraktar N. The frequency of macroprolactinemia among patients with hyperprolactinemia in a central laboratory of a training and research hospital[J]. North Clin Istanb, 2024, 11 (6): 520-4. doi: 10.14744/nci.2023.94758.
2. Sharma L K, Dutta D, Sharma N, et al. Prevalence of Macroprolactinemia in People Detected to Have Hyperprolactinemia[J]. J Lab Physicians, 2021, 13 (4): 353-7. doi: 10.1055/s-0041-1732490.
3. Ke X, Wang L, Duan L, et al. Comparison of PEG precipitation and ultrafiltration treatment for serum macroprolactin in Chinese patients with hyperprolactinemia[J]. Clin Chim Acta, 2023, 544 117358. doi: 10.1016/j.cca.2023.117358.
4. Hu Y, Ni J, Zhang B, et al. Establishment of reference intervals of monomeric prolactin to identify macroprolactinemia in Chinese patients with increased total prolactin[J]. BMC Endocr Disord, 2021, 21 (1): 197. doi: 10.1186/s12902-021-00861-z.
5. Che Soh NAA, Yaacob NM, Omar J, et al. Global Prevalence of Macroprolactinemia among Patients with Hyperprolactinemia: A Systematic Review and Meta-Analysis. Int J Environ Res Public Health. 2020;17(21):8199. Published 2020 Nov 6. doi:10.3390/ijerph17218199.

**Recommendation 3: For patients with pituitary macroadenomas and normal or mildly elevated PRL but clinical manifestations suggestive of hyperprolactinemia, dilution of the serum sample and re-measurement of PRL are suggested to avoid missing the diagnosis of prolactinoma (excluding the hook effect). (Evidence grade: Low, Weak Recommendation). For patients with serum PRL levels above the detectable range (reporting >200 ng/ml or 4240 mU/L), dilution and measurement to obtain an absolute value are suggested for monitoring treatment efficacy. (Evidence grade: Low, Weak Recommendation)**

**3a: For patients with pituitary macroadenomas and normal or mildly elevated PRL but clinical manifestations suggestive of hyperprolactinemia, dilution of the serum sample and re-measurement of PRL are suggested to avoid missing the diagnosis of prolactinoma (excluding the hook effect). (Low certainty evidence)**

| Study ID | Study Design | Population | Risk of Bias | Indirectness | Inconsistency | Publication Bias | Certainty of Evidence | Findings |
| --- | --- | --- | --- | --- | --- | --- | --- | --- |
| Mahmoud MM et al ,2025 [1] | Systematic review | Macroprolactinomas in which the “hook effect” was confirmed | Not serious | Not serious | Not serious | Not serious | Low | This systematic review identified a cohort of 61 patients with macroprolactinoma in whom the “hook effect” was confirmed. In these patients, the median initial (undiluted) serum prolactin (PRL) level was only 108 ng/mL (IQR 76.6). However, upon serial dilution, the median PRL level rose significantly to 38,526 ng/mL. The smallest adenoma volume associated with the hook effect was 3.4 cm³, suggesting that no specific tumor size threshold can reliably rule out this artifact. Among a subset of ten macroprolactinomas from the original cohort, six were initially misdiagnosed as non-functioning pituitary adenomas (NFPAs) due to “normal” or “mildly elevated” reported PRL levels; all were correctly re-classified as prolactinomas after dilutional reassay.  **Low certainty evidence** indicates that in patients with a macroadenoma presenting with clinical manifestations of hyperprolactinaemia but normal or only mildly elevated serum PRL levels, reassaying the sample at high dilution **may be** considered to identify a potential hook effect and avoid misdiagnosis of a prolactinoma. |

**3b: For patients with serum PRL levels above the detectable range (reporting >200 ng/ml or 4240 mU/L), dilution and measurement to obtain an absolute value are suggested for monitoring treatment efficacy. (Low certainty evidence)**

| Study ID | Study Design | Population | Risk of Bias | Indirectness | Inconsistency | Publication Bias | Certainty of Evidence | Findings |
| --- | --- | --- | --- | --- | --- | --- | --- | --- |
| Mahmoud MM et al ,2025 [1] | Systematic review | Macroprolactinomas in which the “hook effect” was confirmed | Not serious | Not serious | Not serious | Not serious | Low | This systematic review, focusing on macroprolactinomas with a confirmed hook effect, reported that all enrolled cases underwent serial dilution to obtain absolute prolactin values. Following dilution, serum PRL concentrations as high as 577,000 ng/mL were documented. These accurate, absolute PRL values were then utilized for monitoring treatment response.  **Low certainty evidence** indicates that for patients with serum PRL levels exceeding the upper detection limit of the assay (e.g., reported as >200 ng/mL or >4240 mU/L), performing dilution to obtain an absolute PRL value **may be** considered to facilitate accurate monitoring of treatment efficacy. |

References:

1. Mahmoud M M, Haj-Ahmad L M, Sweis N W G, et al. Clinical Features and Hormonal Profile of Macroprolactinomas Presenting With the Hook Effect: A Systematic Review[J]. Endocr Pract, 2025, 31 (2): 215-25. doi: 10.1016/j.eprac.2024.11.002.

**Recommendation 4: A detailed history and physical examination should be performed for patients with hyperprolactinemia. Physiological, pharmacological, and other pathological causes not due to pituitary prolactinoma must be excluded before diagnosing a prolactinoma (Good Practice Statement).**

**Rationale for Good Practice Statement:**

1.Clarity and feasibility: The required action is unambiguous: omitting this step and proceeding directly to a diagnosis of prolactinoma constitutes an unequivocal clinical error.

2.Necessity: Without explicit guidance, some clinicians may bypass systematic evaluation and immediately order costly imaging or initiate unnecessary dopamine-agonist therapy.

3.Large, certain net benefit: Precise identification of the underlying aetiology maximises therapeutic benefit while minimising risk and cost; history-taking and physical examination are inexpensive, non-invasive, and immediately available.

4.Prohibitive opportunity cost of direct evidence: Pathophysiological and observational data are already abundant; placebo-controlled RCTs withholding basic clinical assessment would be unethical. A formal GRADE synthesis would require pooling heterogeneous data on hundreds of drugs and comorbidities—an impractical undertaking.

5.Indirect evidence chain

•Mechanistic studies and observational data confirm that numerous medications and systemic disorders raise prolactin.

•Structured medication histories have high sensitivity for identifying drug-induced hyperprolactinaemia.

•Withdrawal or substitution of the offending agent often normalises prolactin, obviating further intervention.

•Misattribution to prolactinoma risks unnecessary surgery or prolonged exposure to dopamine-agonist side effects.

**Recommendation 5: Genetic mutation testing is recommended for the following high-risk groups, prioritizing screening for mutations in the Multiple Endocrine Neoplasia type 1 (MEN1) gene and the Aryl Hydrocarbon Receptor Interacting Protein (AIP) gene: (1) Young patients (especially age <20 years) with macroadenomas (particularly giant adenomas ≥4 cm); (2) Those with a family history of Familial Isolated Pituitary Adenoma (FIPA) or Multiple Endocrine Neoplasia (MEN). (Evidence grade: Low, Weak Recommendation)**

| Study ID | Study Design | Population | Risk of Bias | Indirectness | Inconsistency | Publication Bias | Certainty of Evidence | Findings |
| --- | --- | --- | --- | --- | --- | --- | --- | --- |
| Boukerrouni A 2023 [1] | Retrospective cohort study | Patients with apparently sporadic prolactinomas | Not serious | Not serious | Not serious | Not serious | Low | This retrospective cohort study analysed germline DNA from 506 consecutive patients with apparently sporadic prolactinomas. Pathogenic or likely pathogenic variants (LPVs) were identified in 2.8% (14/506) of the entire cohort, exclusively involving the MEN1 (n=8) and AIP (n=6) genes. All variant carriers were diagnosed with macroprolactinoma before the age of 30. Among the subgroup of 258 patients with macroprolactinomas diagnosed before age 30, the LPV rate was 4.3% (11/258), rising to 10% in those under 18. Among the 20 patients with a documented family history of FIPA or MEN, the LPV rate was 15% (3/20), which was significantly higher than in sporadic cases (OR = 6.7, P = 0.023).  Low certainty evidence suggests that in high-risk groups, such as young patients (particularly <30 years) with macroprolactinomas and/or those with a family history of pituitary adenoma or MEN, genetic testing **may be** considered and should prioritise screening for mutations in the MEN1 and AIP genes. |
| Kumar S 2022 [2] | Retrospective cohort study | Children and adolescents (≤20 years) with giant prolactinomas | Not serious | Not serious | Not serious | Not serious | Low | This retrospective cohort study focused on children and adolescents (≤20 years) with giant prolactinomas (≥4 cm). Among the 95 patients in this high-risk group, pathogenic mutations were identified in 21.9% (7/32) for the MEN1 gene and 18.8% (6/32) for the AIP gene, with all AIP variants occurring in males. The study did not include patients with low-risk microadenomas, and therefore provides no data supporting genetic testing in that population.  Low certainty evidence from a study of very high-risk patients (children/adolescents with giant prolactinomas) suggests that in young patients (especially <20 years) with macroadenomas, genetic testing for mutations in the MEN1 and AIP genes **may be** considered. |

References:

1. Boukerrouni A, Cuny T, Anjou T, et al. Genetic testing in prolactinomas: a cohort study[J]. Eur J Endocrinol, 2023, 189 (6): 567-74. doi: 10.1093/ejendo/lvad148.
2. Kumar S, Sarathi V, Lila A R, et al. Giant prolactinoma in children and adolescents: a single-center experience and systematic review[J]. Pituitary, 2022, 25 (6): 819-30. doi: 10.1007/s11102-022-01250-y.

# **Part 2: Imaging**

**Recommendation 6: Patients diagnosed with hyperprolactinemia (after excluding physiological, pharmacological, and other systemic causes) should undergo pituitary magnetic resonance imaging (MRI). (Evidence grade: Low, Weak Recommendation). Pituitary dynamic contrast-enhanced MRI is recommended to improve the detection rate and localization accuracy of prolactin microadenomas. (Evidence grade: Very Low, Weak Recommendation)**

**6a: Patients diagnosed with hyperprolactinemia (after excluding physiological, pharmacological, and other systemic causes) should undergo pituitary magnetic resonance imaging (MRI) (Low certainty evidence).**

| Study ID | Study Design | Population | Risk of Bias | Indirectness | Inconsistency | Publication Bias | Certainty of Evidence | Findings |
| --- | --- | --- | --- | --- | --- | --- | --- | --- |
| Kreutz J 2015 [1] | Retrospective cohort study | 82 consecutive patients with biochemically confirmed prolactinomas (41 males, 41 females) | Not serious | Not serious | Not serious | Not serious | Low | This retrospective cohort study reported that all lesions were identified by pituitary MRI. MRI signal characteristics were significantly associated with patient sex, tumor size, and serum prolactin (PRL) levels. Male patients had larger tumors and higher PRL levels than female patients. The authors concluded that MRI was essential for excluding alternative diagnoses and confirming a prolactinoma.  The overall certainty of the evidence was rated as low. This initial rating reflects that the evidence is derived from a single, non-randomized observational study (retrospective cohort design) and is based on a selected cohort of patients with an already established diagnosis. No further upgrading or downgrading was applied based on the provided information.  Low certainty evidence suggests that in a cohort of patients with biochemically confirmed prolactinomas, pituitary MRI **may be** associated with the identification of all lesions, and tumor characteristics (such as size and signal) may correlate with clinical parameters (patient sex, serum prolactin levels) |
| Manuchehri A M 2007 [2] | Prospective study | Patients with hyperprolactinaemia and healthy volunteers | Not serious | Not serious | Not serious | Not serious | Low | This prospective study utilized both conventional and dynamic contrast-enhanced magnetic resonance imaging (DCE-MRI) in 23 patients with hyperprolactinaemia. Imaging identified unequivocal pituitary adenomas in 16 patients (5 macroadenomas, 11 microadenomas), while the remaining 7 patients had no visible lesion, a finding consistent with non-tumoural hyperprolactinaemia. The authors concluded that MRI can confirm the presence of a prolactinoma and help identify non-neoplastic causes of hyperprolactinaemia.  The overall certainty of the evidence was rated as low. This initial rating reflects that the evidence is derived from a single, small, prospective cohort study. No further upgrading or downgrading was applied based on the provided information.  **Low certainty evidence** from a small, prospective study suggests that in patients with hyperprolactinaemia, magnetic resonance imaging (MRI) **may** help identify the presence of a pituitary adenoma in some patients, while in others it **may suggest** a non-tumoural cause for the condition. The diagnostic performance and clinical impact of routine MRI in all patients with hyperprolactinaemia remain unclear. |

**6b: Whenever feasible, dynamic contrast-enhanced pituitary MRI is preferred to improve the detection rate and anatomical localisation of prolactin-secreting microadenomas (Very low certainty evidence, weak recommendation).**

| Study ID | Study Design | Population | Risk of Bias | Indirectness | Inconsistency | Publication Bias | Certainty of Evidence | Findings |
| --- | --- | --- | --- | --- | --- | --- | --- | --- |
| Manuchehri A M 2007 [2] | Prospective study | Patients with hyperprolactinaemia and healthy volunteers | Serious | Serious | Not serious | Not serious | Very low | This prospective study compared dynamic contrast-enhanced MRI (DCE-MRI) with conventional MRI in patients with hyperprolactinaemia. It reported that DCE-MRI, by demonstrating characteristic delayed and low-peak enhancement patterns due to altered vascular permeability, facilitated the localization of several microadenomas that were indistinct on conventional MRI. The authors concluded that DCE-MRI achieves high sensitivity for detecting lesions ≤5 mm and can effectively help exclude occult microadenomas when no abnormal vascular parameters are seen.  The certainty of the evidence was rated down by two levels:  **Risk of bias:** The study had a small sample size and likely lacked blinding in image interpretation, raising concerns about bias in assessing the diagnostic performance of DCE-MRI.  **Indirectness:** The study population was a selected cohort in a research setting. This limits the direct applicability of the findings to the broader, unselected patient population encountered in routine clinical practice, where the prevalence and characteristics of microadenomas may differ.  Very low certainty evidence suggests that, compared to conventional MRI alone, dynamic contrast-enhanced (DCE)-MRI can improve the detection and anatomical localization of prolactin-secreting microadenomas. However, we **are very uncertain** about this effect, and the decision to use DCE-MRI when feasible should involve shared decision-making, considering factors such as availability and patient preference. |

References:

1. Kreutz J, Vroonen L, Cattin F, et al. Intensity of prolactinoma on T2-weighted magnetic resonance imaging: towards another gender difference[J]. Neuroradiology, 2015, 57 (7): 679-84. doi: 10.1007/s00234-015-1519-3.
2. Manuchehri A M, Sathyapalan T, Lowry M, et al. Effect of dopamine agonists on prolactinomas and normal pituitary assessed by dynamic contrast enhanced magnetic resonance imaging (DCE-MRI)[J]. Pituitary, 2007, 10 (3): 261-6. doi: 10.1007/s11102-007-0048-4.

**Recommendation 7: The frequency of imaging follow-up for treated prolactinomas should be determined after comprehensive assessment of clinical symptoms, biochemical results, previous imaging features, and histopathology (Good Practice Statement). If new symptoms such as visual dysfunction, headache, galactorrhea, or pituitary deficiency occur, increased follow-up frequency is recommended. (Good Practice Statement)**

**Rationale for Good Practice Statement:**

Clarity and feasibility

•Mandatory variables are explicitly listed: clinical status, serum PRL, prior imaging, and histopathology.

•Trigger conditions are unambiguous: treatment resistance or new/worsening visual disturbance, headache, galactorrhoea, or hypopituitarism mandate earlier MRI.

Necessity

•Prevents both over- and under-surveillance: failure to individualise leads to excessive gadolinium exposure or delayed relapse detection.

•Symptom-driven strategy: international consensus already prioritises symptom + PRL monitoring to reduce gadolinium use; without explicit guidance, some centres may default to rigid, calendar-based schedules.

Large, certain net benefit

•Reduced gadolinium retention: limiting scans and preferring macrocyclic agents directly decreases tissue accumulation.

•Early detection of relapse/resistance: prompt MRI triggered by symptoms or rising PRL averts irreversible visual loss.

•Cost-effectiveness: fewer unnecessary scans lower direct costs and patient anxiety.

Prohibitive opportunity cost for direct evidence

•RCT infeasible: individualised intervals dependent on multiple dynamic variables preclude randomisation.

•Observational data consistent: a systematic review would demand enormous resources yet add little incremental certainty.

Coherent evidence chain

•Macroprolactinomas: early imaging at 3-6 months confirms pharmacological tumour shrinkage.

•Microadenomas and post-operative cases: frequency adjusted dynamically by symptoms and PRL balances gadolinium accumulation against early relapse detection.

•MRI triggered by resistance or new neuro-endocrine symptoms reliably identifies tumour regrowth or remnant progression.

**Recommendation 8: For prolactinoma patients with the following risk factors, preoperative CT angiography (CTA) or MR angiography (MRA) is suggested to screen for associated aneurysms: (1) Invasive tumors; (2) Tumor directly contacting the internal carotid artery; (3) Middle-aged and elderly patients (>50 years); (4) Suspicious imaging signs of an aneurysm on head/pituitary MRI; (5) Previous history of transsphenoidal surgery for sellar lesions, diagnosis of refractory pituitary tumor, and/or history of cerebrovascular disease. (Evidence grade: Very Low, Weak Recommendation)**

**8a：For prolactinoma patients with the following risk factors, preoperative CT angiography (CTA) or MR angiography (MRA) is suggested to screen for associated aneurysms: (1) Invasive tumors… (Very low certainty evidence)**

| Study ID | Study Design | Population | Risk of Bias | Indirectness | Inconsistency | Publication Bias | Certainty of Evidence | Findings |
| --- | --- | --- | --- | --- | --- | --- | --- | --- |
| Ogawa Y et al. 2022[1] | Retrospective, single-centre cohort study | 24 pituitary adenomas with concomitant aneurysms among 923 pituitary adenomas | Serious | Serious | Not serious | Not serious | Very low | This retrospective, single-centre cohort study identified 24 pituitary adenomas with concomitant intracranial aneurysms among 923 adenomas. It reported that cavernous-sinus invasion was associated with a higher prevalence of concurrent aneurysms. Among a subgroup of seven patients with invasive tumors, five had undergone aneurysm management prior to 2007, which the authors interpreted as supporting pre-operative aneurysm screening in this high-risk subgroup.  The certainty of the evidence was rated down by two levels:  **Risk of bias:** The retrospective, single-centre design carries a high risk of selection and information bias. The small number of patients with invasive tumors and aneurysms (n=7) further increases imprecision and the potential for chance findings.  **Indirectness:** The study included all types of pituitary adenomas, not exclusively prolactinomas. The association between invasion and aneurysms, as well as the management decisions, were not specifically analysed for the prolactinoma subtype. This introduces uncertainty when applying the findings directly to the recommendation concerning prolactinoma patients.  Very low certainty evidence suggests an association between invasive prolactinomas (e.g., with cavernous-sinus invasion) and a higher prevalence of intracranial aneurysms. We **are very uncertain** about the effect of preoperative CTA or MRA screening on clinical outcomes in these patients. |
| Huang Z et al. 2024[2] | Retrospective, dual-centre cohort study | 29 pituitary adenomas with concomitant aneurysms among 475 pituitary adenomas | Serious | Serious | Not serious | Not serious | Low | This retrospective, dual-centre cohort study of 475 pituitary adenoma patients identified 29 concomitant intracranial aneurysms. The study found that invasive pituitary adenomas were significantly associated with a higher incidence of intracranial aneurysms (OR = 3.26, 95% CI 1.50–7.11; P = 0.003), suggesting that tumor invasion may alter cerebral arterial integrity and hemodynamics, thereby increasing aneurysm risk.  The certainty of the evidence was rated as Low. The initial rating was High due to the large magnitude of effect (OR > 2). It was subsequently downgraded by two levels:  **Risk of bias:** The retrospective study design carries a high risk of selection and information bias.  **Indirectness:** The study population included all pituitary adenoma subtypes with concomitant aneurysms. This provides only indirect evidence for the specific recommendation targeting prolactinoma patients, as the risk profile for aneurysm co-occurrence may differ between tumor types.  Low certainty evidence suggests that in prolactinoma patients with invasive tumors, preoperative screening for associated aneurysms using CT angiography (CTA) or MR angiography (MRA) **may be** considered. |
| Holdaway M et al. 2023[3] | Systematic review + case reports | 24 pituitary adenomas with aneurysms | Serious | Not serious | Not serious | Not serious | Very low | This systematic review noted that cavernous-sinus invasion is associated with a markedly increased risk of concomitant intracranial aneurysm in patients with pituitary adenomas, citing a study of 800 adenomas in which invasion was linked to a higher aneurysm incidence.  The certainty of the evidence was rated down by one level for **risk of bias.** The systematic review incorporated case reports and may have had limitations in the comprehensiveness of its search, study selection, and risk of bias assessment of included studies. The cited primary study (Oh et al.) was not independently assessed for risk of bias within this context.  Very low certainty evidence suggests an association between invasive prolactinomas (e.g., with cavernous-sinus invasion) and a higher prevalence of intracranial aneurysms. We **are very uncertain** about the effect of preoperative CTA or MRA screening on clinical outcomes in these patients. |
| Mercuri V et al. 2022[4] | Systematic review + case reports | 150 pituitary adenomas with intracranial aneurysms | Serious | Not serious | Not serious | Not serious | Very low | This systematic review of 150 pituitary adenomas with intracranial aneurysms explicitly stated that “cavernous-sinus invasion significantly increases the risk of associated aneurysm,” referencing a prior study of 800 adenomas in which invasion was linked to elevated aneurysm rates.  The certainty of the evidence was rated down by one level for **risk of bias**. The systematic review incorporated case reports and may have had limitations in the comprehensiveness of its search, study selection, and risk of bias assessment of included studies, which increases the risk of biased conclusions.  Very low certainty evidence suggests an association between invasive prolactinomas (specifically with cavernous-sinus invasion) and a higher prevalence of intracranial aneurysms. We **are very uncertain** about the effect of preoperative CTA or MRA screening on clinical outcomes in these patients. |
| Piper K J et al. 2021[5] | Systematic review + case reports | 1 macroprolactinoma with cavernous-carotid aneurysm + 20 pituitary adenomas with cavernous aneurysms | Serious | Serious | Not serious | Not serious | Very low | This systematic review included a case of a macroprolactinoma with a cavernous-carotid aneurysm and 20 additional cases of pituitary adenomas with cavernous aneurysms. The review noted that most of the analyzed cases (e.g., from Chuang 2006, Choi 2013) indicated that invasive tumors are more likely to encase or compress the internal carotid artery, a mechanism postulated to increase aneurysm risk.  The certainty of the evidence was rated down by two levels:  **Risk of bias:** The systematic review incorporated case reports and may have had limitations in the comprehensiveness of its search and study selection, which introduces a high risk of selection and reporting bias.  **Indirectness:** The evidence is primarily derived from case reports and a limited series of pituitary adenomas (not exclusively prolactinomas). This provides only indirect evidence for the specific recommendation targeting prolactinoma patients, as the pathological behavior and aneurysm risk may differ across pituitary adenoma subtypes.  Very low certainty evidence suggests an association between invasive prolactinomas (particularly those encasing or compressing the internal carotid artery) and a higher risk of cavernous-carotid aneurysms. We **are very uncertain** about the effect of preoperative CTA or MRA screening on clinical outcomes in these patients. |

**8b: For prolactinoma patients with the following risk factors, preoperative CT angiography (CTA) or MR angiography (MRA) is suggested to screen for associated aneurysms… (2) Tumor directly contacting the internal carotid artery… (Very low certainty evidence).**

| Study ID | Study Design | Population | Risk of Bias | Indirectness | Inconsistency | Publication Bias | Certainty of Evidence | Findings |
| --- | --- | --- | --- | --- | --- | --- | --- | --- |
| Ogawa Y et al. 2022[1] | Retrospective, single-centre cohort study | 24 pituitary adenomas with concomitant aneurysms among 923 pituitary adenomas | Serious | Serious | Not serious | Not serious | Very low | This retrospective, single-centre cohort study of 923 pituitary adenomas identified 24 cases with concomitant intracranial aneurysms. Patients were categorized into an “adherent” group (n=15), where the aneurysm was directly attached to the adenoma, and a “non-adherent” group (n=9). In the adherent group, aneurysms predominantly involved the internal carotid artery (ICA) system. Strong VEGF expression was significantly associated with direct tumor-vessel attachment (p < 0.05), suggesting a potential biological mechanism for aneurysm formation promoted by tumor contact.  The certainty of the evidence was rated down by two levels:  **Risk of bias:** The retrospective, single-centre study design carries a high risk of selection and information bias.  **Indirectness:** The study population included all pituitary adenoma subtypes with concomitant aneurysms. This provides only indirect evidence for the specific recommendation targeting prolactinoma patients, as the propensity for vessel adherence and VEGF expression may differ between tumor types.  Very low certainty evidence suggests an association between prolactinomas that directly contact the internal carotid artery and a higher prevalence of adherent intracranial aneurysms, potentially mediated by factors like VEGF expression. We **are very uncertain** about the effect of preoperative CTA or MRA screening on clinical outcomes in these patients. |
| Huang Z et al. 2024[2] | Retrospective, dual-centre cohort study | 29 pituitary adenomas with concomitant aneurysms among 475 pituitary adenomas | Serious | Serious | Not serious | Not serious | Very low | This retrospective, dual-centre cohort study of 475 pituitary adenomas identified 29 concomitant intracranial aneurysms. The study noted that invasive tumors may affect the internal carotid artery (ICA) system through direct compression and hemodynamic alterations. Among the identified aneurysms, 21 were located along the ICA system, suggesting a close anatomical relationship between tumors and the ICA.  The certainty of the evidence was rated down by two levels:  **Risk of bias:** The retrospective study design carries a high risk of selection and information bias.  **Indirectness:** The study population included all pituitary adenoma subtypes with concomitant aneurysms. This provides only indirect evidence for the specific recommendation targeting prolactinoma patients, as the relationship between tumor proximity, invasion, and aneurysm risk may differ across tumor types.  Very low certainty evidence suggests a possible anatomical relationship between invasive prolactinomas or those near the internal carotid artery (ICA) and aneurysms in the ICA system. We **are very uncertain** about the strength of this association and the effect of preoperative CTA or MRA screening on clinical outcomes in these patients. |
| Holdaway M et al. 2023[3] | Systematic review + case reports | 24 pituitary adenomas with aneurysms | Serious | Not serious | Not serious | Not serious | Very low | This systematic review noted that the spatial relationship between a pituitary adenoma and an aneurysm is a critical determinant of surgical risk. It stated that when a tumor abuts or encases the internal carotid artery (particularly its intrasellar or cavernous segments), the risk of intraoperative aneurysm injury is extremely high. The authors concluded that preoperative CTA or MRA is mandatory to define whether an aneurysm is embedded in or merely adjacent to the tumor.  The certainty of the evidence was rated down by one level for **risk of bias**. The systematic review incorporated case reports and may have had limitations in the comprehensiveness of its search, study selection, and risk of bias assessment of included studies, which increases the risk of biased or overestimated conclusions regarding risk.  Very low certainty evidence addresses the potential surgical risk in patients where a prolactinoma is in direct contact with or encases the internal carotid artery. We **are very uncertain** about the effect of preoperative CTA or MRA screening on clinical outcomes in these patients. |
| Mercuri V et al. 2022[4] | Systematic review + case reports | 150 pituitary adenomas with intracranial aneurysms | Serious | Not serious | Not serious | Not serious | Very low | This systematic review of 150 pituitary adenomas with intracranial aneurysms emphasised that the spatial relationship between the adenoma and the aneurysm is a key factor in surgical risk. The authors stated that if the tumor is close to or surrounds the internal carotid artery (ICA), particularly its cavernous portion, the risk of intra-operative rupture is substantial. They concluded that preoperative CTA, MRA, or DSA should be performed to delineate the aneurysm-tumor interface and prevent intra-operative catastrophe.  The certainty of the evidence was rated down by one level for **risk of bias**. The systematic review incorporated case reports and may have had limitations in the comprehensiveness of its search, study selection, and risk of bias assessment of included studies, which increases the risk of biased conclusions regarding the magnitude of risk and the necessity of imaging.  Very low certainty evidence addresses the potential surgical risk in patients where a prolactinoma is close to or surrounds the internal carotid artery (ICA). We **are very uncertain** about the actual magnitude of this risk and about the effect of preoperative CTA or MRA screening on clinical outcomes in these patients, due to serious limitations in the evidence base. |
| Piper K J et al. 2021[5] | Systematic review + case reports | 1 macroprolactinoma with cavernous-carotid aneurysm + 20 pituitary adenomas with cavernous aneurysms | Serious | Not serious | Not serious | Not serious | Very low | This systematic review included a case of a macroprolactinoma with a cavernous-carotid aneurysm and 20 additional cases of pituitary adenomas with cavernous aneurysms. In all 20 reviewed cases, direct tumor contact with the internal carotid artery (ICA) was described (e.g., “adenoma encasing the ICA” “tumour adherent to the ICA”). The review highlighted radiographic blurring of the tumor-arterial interface as an unequivocal warning sign.  The certainty of the evidence was rated down by one level for **risk of bias.** The systematic review incorporated case reports, which are inherently prone to publication and selection bias. The consistent finding across these highly selected cases may not reflect the true prevalence or risk in the broader population of prolactinoma patients.  Very low certainty evidence describes a consistent finding of direct contact between the tumor and the internal carotid artery (ICA) in a series of reported cases involving pituitary adenomas and cavernous aneurysms. We **are very uncertain** about the generalizability of this finding and about the effect of preoperative CTA or MRA screening on clinical outcomes in patients with this anatomical relationship, due to serious limitations in the evidence base. |

**8c: For prolactinoma patients with the following risk factors, preoperative CT angiography (CTA) or MR angiography (MRA) is suggested to screen for associated aneurysms: ... (3) Middle-aged and elderly patients (>50 years) ... (Very low certainty evidence)**

| Study ID | Study Design | Population | Risk of Bias | Indirectness | Inconsistency | Publication Bias | Certainty of Evidence | Findings |
| --- | --- | --- | --- | --- | --- | --- | --- | --- |
| Ogawa Y et al. 2022[1] | Retrospective, single-centre cohor tstudy | 24 pituitary adenomas with concomitant aneurysms among 923 pituitary adenomas | Serious | Serious | Not serious | Not serious | Very low | This retrospective, single-centre cohort study of 923 pituitary adenomas identified 24 cases with concomitant intracranial aneurysms. The mean age of patients with aneurysms in this cohort was 56.8 years. The authors cited empirical evidence indicating that advancing age is associated with an increased prevalence of intracranial aneurysms, suggesting this cohort reflects a heightened risk profile among older patients.  The certainty of the evidence was rated down by two levels:  **Risk of bias:** The retrospective, single-centre study design carries a high risk of selection and information bias.  **Indirectness:** The study population included all pituitary adenoma subtypes with concomitant aneurysms and reported only the mean age of the aneurysm subgroup. This provides only indirect evidence for the specific recommendation targeting prolactinoma patients aged >50 years, as it does not directly analyze age as an independent risk factor within prolactinomas, nor does it compare risk across age groups.  Very low certainty evidence describes an older mean age (56.8 years) in a cohort of patients with pituitary adenomas and concomitant intracranial aneurysms. We **are very uncertain** about whether age (>50 years) is a specific risk factor for aneurysms in prolactinoma patients, or about the effect of preoperative CTA or MRA screening in this age group, due to serious limitations in the evidence. |
| Huang Z et al. 2024[2] | Retrospective, dual-centre cohort study | 29 pituitary adenomas with concomitant aneurysms among 475 pituitary adenomas | Serious | Serious | Not serious | Not serious | Low | This retrospective, dual-centre cohort study of 475 pituitary adenoma patients, which identified 29 concomitant intracranial aneurysms, performed a multivariable analysis. It found that age >50 years was an independent risk factor for intracranial aneurysm in patients with pituitary adenoma (OR=2.69; 95%CI: 1.20-6.04; P=0.016).  The certainty of the evidence is rated as **Low**. The initial rating was **High** due to the large magnitude of effect (OR > 2). It was subsequently downgraded by two levels:  **Risk of bias:** The retrospective study design carries a high risk of selection and information bias.  **Indirectness:** The study population included all pituitary adenoma subtypes. This provides indirect evidence for the specific recommendation targeting prolactinoma patients, as the strength of the association between age and aneurysm risk may differ across tumor types.  Low certainty evidence suggests that in patients with pituitary adenoma, age >50 years is associated with an increased risk of intracranial aneurysm. Based on this, preoperative screening for associated aneurysms using CTA or MRA **may be** considered in prolactinoma patients aged >50 years. |
| Holdaway M et al. 2023[3] | Systematic review + case reports | 24 pituitary adenomas with aneurysms | Serious | Serious | Not serious | Not serious | Very low | This systematic review noted that published series of patients with concomitant pituitary adenomas and intracranial aneurysms report a mean age of 54.2 years, and stated that middle-aged and elderly individuals constitute a population of interest in this context. The authors commented that while these data do not directly establish age as a screening criterion, they provide indirect support when considered alongside general epidemiological findings that aneurysmal risk increases with age.  The certainty of the evidence was rated down by two levels:  **Risk of bias:** The systematic review incorporated case reports and may have had limitations in its methodology, introducing a high risk of selection and reporting bias.  **Indirectness:** The evidence relies on descriptive mean age data from mixed pituitary adenoma series and on extrapolation from general epidemiology, providing only indirect support for age as a specific risk factor in prolactinoma patients.  Very low certainty evidence describes a reported mean age of 54.2 years in published series of patients with pituitary adenomas and concomitant aneurysms. We **are very uncertain** about whether age (>50 years) is a specific, independent risk factor for aneurysms in prolactinoma patients, or about the effect of preoperative CTA or MRA screening in this age group, due to serious limitations in the evidence. |
| Mercuri V et al. 2022[4] | Systematic review + case reports | 150 pituitary adenomas with intracranial aneurysms | Serious | Not serious | Not serious | Not serious | Very low | This systematic review of 150 pituitary adenomas with intracranial aneurysms reported a mean patient age of 52.15 years in its cohort. The authors stated that this finding underscores middle-aged and elderly patients as a high-prevalence group for the co-occurrence of aneurysm and pituitary adenoma, while noting that this age threshold was not explicitly validated as an indication for screening.  The certainty of the evidence was rated down by one level for **risk of bias**. The systematic review incorporated case reports and may have had limitations in the comprehensiveness of its search, study selection, and risk of bias assessment of included studies, which increases the risk of biased conclusions.  Very low certainty evidence describes a reported mean age of 52.15 years in a cohort of patients with pituitary adenomas and concomitant intracranial aneurysms. We **are very uncertain** about whether age (>50 years) is a specific risk factor for aneurysms in prolactinoma patients, or about the effect of preoperative CTA or MRA screening in this age group, due to serious limitations in the evidence. |
| Piper K J et al. 2021[5] | Systematic review + case reports | 1 macroprolactinoma with cavernous-carotid aneurysm + 20 pituitary adenomas with cavernous aneurysms | Serious | Serious | Not serious | Not serious | Very low | This systematic review noted that the majority of reported cases of pituitary adenomas with cavernous aneurysms fall within the 50- to 70-year age range (e.g., 58 years in Yamada et al., 2012; 55 years in Peng et al., 2015). The review stated that middle-aged and elderly patients are an independent risk factor for aneurysm formation, and this risk is amplified when pituitary adenoma is present.  The certainty of the evidence was rated down by two levels:  **Risk of bias:** The systematic review incorporated case reports, which are prone to publication and selection bias.  **Indirectness:** The evidence is based on selected case reports of mixed pituitary adenomas, providing only indirect support for age as a specific risk factor in prolactinoma patients.  Very low certainty evidence suggests that reported cases of pituitary adenomas with cavernous aneurysms often occur in patients aged 50-70 years. We **are very uncertain** about whether age (>50 years) is a specific risk factor for aneurysms in prolactinoma patients, or about the effect of preoperative CTA or MRA screening in this age group. |

**8d: For prolactinoma patients with the following risk factors, preoperative CT angiography (CTA) or MR angiography (MRA) is suggested to screen for associated aneurysms: ... (4) Suspicious imaging signs of an aneurysm on head/pituitary MRI … (Very low certainty evidence).**

| Study ID | Study Design | Population | Risk of Bias | Indirectness | Inconsistency | Publication Bias | Certainty of Evidence | Findings |
| --- | --- | --- | --- | --- | --- | --- | --- | --- |
| Ogawa Y et al. 2022[1] | Retrospective, single-centre cohort study | 24 pituitary adenomas with concomitant aneurysms among 923 pituitary adenomas | Serious | Serious | Not serious | Not serious | Very low | This retrospective, single-centre cohort study of 923 pituitary adenomas, which identified 24 concomitant intracranial aneurysms, reported that all participants underwent preoperative MRI and time-of-flight MRA. The detection of imaging red flags was described as pivotal for identifying co-existing aneurysms. The authors noted that while the dataset does not formally quantify “suspicious imaging features” as an independent risk factor, the universal reliance on cross-sectional imaging underpins its importance, though interpretation should consider additional clinical variables.  The certainty of the evidence was rated down by two levels:  **Risk of bias:** The retrospective, single-centre study design carries a high risk of selection and information bias.  **Indirectness:** The study population included all pituitary adenoma subtypes and did not formally analyze “suspicious imaging signs” as a risk factor. This provides only indirect evidence for the specific recommendation regarding the interpretation of such signs in prolactinoma patients.  Very low certainty evidence indicates that in a cohort of patients with pituitary adenomas, cross-sectional imaging (MRI/MRA) was used to identify co-existing intracranial aneurysms. We **are very uncertain** about the diagnostic accuracy of specific “suspicious imaging signs” for predicting aneurysms in prolactinoma patients, or about the effect of targeted screening based on such signs, due to serious limitations in the evidence. |
| Huang Z et al. 2024[2] | Retrospective, dual-centre cohort study | 29 pituitary adenomas with concomitant aneurysms among 475 pituitary adenomas | Serious | Serious | Not serious | Not serious | Very low | This retrospective, dual-centre cohort study of 475 pituitary adenomas, which identified 29 concomitant intracranial aneurysms, explicitly advocated for routine preoperative CTA screening and recognized MRI/CTA as the principal modalities for aneurysm detection. The authors noted that, although multivariable statistics incorporating “MRI suspicious findings” were absent, the premise that imaging abnormalities drive case finding provides indirect corroboration for their importance.  The certainty of the evidence was rated down by two levels:  **Risk of bias:** The retrospective study design carries a high risk of selection and information bias.  **Indirectness:** The study population included all pituitary adenoma subtypes and did not formally analyze "suspicious MRI findings" as a risk factor. This provides only indirect evidence for the specific recommendation regarding the interpretation of such signs in prolactinoma patients.  Very low certainty evidence indicates that in a cohort of patients with pituitary adenomas, MRI and CTA were recognized as key modalities for detecting co-existing intracranial aneurysms. We **are very uncertain** about the diagnostic accuracy of specific "suspicious imaging signs" on MRI for predicting aneurysms in prolactinoma patients, or about the effect of targeted screening based on such signs, due to serious limitations in the evidence. |
| Holdaway M et al. 2023[3] | Systematic review + case reports | 24 pituitary adenomas with aneurysms | Serious | Not serious | Not serious | Not serious | Very low | This systematic review reported that CTA with three-dimensional reconstructions is considered mandatory to delineate the anatomical relationship between an aneurysm and an adenoma. The authors suggested that when MRI reveals equivocal findings (e.g., flow voids or atypical enhancement), subsequent CTA or MRA is obligatory to secure the diagnosis and prevent missed lesions. This diagnostic algorithm was illustrated by index cases in which MRI-detected aneurysms were subsequently verified by CTA.  The certainty of the evidence was rated down by one level due to serious **risk of bias**: The evidence is primarily derived from a systematic review of case reports and expert opinion. This study design inherently carries a very high risk of selection and reporting bias, as it focuses on published, often remarkable cases, and does not represent an unselected patient population.  Very low certainty evidence indicates that in a selected group of patients with pituitary adenomas and aneurysms, expert opinion emphasizes the use of CTA/MRA following inconclusive MRI findings to confirm a diagnosis. We **are very uncertain** about the diagnostic accuracy of specific “suspicious imaging signs” on MRI for predicting co-existing aneurysms in prolactinoma patients, or about the clinical impact (e.g., on patient outcomes) of a systematic screening algorithm triggered by such MRI findings in this specific population. |
| Mercuri V et al. 2022[4] | Systematic review + case reports | 150 pituitary adenomas with intracranial aneurysms | Serious | Not serious | Not serious | Not serious | Very low | This systematic review reported a recommendation that any MRI revealing non-characteristic enhancement or flow-void signals should prompt confirmatory digital subtraction angiography (DSA) or CTA to exclude an aneurysm. The review documented illustrative cases in which initial MRI-based detection was followed by DSA confirmation, a process described as preventing diagnostic oversight.  The certainty of the evidence was rated down by one level due to serious **risk of bias:** The evidence is derived from a systematic review of case reports. This study design is inherently subject to a very high risk of selection, reporting, and publication bias, as it synthesizes data from published individual cases that are not representative of an unselected clinical population.  Very low certainty evidence indicates that in a selected cohort of patients with pituitary adenomas and aneurysms, expert opinion recommends confirmatory vascular imaging (DSA/CTA) following the detection of non-specific signals on MRI. We **are very uncertain** about the predictive value of specific “suspicious imaging signs” on routine MRI for the presence of an aneurysm in prolactinoma patients, or about the effect of implementing this confirmatory testing strategy on diagnostic accuracy or patient-important outcomes in the target population. |
| Piper K J et al. 2021[5] | Systematic review + case reports | 1 macroprolactinoma with cavernous-carotid aneurysm + 20 pituitary adenomas with cavernous aneurysms | Serious | Not serious | Not serious | Not serious | Very low | This systematic review reported that 18 of the 20 aneurysms were incidental imaging discoveries. The review described characteristic MRI signatures associated with these aneurysms, including aberrant flow voids on T1-weighted images and time-of-flight MRA, and focal intratumoral hypointensity suggestive of aneurysmal wall calcification or intraluminal flow.  The certainty of the evidence was rated down by one level due to serious **Risk of Bias**: The evidence is derived from a systematic review of case reports. This study design carries an extremely high risk of selection and reporting bias, as it synthesizes published cases that are inherently non-representative of a clinical population.  Very low certainty evidence suggests that in a small, selected series of pituitary adenoma cases with coexisting cavernous aneurysms, most aneurysms were incidental findings. The evidence describes MRI features (e.g., aberrant flow voids, focal hypointensity) observed in these cases. We **are very uncertain** about the diagnostic accuracy of these “suspicious imaging signs” for predicting an aneurysm in a broader population of prolactinoma patients. |

**8e: For prolactinoma patients with the following risk factors, preoperative CT angiography (CTA) or MR angiography (MRA) is suggested to screen for associated aneurysms: ... (5) Previous history of transsphenoidal surgery for sellar lesions, diagnosis of refractory pituitary tumor, and/or history of cerebrovascular disease... (Very low certainty evidence).**

| Study ID | Study Design | Population | Risk of Bias | Indirectness | Inconsistency | Publication Bias | Certainty of Evidence | Findings |
| --- | --- | --- | --- | --- | --- | --- | --- | --- |
| Holdaway M et al. 2023[3] | Systematic review + case reports | 24 pituitary adenomas with aneurysms | Serious | Serious | Not serious | Not serious | Very low | This systematic review of case reports provided indirect evidence. None of the included primary studies explicitly examined a prior history of transsphenoidal surgery or a diagnosis of refractory pituitary tumor as specific risk factors for intracranial aneurysm formation. The review notes, however, that the literature consistently suggests prior operative manipulation can alternative anatomy, potentially increasing the susceptibility of peritumoral vessels (including pre-existing aneurysms) to intraoperative injury.  The certainty of the evidence was rated down by two levels:  **Risk of Bias:** The evidence is derived from a systematic review of case reports. This study design carries an extremely high risk of selection and reporting bias, as it synthesizes published, non-representative cases.  **Indirectness:** There is a critical lack of direct evidence. The review did not identify any study that directly investigated the association between the specific risk factors in the recommendation (prior transsphenoidal surgery, refractory tumor) and the presence of intracranial aneurysms in the target population (prolactinoma patients).  Very low certainty evidence indicates that, in a selected series of pituitary adenoma cases with aneurysms, there is a pathophysiological rationale for the possibility that prior surgery alters vascular anatomy. We **are very uncertain** about the actual risk of having an associated intracranial aneurysm specifically in prolactinoma patients with a history of transsphenoidal surgery or refractory disease, or about the effect of screening in this subgroup, due to serious limitations in the direct evidence. |
| Mercuri V et al. 2022[4] | Systematic review + case reports | 150 pituitary adenomas with intracranial aneurysms | Serious | Serious | Not serious | Not serious | Very low | This systematic review of case reports noted that a documented history of cerebrovascular disease (e.g., treated or residual aneurysms) is itself considered a high-risk condition, mandating heightened vigilance for de novo or persistent aneurysms. The authors suggested that, collectively, these considerations provide indirect support for including prior surgical history in screening algorithms.  The certainty of the evidence was rated down by two levels:  **Risk of Bias**: The evidence is derived from a systematic review of case reports. This study design carries an extremely high risk of selection and reporting bias, as it synthesizes published, non-representative cases.  **Indirectness**: The evidence is not directly applicable to the specific recommendation. The review discusses the general principle of “history of cerebrovascular disease” as a risk factor, but provides no direct data linking the specific risk factors in the recommendation to the presence of aneurysms in the target population.  Very low certainty evidence indicates that, in a selected series of pituitary adenoma cases with aneurysms, a history of cerebrovascular disease is considered a high-risk condition warranting vigilance. We **are very uncertain** about the strength of association between a history of cerebrovascular disease or prior surgery and the presence of an intracranial aneurysm specifically in prolactinoma patients, or about the effect of preoperative screening on outcomes in this subgroup, due to serious limitations in the evidence. |
| Piper K J et al. 2021[5] | Systematic review + case reports | 1 macroprolactinoma with cavernous-carotid aneurysm + 20 pituitary adenomas with cavernous aneurysms | Serious | Serious | Not serious | Not serious | Very low | This systematic review of case reports noted that published series suggest previous transsphenoidal or transcranial procedures could modify the parasellar micro-architecture and precipitate arterial wall trauma or chronic inflammation, which are recognized as potential catalysts for aneurysmal degeneration. The review also catalogued cases of recurrent or medically refractory pituitary tumours (where prior surgery had failed) in which aneurysm-adenoma adhesions were exacerbated by earlier interventions, which the authors suggested further substantiates the rationale for targeted screening in patients with a surgical history.  The certainty of the evidence was rated down by two levels:  **Risk of Bias:** The evidence is derived from a systematic review of case reports. This study design carries an extremely high risk of selection and reporting bias, as it synthesizes published, non-representative cases. The conclusions are based on descriptive cases and theoretical rationale rather than direct analytical data.  **Indirectness:** The evidence does not directly address the risk factor-outcome relationship in question. It provides no direct data on the association between prior transsphenoidal surgery/refractory disease and aneurysm presence in prolactinoma patients, relying instead on theoretical rationale and case descriptions.  Very low certainty evidence indicates that, in selected published cases of pituitary adenomas with aneurysms, a theoretical rationale exists suggesting a link between prior surgery and vascular changes. We **are very uncertain** about the actual prevalence of intracranial aneurysms in prolactinoma patients with a history of transsphenoidal surgery or refractory disease, and about the clinical utility of screening based on this history. |

References:

1. Ogawa Y, Watanabe M, Tominaga T. Pituitary Adenomas Associated with Intracranial Aneurysms: the Clinical Characteristics, Therapeutic Strategies, and Possible Effects of Vascular Remodeling Factors[J]. J Neurol Surg A Cent Eur Neurosurg, 2022, 83 (6): 555-60. doi: 10.1055/s-0041-1739232.
2. Huang Z, Yang Z, Xu L, et al. Clinical characteristics and treatment strategies for pituitary adenoma associated with intracranial aneurysm[J]. Chin Neurosurg J, 2024, 10 (1): 18. doi: 10.1186/s41016-024-00370-7.
3. Holdaway M, Huda S, D'Amico R S, et al. An algorithm for the treatment of concurrent pituitary adenoma and cavernous sinus aneurysm: A systematic review & case report[J]. J Clin Neurosci, 2023, 117 46-53. doi: 10.1016/j.jocn.2023.09.012.
4. Mercuri V, Armocida D, Paglia F, et al. Giant Prolactinoma Embedded by Pseudoaneurysm of the Cavernous Carotid Artery Treated with a Tailored Therapeutic Scheme[J]. J Neurosci Rural Pract, 2022, 13 (3): 358-69. doi: 10.1055/s-0042-1749662.
5. Piper K J, Karsy M, Barton B, et al. Management of Coincident Pituitary Macroadenoma and Cavernous Carotid Aneurysm: A Systematic Literature Review[J]. J Neurol Surg Rep, 2021, 82 (3): e25-e31. doi: 10.1055/s-0041-1735904.

# **Part 3 Treatment**

**3.1 Pharmacological therapy**

**Recommendation 9: Dopamine receptor agonist therapy is effective in reducing serum PRL levels, improving clinical manifestations associated with hyperprolactinemia, and shrinking the size of pituitary prolactinomas. (Evidence grade: Moderate, Strong Recommendation)**

| Study ID | Study Design | Population | Risk of Bias | Indirectness | Inconsistency | Publication Bias | Certainty of Evidence | Findings |
| --- | --- | --- | --- | --- | --- | --- | --- | --- |
| Wang AT et al., 2012[1] | Systematic review and meta-analysis | >3 000 patients with hyperprolactinaemia, including micro- and macro-prolactinomas | Not serious | Not serious | Not serious | Not serious | Low | This systematic review and meta-analysis included over 3,000 patients with hyperprolactinaemia (including micro- and macro-prolactinomas). It reported that dopamine agonist therapy was consistently superior to no treatment. A meta-analysis of three observational studies and one RCT showed dopamine agonists produced a clinically and statistically significant reduction in serum prolactin (weighted mean difference −45 ng/mL; 95% CI −77 to −11) and a significant decrease in persistent hyperprolactinaemia (relative risk 0.90; 95% CI 0.81–0.99). A synthesis of over 150 non-comparative cohort studies reported normalization of prolactin in 68% of patients, tumour shrinkage in 62%, resolution of galactorrhoea in 86%, resumption of menses in 78%, and improvement in sexual function in 67%. Pooled data from six observational studies and three RCTs demonstrated that cabergoline was superior to bromocriptine for sustained normoprolactinaemia, restoration of regular menses/cessation of oligo-menorrhoea, and resolution of galactorrhoea, with a lower incidence and severity of adverse effects (e.g., nausea, hypotension).  This initial rating reflects that the body of evidence is primarily based on observational data and a limited number of randomized trials. No further upgrading or downgrading was applied for this specific comparison.  Low certainty evidence suggests that dopamine receptor agonist therapy **may reduce** serum prolactin levels, increase the rates of prolactin normalization, tumour shrinkage, and resolution of clinical symptoms (e.g., galactorrhoea, menstrual irregularities), and improve sexual function in patients with hyperprolactinaemia and prolactinomas when compared to no treatment. Furthermore, cabergoline **may lead to** better efficacy and fewer adverse effects than bromocriptine. |
| Colao 2000[2] | Prospective cohort study | 110 patients with giant prolactinomas | Not serious | Serious | Not serious | Not serious | Very low | This prospective cohort study of 110 patients with giant prolactinomas reported that among dopamine-agonist-naïve patients, cabergoline yielded robust responses: tumour volume reduction of ≥80% in 92.3% of patients, complete radiological disappearance of the tumour in 61.5%, prolactin normalization in 80.8% within 1-6 months, and a mean volumetric shrinkage of 92.1%. The authors suggested that cabergoline should be considered first-line therapy for giant prolactinomas, as its efficacy in treatment-naïve patients markedly exceeded that observed in patients previously exposed to other dopamine agonists (e.g., bromocriptine or quinagolide).  The certainty of the evidence was rated down by one level due to serious indirectness:  **Indirectness:** The study population is highly specific and differs critically from the broader target population of the recommendation. The evidence is indirect regarding the population as it exclusively involves patients with giant prolactinomas, while the recommendation applies to all pituitary prolactinomas (including micro- and macroprolactinomas).  Very low certainty evidence indicates that in treatment-naïve patients with giant prolactinomas, cabergoline was associated with high rates of tumour shrinkage and prolactin normalization. We **are very uncertain** about the effect of dopamine receptor agonist therapy on patient-important outcomes (e.g., symptom resolution) or its comparative effectiveness relative to other drugs for the broader population of prolactinoma patients, including those with non-giant tumours or prior treatment exposure. |
| Di Sarno 2001[3] | Retrospective cohort study | 207 treatment-naïve patients with hyperprolactinaemia (de-novo diagnosis) | Not serious | Not serious | Not serious | Not serious | Low | This retrospective cohort study of 207 treatment-naïve patients with hyperprolactinaemia compared the efficacy and safety of cabergoline versus bromocriptine as first-line therapy. The study reported significantly higher rates of prolactin normalization with cabergoline across all subgroups: giant prolactinomas (82.1% vs. 46.4%), micro-prolactinomas (90.0% vs. 56.8%), and non-tumoural hyperprolactinaemia (100% vs. 80%). Median tumour volume reduction was also significantly greater with cabergoline for both giant adenomas (62.2% vs. 22.2%) and micro-adenomas (58.9% vs. 4.3%). Rates of visual field recovery normalization were 89.5% with cabergoline versus 73.3% with bromocriptine. The adverse-event rate was lower with cabergoline (3.3% vs. 21.8%), while discontinuation due to side effects was 0% in both groups.  The overall certainty of the evidence for this comparison (cabergoline vs. bromocriptine) was rated as low. This initial rating reflects that the evidence is derived from a single, non-randomized observational study (retrospective cohort design). No further upgrading or downgrading was applied based on the provided information.  Low certainty evidence suggests that in treatment-naïve patients with hyperprolactinaemia or prolactinomas, cabergoline **may be** more effective than bromocriptine in normalizing serum prolactin levels, reducing tumour volume, and improving visual field deficits, and may be associated with fewer adverse effects. |
| Corsello 2003[4] | Prospective cohort study | 10 male patients with giant prolactinomas | Not serious | Serious | Not serious | Not serious | Very low | This prospective cohort study of 10 male patients with giant prolactinomas reported that treatment led to a mean reduction in serum prolactin of ≥96% (from 5,794±1,996μg/L to 77±38μg/L), with normalization achieved in 50% (5/10) of patients. Regarding tumour response, 90% (9/10) of patients exhibited shrinkage after 6-12 months: >95% reduction in 3 patients, >50% in 4, and >25% in 2; one patient had no volume change despite prolactin normalization. Of the 7 patients with baseline visual field defects, 6 improved and 2 achieved complete normalization. Among 8 patients with baseline sexual dysfunction, complete restoration of function occurred in 2, and fertility (partner pregnancy) was restored in 1. Treatment was well-tolerated with no discontinuations due to adverse events.  The certainty of the evidence was rated down by one level due to serious indirectness:  **Indirectness**: The study population is narrowly defined and substantially differs from the target population of the recommendation. The evidence is indirect regarding the population on two key dimensions: 1) it includes only patients with giant prolactinomas, a rare and severe subtype, and 2) it includes only male patients.  Very low certainty evidence from a small, single-arm study indicates that in a highly specific group of male patients with giant prolactinomas, dopamine agonist therapy was associated with large reductions in serum prolactin, frequent tumour shrinkage, and improvements in visual and sexual function. We **are very uncertain** about the effect of this therapy on the outcomes specified in the recommendation for the broader population of prolactinoma patients, including females and those with non-giant tumours. |
| Colao 2003-1[5] | Prospective cohort study | 219 newly diagnosed patients with hyperprolactinaemia | Not serious | Not serious | Not serious | Not serious | Low | This prospective cohort study of 219 newly diagnosed patients with hyperprolactinaemia evaluated cabergoline treatment for 6 months. It reported large reductions in serum prolactin levels for both micro-adenomas (89.2% to 92.6%) and macro-adenomas (93.4% to 96.4%). Prolactin normalization rates were 86% for micro-adenomas and 64% for macro-adenomas. Median tumour shrinkage was 45-52% for macro-adenomas and 38-44% for micro-adenomas, with complete radiological disappearance observed in 9 macro- and 14 micro-adenomas. Clinical improvements included resumption of regular menses in 82% of women, improved libido/erectile function in 57% of men, and resolution of visual field defects in 61% of women and 71% of men. Mild adverse events occurred in six patients with no treatment discontinuations.  The overall certainty of the evidence for the effect of cabergoline therapy was rated as low. This initial rating reflects that the evidence is derived from a single, non-randomized observational study (prospective cohort design) without a concurrent control group. No further upgrading or downgrading was applied based on the provided information.  **Low certainty evidence** suggests that in newly diagnosed patients with prolactinomas, cabergoline treatment **may significantly reduce** serum prolactin levels, normalize prolactin in a majority of patients, shrink tumour volume, and improve associated clinical symptoms such as menstrual irregularities, sexual dysfunction, and visual field deficits. |
| Colao 2003-2[6] | Prospective cohort study | 297 treatment-naïve patients with hyperprolactinaemia who achieved cabergoline-withdrawal criteria | Not serious | Not serious | Not serious | Not serious | Low | This prospective cohort study of 297 treatment-naïve patients with hyperprolactinaemia who met criteria for cabergoline withdrawal reported an overall prolactin normalization rate of 92% (273/297). Tumour volume reduction of ≥50% (meeting withdrawal criteria) was achieved in 67% (200/297) of patients, with near-complete disappearance observed in 30% of macro-prolactinomas. No serious adverse events were reported; only six patients experienced mild nausea or transient hypotension, and no treatment discontinuations occurred due to adverse effects. Following drug cessation, the study reported no tumour regrowth or prolactin rebound, suggesting durable disease control.  The overall certainty of the evidence for the efficacy and safety of cabergoline in this context was rated as **low**. This initial rating reflects that the evidence is derived from a single, non-randomized observational study without a concurrent control group. No further upgrading or downgrading was applied based on the provided information.  Low certainty evidence suggests that in treatment-naïve patients with hyperprolactinaemia or prolactinomas who achieve a response, cabergoline therapy **may lead to** high rates of prolactin normalization and substantial tumour shrinkage, and may be well-tolerated. The evidence also suggests that drug withdrawal after successful treatment may be feasible without immediate recurrence in a proportion of patients. |
| Colao 2004[7] | Prospective cohort study | 51 treatment-naïve male patients with prolactinomas | Not serious | Not serious | Not serious | Not serious | Low | This prospective cohort study of 51 treatment-naïve male patients with prolactinomas reported that after 24 months of cabergoline therapy, the overall prolactin normalization rate was 76.5%, the mean tumour volume reduction exceeded 70%, and tumours disappeared completely in approximately one-third of patients.  The overall certainty of the evidence for the efficacy of cabergoline in this population was rated as **low**. This initial rating reflects that the evidence is derived from a single, non-randomized observational study without a concurrent control group. No further upgrading or downgrading was applied based on the provided information.  **Low certainty evidence** suggests that in treatment-naïve male patients with prolactinomas, cabergoline therapy over 24 months **may lead to** prolactin normalization in a majority of patients and **may be** associated with substantial tumour shrinkage, including complete radiological disappearance in some. |
| De Rosa M 2006[8] | Prospective cohort study | 43 treatment-naïve male patients with macro- or micro-prolactinomas versus healthy controls | Not serious | Serious | Not serious | Not serious | Very low | This prospective cohort study reported that short-term (6 months) cabergoline therapy markedly reduced prolactin levels, with 76-80% of patients achieving normoprolactinaemia. Semen quality improved significantly, although most parameters remained below control values. Long-term (24 months) treatment resulted in restored gonadal function in 66.7% of patients without the need for hormonal replacement.  The certainty of the evidence was rated down by one level due to serious **indirectness**: The study population does not fully match the target population of the recommendation. The evidence is indirect regarding the population, as it included only male patients. The effect of therapy on outcomes such as gonadal and sexual function didn’t directly generalizable to the broader population addressed by the recommendation, which includes female patients.  **Very low certainty evidence** indicates that in treatment-naïve male patients with prolactinomas, cabergoline therapy was associated with reductions in prolactin levels, improvements in semen quality, and restoration of gonadal function over time. We **are very uncertain** about the effect of dopamine receptor agonist therapy on all patient-important outcomes specified in the recommendation (including tumour shrinkage and symptom resolution in female patients) for the broader population of prolactinoma patients. |
| Shimon 2007[9] | Retrospective case series | male patients with giant invasive prolactinomas who had received prior therapy | Serious | Serious | Not serious | Not serious | Very low | This retrospective case series of male patients with giant invasive prolactinomas who had received prior therapy reported that first-line cabergoline demonstrated high efficacy and favourable safety. The study observed prolactin normalization in 83% of patients, tumour shrinkage in 91%, and marked visual improvement. No serious adverse events occurred, even with prolonged high-dose regimens (up to 7 mg per week).  The certainty of the evidence was rated down by two levels:  **Risk of Bias:** The evidence is derived from a retrospective case series. This study design carries a very high risk of selection, reporting, and recall bias, as it reviews past medical records of a non-consecutive or selected series of patients without a control group.  **Indirectness:** The study population is highly specific and differs substantially from the target population of the recommendation. The evidence is indirect regarding the population on multiple dimensions: it includes only patients with giant invasive prolactinomas, only male patients, and only those who had received prior therapy.  **Very low certainty evidence** indicates that in a highly specific, pre-treated population of male patients with giant invasive prolactinomas, cabergoline therapy was associated with high rates of prolactin normalization, tumour shrinkage, and visual improvement. We **are very uncertain** about the effect of dopamine agonist therapy on the outcomes specified in the recommendation for the broader, treatment-naïve population of prolactinoma patients, which includes females and those with non-invasive or smaller tumours. |
| Ono 2008[10] | Prospective cohort study | patients with prolactinomas, both treatment-naïve and previously treated | Not serious | Serious | Not serious | Not serious | Very low | This prospective cohort study of patients with prolactinomas (both treatment-naïve and previously treated) reported that cabergoline as first-line therapy achieved prolactin (PRL) normalization in 95% of untreated patients within six months. Among patients resistant to or intolerant of prior agents, PRL normalization was ultimately attained in 96-99%. No serious adverse events were observed, and tolerability was reported to be excellent.  The certainty of the evidence was rated down by one level due to **serious indirectness**: The study population is not fully described and not precisely match the target population of the recommendation. The evidence is indirect regarding the outcomes as it primarily reports on prolactin normalization but provides limited or no direct data on other critical outcomes mentioned in the recommendation. The applicability of the findings to all prolactinoma subtypes is unclear.  Very low certainty evidence from a cohort study indicates that in a mixed population of patients with prolactinomas, cabergoline therapy was associated with high rates of serum prolactin normalization, both in treatment-naïve patients and in those previously treated with other agents. We **are very uncertain** about the effect of this therapy on other patient-important outcomes specified in the recommendation, such as tumour shrinkage and the resolution of specific clinical manifestations, and about its comparative effectiveness or applicability to the full spectrum of prolactinoma patients. |
| Vallette 2009[11] | Prospective case–control study | prolactinoma patients receiving cabergoline vs healthy controls | Not serious | Serious | Not serious | Not serious | Very low | This prospective case-control study reported that this cabergoline regimen did not increase the risk of cardiac valvulopathy, achieved prolactin (PRL) normalization in over 90% of patients, and was well-tolerated with no treatment discontinuations attributable to valvular disease.  The certainty of the evidence was rated down by one level due to **serious indirectness**: The evidence is indirect regarding the **outcomes.** While the study provides direct evidence on the safety outcome of valvulopathy and the surrogate efficacy outcome of prolactin normalization, it does not address the two other patient-important outcomes that are central to the recommendation.  Very low certainty evidence indicates that long-term, low-dose cabergoline use in prolactinoma patients was not associated with an increased risk of cardiac valvulopathy and was associated with high rates of prolactin normalization. We **are very uncertain** about the effect of dopamine agonist therapy on the critical outcomes of tumour shrinkage and the resolution of specific clinical manifestations of hyperprolactinemia as specified in the recommendation. |
| Cho 2009[12] | Retrospective cohort study | 10 treatment-naïve male patients with invasive giant prolactinomas | Serious | Serious | Not serious | Not serious | Very low | This retrospective cohort study reported that serum prolactin fell by a mean of 97% after 3 months (9/10 patients). At a mean follow-up of 19 months, prolactin was reduced by 98%, with normalization achieved in five patients. Tumour volume decreased by 85% on the first follow-up MRI (3-12 months). In patients treated for >12 months, shrinkage reached 97%, which was reported as significantly greater than the 78% reduction in those treated for <12 months. All patients tolerated treatment well with no serious adverse events; visual-field defects improved in three patients, and hypogonadism resolved in three of the seven affected patients.  The certainty of the evidence was rated down by two levels:  **Risk of Bias:** The evidence is derived from a small, retrospective cohort study. This design carries a high risk of selection and information bias, and the lack of a control group limits the reliability of the observed associations.  **Indirectness:** The evidence is indirect regarding the population. The study population is narrowly defined, including only treatment-naïve male patients with invasive giant prolactinomas, a rare and severe subtype. The treatment effects bserved in this extreme subgroup cannot be representative of the broader and more heterogeneous target population of the recommendation, which encompasses all prolactinoma patients.  **Very low certainty evidence** indicates that in a highly selected group of treatment-naïve male patients with invasive giant prolactinomas, cabergoline therapy was associated with substantial reductions in serum prolactin and tumour volume, along with improvements in some clinical symptoms. We **are very uncertain** about the effect of dopamine agonist therapy on the outcomes specified in the recommendation for the broader population of prolactinoma patients, which includes females and those with non-invasive or smaller tumours. |
| Delgrange 2009[13] | Retrospective cohort study | 122 treatment-naïve patients with macroprolactinomas | Serious | Not serious | Not serious | Not serious | Very low | This retrospective cohort study of 122 treatment-naïve patients with macroprolactinomas reported that monotherapy with cabergoline achieved biochemical control in 94% of patients, with significant tumour shrinkage observed in 82%.  The certainty of the evidence was rated down by one level due to **serious risk of bias**: The evidence is derived from a retrospective cohort study. This study design carries a high risk of selection, information, and confounding bias. Data collection relies on past medical records, which may be incomplete or inconsistently recorded, and the lack of a concurrent control group limits the strength of causal inference.  **Very low certainty evidence** from a retrospective cohort study indicates that in treatment-naïve patients with macroprolactinomas, cabergoline monotherapy was associated with high rates of biochemical control and tumour shrinkage. We **are very uncertain** about the effect of dopamine agonist therapy on the full range of clinical manifestations specified in the recommendation, and about its efficacy in patients with microprolactinomas or those who have received prior treatment. |
| Lombardi 2014[14] | Retrospective cohort study | treatment-naïve patients with prolactin-secreting pituitary adenomas | Serious | Not serious | Not serious | Not serious | Very low | This retrospective cohort study reported that after 24 months of treatment, tumour shrinkage (defined as a >4 mm reduction in any MRI dimension) was observed in 102 of 151 cases (67.5%). The study noted that the shrinkage rate was 80.4% when prolactin was persistently suppressed below 5 ng/mL, compared to 63.3% when prolactin merely reached the normal range (p = 0.045).  The certainty of the evidence was rated down by one level due to **serious risk of bias**: The evidence is derived from a retrospective cohort study. This design carries a high risk of selection bias, information bias and confounding. The reliance on historical data and the absence of a concurrent control group significantly limit the reliability of the observed associations.  **Very low certainty evidence** from a retrospective cohort study indicates that in treatment-naïve patients with prolactinomas, dopamine agonist therapy was associated with tumour shrinkage in a majority of patients, and that the degree of shrinkage was greater with more profound prolactin suppression. We **are very uncertain** about the effect of this therapy on serum prolactin levels and the specific clinical manifestations of hyperprolactinemia as outlined in the recommendation. |
| Pala 2015[15] | Non-randomised, prospective cohort study with matched controls | 19 treatment-naïve patients with prolactinomas versus healthy controls | Serious | Not serious | Not serious | Not serious | Very low | This non-randomised, prospective cohort study reported that cabergoline, used as first-line therapy, not only rapidly normalized prolactin levels but also significantly reversed obesity and glucose-lipid metabolic abnormalities associated with hyperprolactinaemia.  The certainty of the evidence was rated down by one level due to **serious risk of bias**: The evidence is derived from a small, non-randomised study. Despite its prospective design and use of matched controls, the lack of randomisation introduces a high risk of selection and confounding bias.  **Very low certainty evidence** from the study indicates that in treatment-naïve patients with prolactinomas, cabergoline therapy was associated with normalization of prolactin levels and improvement in some metabolic parameters. We **are very uncertain** about the effect of dopamine agonist therapy on the outcomes of tumour shrinkage and other clinical manifestations specified in the recommendation. |
| dos Santos Nunes V et al., 2011[16] | Systematic review and meta-analysis | patients with prolactinomas receiving initial or ongoing treatment | Not serious | Serious | Not serious | Not serious | Moderate | This systematic review and meta-analysis reported that dopamine agonists effectively control prolactin levels and tumour-related symptoms. Meta-analysis of comparative studies found cabergoline superior to bromocriptine for achieving prolactin normalization (relative risk [RR]=0.67, 95% CI 0.57-0.80) and resumption of menstruation/ovulation (RR=0.74, 95% CI 0.67-0.83). The review also cited indirect evidence (e.g., a large retrospective series) indicating cabergoline markedly reduces prolactin and shrinks tumour volume. The authors concluded that dopamine agonists are efficacious, with cabergoline demonstrating superior efficacy and tolerability and being recommended as first-line therapy. The meta-analysis included four RCTs.  The overall certainty of the evidence was rated as moderate. This rating was derived by starting at **high certainty** due to the inclusion of several well-conducted randomized controlled trials (RCTs) in the meta-analysis, which provide the most reliable design for assessing intervention effects. The certainty was then downgraded by one level for **indirectness**: While the evidence directly addresses prolactin normalization and some clinical symptoms, the strongest comparative data for the critical outcome of tumour shrinkage is less direct, relying more on large observational series rather than head-to-head RCT comparisons. This introduces some uncertainty in applying the evidence to all outcomes specified in the recommendation.  **Moderate certainty evidence** suggests that dopamine receptor agonist therapy, particularly with cabergoline, is **probably** effective in normalising serum prolactin levels and improving associated clinical symptoms (e.g., restoring menstrual function) in patients with prolactinomas. Cabergoline **likely leads to** better biochemical and clinical outcomes than bromocriptine. Evidence regarding tumour shrinkage is also **probably** in favor of therapy, though this conclusion is less directly supported by the comparative data. |
| Webster J et al., 1994[17] | Randomised controlled trial | women with primary or secondary amenorrhoea due to treatment-naïve or previously treated hyperprolactinaemia | Not serious | Serious | Not serious | Not serious | Moderate | This randomised controlled trial reported that cabergoline was superior to bromocriptine in both efficacy and tolerability. The authors suggested this represents a significant therapeutic advance and that cabergoline should be the first-line dopamine agonist, offering better tolerability, higher adherence, and greater efficacy.  The overall certainty of the evidence was rated as moderate. This rating was derived by starting at high certainty because the evidence comes from a randomised controlled trial. The certainty was then downgraded by one level for **indirectness**: The evidence is indirect regarding the **population and outcomes**. The study population was exclusively women with hyperprolactinaemic amenorrhoea, which does not fully represent all prolactinoma patients. Furthermore, the primary outcome was focused on amenorrhoea and tolerability; it provides only indirect evidence regarding other critical outcomes specified in the recommendation, such as prolactin level reduction and tumour shrinkage.  **Moderate certainty evidence** suggests that in women with hyperprolactinaemic amenorrhoea, cabergoline **likely** leads to better efficacy and is **probably** better tolerated than bromocriptine. The evidence directly supports the superiority of cabergoline for improving this specific clinical manifestation (amenorrhoea). |

References:

1. Wang AT, Mullan RJ, Lane MA, et al. Treatment of hyperprolactinemia: a systematic review and meta-analysis. *Syst Rev*. 2012;1:33. Published 2012 Jul 24. doi:10.1186/2046-4053-1-33
2. Colao A, Di Sarno A, Landi ML, Scavuzzo F, Cappabianca P, Pivonello R, Volpe R, Di Salle F, Cirillo S, Annunziato L, Lombardi G: Macroprolactinoma shrinkage during cabergoline treatment is greater in naive patients than in patients pretreated with other dopamine agonists: a prospective study in 110 patients. J Clin Endocrinol Metab 2000;85:2247–2252.
3. Di Sarno A, Landi ML, Cappabianca P, et al. Resistance to cabergoline as compared with bromocriptine in hyperprolactinemia: prevalence, clinical definition, and therapeutic strategy. J Clin Endocrinol Metab. 2001;86(11):5256-5261. doi:10.1210/jcem.86.11.8054
4. Corsello SM, Ubertini G, Altomare M, Lovicu RM, Migneco MG, Rota CA, Colosimo C: Giant prolactinomas in men: efficacy of cabergoline treatment. Clin Endocrinol (Oxf) 2003;58:662–670.
5. Colao A, Sarno AD, Cappabianca P, Briganti F, Pivonello R, Somma CD, Faggiano A, Biondi B, Lombardi G: Gender differences in the prevalence, clinical features and response to cabergoline in hyperprolactinemia. Eur J Endocrinol 2003;148:325–331.
6. Colao A, Di Sarno A, Cappabianca P, Di Somma C, Pivonello R, Lombardi G: Withdrawal of long-term cabergoline therapy for tumoral and nontumoral hyperprolactinemia. N Engl J Med 2003;349:2023–2033.
7. Colao A, Vitale G, Cappabianca P, Briganti F, Ciccarelli A, De Rosa M, Zarrilli S, Lombardi G: Outcome of cabergoline treatment in men with prolactinoma: effects of a 24-month treatment on prolactin levels, tumor mass, recovery of pituitary function, and semen analysis. J Clin Endocrinol Metab 2004;89:1704–1711.
8. De Rosa M, Ciccarelli A, Zarrilli S, Guerra E, Gaccione M, Di Sarno A, Lombardi G, Colao A: The treatment with cabergoline for 24 months normalizes the quality of seminal fluid in hyperprolactinaemic males. Clin Endocrinol 2006;64:307–313.
9. Shimon I, Benbassat C, Hadani M: Effectiveness of long-term cabergoline treatment for giant prolactinoma: study of 12 men. Eur J Endocrinol 2007;156:225–231.
10. Ono M, Miki N, Kawamata T, Makino R, Amano K, Seki T, Kubo O, Hori T, Takano K: Prospective study of high-dose cabergoline treatment of prolactinomas in 150 patients. J Clin Endocrinol Metab 2008;93:4721–4727.
11. Vallette S, Serri K, Rivera J, Santagata P, Delorme S, Garfield N, Kahtani N, Beauregard H, Aris-Jilwan N, Houde G, Serri O: Long-term cabergoline therapy is not associated with valvular heart disease in patients with prolactinomas. Pituitary 2009;12:153–157.
12. Cho EH, Lee SA, Chung JY, Koh EH, Cho YH, Kim JH, Kim CJ, Kim MS: Efficacy and safety of cabergoline as first line treatment for invasive giant prolactinoma. J Korean Med Sci 2009;24:874–878.
13. Delgrange E, Daems T, Verhelst J, Abs R, Maiter D: Characterization of resistance to the prolactin-lowering effects of cabergoline in macroprolactinomas: a study in 122 patients. Eur J Endocrinol 2009;160:747–752.
14. Lombardi M, Lupi I, Cosottini M, Rossi G, Manetti L, Raffaelli V, Sardella C, Martino E, Bogazzi F: Lower prolactin levels during cabergoline treatment are associated with tumor shrinkage in prolactin-secreting pituitary adenoma. Horm Metab Res 2014;46:939–942.
15. Pala NA, Laway BA, Misgar RA, Dar RA: Metabolic abnormalities in patients with prolactinoma: response to treatment with cabergoline. Diabetol Metab Syndr 2015;7:99.
16. dos Santos Nunes V, El Dib R, Boguszewski C L, et al. Cabergoline versus bromocriptine in the treatment of hyperprolactinemia: a systematic review of randomized controlled trials and meta-analysis[J]. Pituitary, 2011, 14 (3): 259-65. doi: 10.1007/s11102-010-0290-z.
17. Webster J, Piscitelli G, Polli A, et al. A comparison of cabergoline and bromocriptine in the treatment of hyperprolactinemic amenorrhea. Cabergoline Comparative Study Group[J]. N Engl J Med, 1994, 331 (14): 904-9. doi: 10.1056/nejm199410063311403.

**Recommendation 10: For patients with Knosp grade 0 or 1, well-defined microadenomas or macroadenomas, before initiating treatment, the MDT should fully inform the patient of the pros and cons of surgical versus pharmacological treatment options. Combined with patient preference, either surgery or DA can be chosen as the first-line treatment. (Evidence grade: Low, Weak Recommendation)**

**Recommendation 11:** **For patients with Knosp grade≥2 prolactinomas, especially invasive prolactin macroadenomas or giant adenomas, DA therapy is the first choice. (Evidence grade: Low, Weak Recommendation).**

**10: For patients with Knosp grade 0 or 1, well-defined microadenomas or macroadenomas, before initiating treatment, the MDT should fully inform the patient of the pros and cons of surgical versus pharmacological treatment options. Combined with patient preference, either surgery or DA can be chosen as the first-line treatment. (Low certainty evidence).**

| Study ID | Study Design | Population | Risk of Bias | Indirectness | Inconsistency | Publication Bias | Certainty of Evidence | Findings |
| --- | --- | --- | --- | --- | --- | --- | --- | --- |
| Wu Z B et al., 2006[1] | Retrospective cohort study | patients with invasive giant prolactinomas classified as Knosp grade III or IV | Not serious | Not serious | Not serious | Not serious | Low | This retrospective cohort study noted that, while the study did not directly enroll patients with Knosp grade 0 or 1 tumours, it indirectly supports the validity of the recommendation. The study demonstrated that dopamine agonist (DA) therapy was effective in invasive lesions, implying that DA is also likely to benefit non-invasive (Knosp 0-1) tumours, thereby justifying a balanced discussion of both surgical and pharmacological options.  The overall certainty of the evidence supporting this specific aspect of the recommendation (consideration of DA therapy for Knosp 0-1 tumours) was rated as **low**. This initial rating reflects that the direct evidence is absent, and the support is derived indirectly from a study in a different, more severe patient population (invasive tumours). No further upgrading or downgrading was applied based on the provided information.  **Low certainty evidence** suggests that dopamine agonist therapy **may be** effective for invasive prolactinomas. This provides indirect support for the consideration of DA therapy as a viable first-line option for patients with non-invasive (Knosp 0-1) tumours, within a shared decision-making framework that includes surgical options. |
| Chen et al. 2024[2] | Retrospective cohort study | 290 pathologically confirmed prolactinoma patients (treated and untreated) | Serious | Serious | Not serious | Not serious | Very low | This retrospective cohort study reported that surgical resection was highly feasible and curative. Among 217 patients with non-invasive (Knosp 0-2) tumours, 72.8% achieved immediate postoperative endocrine remission (IPR), significantly higher than the 19.2% in Knosp 3-4 lesions. Microadenomas (Knosp 0-1) demonstrated an IPR rate of 91.2%. The study mandated a “multidisciplinary discussion” incorporating patient preferences (e.g., reluctance to long-term medication) and presented both surgery and dopamine agonist (DA) therapy as valid options for Knosp 0-1 tumours. It also reported that DA pretreatment induced significant tumour fibrosis, increased surgical complexity (longer operative time, greater blood loss) and complication rates (19.4% vs. 8.9%), and was associated with a lower IPR rate in microadenomas compared to primary surgery (86.7% vs. 100%). The authors concluded that comprehensive counselling regarding the advantages (high surgical cure rate) and disadvantages (long-term DA dependence, potential fibrosis) of each modality is needed, with decisions guided by patient choice.  The certainty of the evidence was rated down by two levels:  **Risk of Bias:** The evidence is derived from a retrospective cohort study. This design carries a high risk of selection, information, and confounding bias, especially when comparing pre-treated and treatment-naïve groups, as the decision to pretreat was not random and likely influenced by unmeasured factors.  **Indirectness:** The evidence is indirect regarding the **intervention/comparison**. The study primarily describes outcomes in patients who underwent surgery, with or without prior DA exposure. It does not provide a direct, concurrent comparison of the two first-line strategies (primary surgery vs. primary DA therapy) in a population where both are considered equally viable from the outset, as recommended.  **Very low certainty evidence** indicates that in a surgical cohort, patients with non-invasive prolactinomas (Knosp 0-1) achieved high rates of immediate remission after surgery. We **are very uncertain** about the effect of dopamine agonist pretreatment on surgical outcomes (such as complication rates and remission success) compared to primary surgery. We **are also very uncertain** about the comparative long-term effectiveness, safety, and quality-of-life outcomes of primary surgery versus primary DA therapy for patients with Knosp 0-1 tumours, or about how patient preferences should be optimally integrated into this decision. |

**Recommendation 11: For patients with Knosp grade≥2 prolactinomas, especially invasive prolactin macroadenomas or giant adenomas, DA therapy is the first choice. (Low certainty evidence).**

| Study ID | Study Design | Population | Risk of Bias | Indirectness | Inconsistency | Publication Bias | Certainty of Evidence | Findings |
| --- | --- | --- | --- | --- | --- | --- | --- | --- |
| Wu Z B et al., 2006[1] | Retrospective cohort study | patients with invasive giant prolactinomas classified as Knosp grade III or IV | Not serious | Not serious | Not serious | Not serious | Low | This retrospective cohort study involved patients with invasive giant prolactinomas classified as Knosp grade III or IV. The treatment strategy included 14 patients who received bromocriptine (a dopamine agonist, DA) as first-line therapy and 6 patients who received DA after failed surgery. The authors explicitly concluded that “dopamine-agonist medication can serve as first-line therapy for invasive giant prolactinomas” effectively reducing tumour volume, controlling prolactin levels, and obviating the need for unnecessary surgery.  The overall certainty of the evidence for the effectiveness of DA as first-line therapy in this specific, severe subgroup was rated as **low**. This initial rating reflects that the evidence is derived from a single, non-randomized observational study without a concurrent control group. No further upgrading or downgrading was applied based on the provided information.  **Low certainty evidence** suggests that in patients with invasive giant prolactinomas (Knosp III-IV), dopamine agonist (DA) therapy **may be** effective in reducing tumour volume and controlling prolactin levels, and **may** serve as a first-line treatment option. |
| Chen et al. 2024[2] | Retrospective cohort study | 290 pathologically confirmed prolactinoma patients (treated and untreated) | Serious | Not serious | Not serious | Not serious | Very low | This retrospective cohort study reported that surgery for Knosp ≥2 (especially grades 3-4) tumours was associated with low immediate postoperative endocrine remission rates (19.2% for Knosp 3-4) and higher surgical morbidity. The study affirmed that “DA as first-line therapy” represents an international consensus, with surgery reserved for DA failure or emergencies. By excluding DA-responsive patients from the surgical cohort, the study design indirectly supported the pharmacological control of invasive tumours. The authors concluded that surgery for Knosp ≥2 patients is recommended only when DA is ineffective, intolerable, or at patient insistence for debulking, and they explicitly support DA as first-line therapy for these invasive prolactinomas.  The certainty of the evidence was rated down by one level due to **serious risk of bias**: The evidence is derived from a retrospective cohort study with significant methodological limitations. This introduces a high risk of confounding and selection bias, severely limiting the strength of causal inference regarding the effectiveness of DA therapy.  **Very low certainty evidence** indicates that in a surgical cohort, invasive prolactinomas (Knosp≥2) were associated with poor surgical outcomes. We **are very uncertain** about the efficacy, optimal regimen, and patient-important outcomes of dopamine agonist (DA) therapy as a first-line treatment specifically for patients with Knosp≥2 prolactinomas, as the study did not directly evaluate DA in this population and the evidence is based on indirect inference. |
| Billion L, et al. 2023[3] | Retrospective cohort study | Knosp ≥2, invasive giant prolactinomas | Not serious | Not serious | Not serious | Not serious | Low | This retrospective cohort study reported a uniform management approach: 82% of patients received dopamine agonists (DA) as first-line therapy. Among those treated, 88% exhibited tumour shrinkage, 51% achieved prolactin normalization, and 85% experienced visual improvement. Surgery was reserved for specific indications (e.g., DA resistance, CSF leak, apoplexy) and yielded limited efficacy: all operated patients had residual tumour and persistent hyperprolactinaemia. The authors concluded that DA is unequivocally the treatment of choice, irrespective of tumour size or the presence of visual compromise.  The overall certainty of the evidence for the effectiveness of DA as first-line therapy in this specific population (invasive giant prolactinomas, Knosp≥2) was rated as **low**. This initial rating reflects that the evidence is derived from a single, non-randomized observational study without a concurrent control group. No further upgrading or downgrading was applied based on the provided information.  **Low certainty evidence** suggests that in patients with invasive giant prolactinomas (Knosp≥2), dopamine agonist (DA) therapy **may** lead to tumour shrinkage, prolactin normalization, and visual improvement in a substantial proportion of patients. In this severe subgroup, surgery **may be** of limited effectiveness when used after DA failure or for specific complications. |

References:

1. Wu Z B, Yu C J, Su Z P, et al. Bromocriptine treatment of invasive giant prolactinomas involving the cavernous sinus: results of a long-term follow up[J]. J Neurosurg, 2006, 104 (1): 54-61. doi: 10.3171/jns.2006.104.1.54.
2. Chen Z, Shou X, Ji L, et al. Presurgical Medical Treatment in Prolactinomas: Surgical Implications and Pathological Characteristics From 290 Cases[J]. J Clin Endocrinol Metab, 2024, 109 (6): 1433-42. doi: 10.1210/clinem/dgad758.
3. Billion L, Verleye A, Block C, et al. Giant prolactinomas, a detailed analysis of 196 adult cases[J]. Pituitary, 2023, 26 (5): 529-37. doi: 10.1007/s11102-023-01337-0.

**Recommendation 12: For patients with microprolactinomas and hyperprolactinemia but no related clinical manifestations, observation is suggested. Follow-up should include clinical symptoms, PRL levels, and pituitary MRI. Treatment should be initiated if symptoms of hyperprolactinemia appear or if the tumor enlarges. (Evidence grade: Very Low, Weak Recommendation)**

| Study ID | Study Design | Population | Risk of Bias | Indirectness | Inconsistency | Publication Bias | Certainty of Evidence | Findings |
| --- | --- | --- | --- | --- | --- | --- | --- | --- |
| Schlechte J 1989[1] | Prospective cohort study | untreated women with hyperprolactinaemia | Serious | Serious | Not serious | Not serious | Very low | This prospective cohort study followed 30 untreated women with hyperprolactinaemia (presumed microprolactinomas) for a mean of 5.2 years. It reported that none of the patients experienced visual loss, headache, hypopituitarism, or significant tumour enlargement; only two (7%) showed minimal tumour progression without clinical sequelae, and none developed macroadenomas or required surgery. Spontaneous remission occurred in a subset: 35% experienced symptom improvement, prolactin levels normalized in six patients, and imaging abnormalities reverted to normal in four patients. The study concluded that prolactin levels and tumour size changes are not concordant and should not be the sole criteria for initiating therapy, and that annual assessment of clinical status, serum prolactin, and pituitary imaging (CT or MRI) safely and effectively detects early tumour progression.  The certainty of the evidence was rated down by two levels:  **Risk of Bias**: The evidence is from an old (1989), small, single-arm cohort study without a control group. The diagnostic criteria for microprolactinoma were based on imaging available at the time (CT), which is less sensitive than modern MRI. The lack of blinding and potential for selective reporting over long-term follow-up introduce significant risk of bias.  **Indirectness**: The evidence is indirect regarding the **population**. The study included only women with hyperprolactinaemia and presumed microadenomas. The natural history, progression rates, and likelihood of spontaneous remission could differ in male patients with microprolactinomas, limiting the applicability of these findings to the broader population addressed by the recommendation.  **Very low certainty evidence** indicates that in untreated women with presumed microprolactinomas followed for several years, significant clinical or tumour progression was rare, and spontaneous improvement occurred in some. We **are very uncertain** about the safety and long-term outcomes of an observation strategy for all microprolactinoma patients, including men, or about the optimal timing and triggers for initiating treatment. |
| Karunakaran S 2001[2] | Retrospective cohort study | patients with hyperprolactinaemia (including microprolactinomas) | Serious | Serious | Not serious | Not serious | Very low | This retrospective cohort study reported that among 69 women who received neither surgery nor experienced pregnancy or menopause, only 7% required continuous treatment during a median 5-year follow-up. It also noted that prolactin normalized spontaneously in 45% of post-menopausal women and 24% after pregnancy. The authors stated that there was “no significant association between pituitary imaging abnormalities and prolactin normalization” indirectly supporting the notion that tumour enlargement, rather than isolated imaging changes, should trigger treatment.  The certainty of the evidence was rated down by two levels:  **Risk of Bias**: The evidence is derived from a retrospective cohort study. This design carries a high risk of selection, information, and confounding bias, particularly given the exclusion of patients who underwent surgery or experienced pregnancy/menopause, which may limit the generalizability of the findings to an unselected population.  **Indirectness**: The evidence is indirect regarding the population. The study population primarily consisted of women with hyperprolactinaemia, and its findings related to spontaneous normalization are specifically tied to female physiological states (pregnancy, menopause). The applicability of these findings to male patients with microprolactinomas is highly uncertain, limiting the directness of the evidence for the broader recommendation.  **Very low certainty evidence** from a retrospective cohort study indicates that in a selected group of women with hyperprolactinaemia/microprolactinomas not undergoing surgery, most did not require treatment over several years, and spontaneous prolactin normalization occurred in some. We **are very uncertain** about the safety and outcomes of an observation strategy for all microprolactinoma patients, including men, or about the predictive value of imaging changes for guiding treatment decisions. |
| Santharam S et al. 2018[3] | Retrospective cohort study | postmenopausal women with prolactinomas | Serious | Serious | Not serious | Not serious | Very low | This retrospective cohort study reported that microadenomas exhibited low progression rates: only 7% (2/27) of women who discontinued therapy experienced tumour regrowth, with both lesions being microadenomas. Serial prolactin monitoring 6-12 months after discontinuation was predictive of long-term outcome (100% remained normoprolactinaemic if prolactin was normal at that time). The study also observed that estrogen deficiency after menopause may attenuate lactotroph activity, allowing prolactin to decline spontaneously (52% ultimately normalised).  The certainty of the evidence was rated down by two levels:  **Risk of Bias**: The evidence is derived from a retrospective cohort study with a specific selection criterion (patients who discontinued therapy). This design carries a high risk of selection and information bias, and the findings may not be applicable to an unselected, treatment-naïve population.  **Indirectness**: The evidence is indirect regarding the **population**. The study population is narrowly defined as postmenopausal women who had previously been on treatment and then discontinued it. The physiological state of estrogen deficiency is central to the observed outcomes. This provides only indirect, and likely inapplicable, evidence for the recommendation's target population, which includes all patients (including premenopausal women and men) with untreated, asymptomatic microprolactinomas.  **Very low certainty evidence** indicates that in postmenopausal women with microprolactinomas, tumour progression after treatment cessation was infrequent, and prolactin levels at a specific timepoint may predict long-term stability. We are very uncertain about the natural history, safety of observation, and optimal monitoring strategy for untreated microprolactinoma patients, particularly in premenopausal women and men. |
| Constantinescu et al. 2025[4] | Retrospective cohort study | postmenopausal women with prolactinomas | Serious | Serious | Not serious | Not serious | Very low | This retrospective cohort study reported minimal microadenoma progression: among 56 patients, only one (1.8%) exhibited slight regrowth (2 mm). Prolactin levels at 3-6 months after treatment discontinuation were the sole independent predictor of relapse, with no recurrences observed when prolactin was below 0.78 times the upper limit of normal. Most of the 15 reported relapses were asymptomatic; only a minority of patients resumed therapy for symptoms or patient anxiety, while the majority had mild, asymptomatic prolactin increases.  The certainty of the evidence was rated down by two levels:  **Risk of Bias**: The evidence is derived from a retrospective cohort study with inherent risks of selection and information bias. The population consists of patients who had been on treatment and then discontinued it, which cannot represent the natural history of untreated lesions.  **Indirectness**: The evidence is indirect regarding the **population, intervention, and outcomes**. The study focuses on postmenopausal women after treatment discontinuation, which is fundamentally different from the recommendation’s target: all untreated, asymptomatic patients initiating an observation strategy. Furthermore, the primary outcome (relapse after cessation) and the proposed monitoring trigger (post-cessation prolactin level) are not directly applicable to the recommended scenario of de novo observation and monitoring for symptom onset or tumour enlargement.  **Very low certainty evidence** indicates that in postmenopausal women with previously treated microprolactinomas, the risk of significant tumour regrowth after treatment cessation was very low, and early post-cessation prolactin levels may predict relapse. We **are very uncertain** about the progression risk and optimal monitoring strategy for untreated, asymptomatic microprolactinoma patients in the broader population (including premenopausal women and men), or about the clinical significance of asymptomatic prolactin increases. |

References:

1. Schlechte J, Dolan K, Sherman B, et al. The natural history of untreated hyperprolactinemia: a prospective analysis[J]. J Clin Endocrinol Metab, 1989, 68 (2): 412-8. doi: 10.1210/jcem-68-2-412.
2. Karunakaran S, Page R C, Wass J A. The effect of the menopause on prolactin levels in patients with hyperprolactinaemia[J]. Clin Endocrinol (Oxf), 2001, 54 (3): 295-300. doi: 10.1046/j.1365-2265.2001.01190.x.
3. Santharam S, Fountas A, Tampourlou M, et al. Impact of menopause on outcomes in prolactinomas after dopamine agonist treatment withdrawal[J]. Clin Endocrinol (Oxf), 2018, 89 (3): 346-53. doi: 10.1111/cen.13765.
4. Constantinescu S M, Nava C M, Chasseloup F, et al. Menopause has a beneficial influence on the evolution of prolactinomas. A study of 99 patients[J]. J Clin Endocrinol Metab, 2025, doi: 10.1210/clinem/dgaf152.

**Recommendation 13: For patients resistant to bromocriptine, switching to cabergoline is recommended if available. (Evidence grade: Very Low, Weak Recommendation). For patients resistant to cabergoline, discussion by a multidisciplinary team is recommended to choose among surgical treatment, increasing the drug dose to the maximum tolerated level, radiotherapy, or comprehensive treatment including temozolomide (TMZ). (Evidence grade: Very Low, Weak Recommendation)**

**13a: In patients who are resistant to bromocriptine, switching to cabergoline is recommended if cabergoline is available (Very low certainty evidence).**

| Study ID | Study Design | Population | Risk of Bias | Indirectness | Inconsistency | Publication Bias | Certainty of Evidence | Findings |
| --- | --- | --- | --- | --- | --- | --- | --- | --- |
| Huang, H，et.al 2018[1] | Systematic review | patients with macroprolactinomas | Not serious | Serious | Not serious | Not serious | Very low | This systematic review reported that cabergoline (CAB) was superior to bromocriptine (BRC) in normalising prolactin levels (60.4% vs. 35.3%, p=0.01), with the difference more pronounced in men (69.4% vs. 31.7%, p=0.01). The review also noted that CAB demonstrates better tolerability and a longer half-life (allowing weekly dosing). The authors suggested this evidence indirectly supports switching to CAB in BRC-resistant patients, given its greater efficacy and patient acceptability.  The certainty of the evidence was rated down by one level due to **serious indirectness**:  The evidence is indirect regarding the **population**. The review compared the efficacy of CAB versus BRC as initial or primary therapies in a broad population of macroprolactinoma patients. It did not directly study the specific population of interest: patients who have already been treated with and developed resistance to bromocriptine.  **Very low certainty evidence** indicates that in patients with macroprolactinomas, cabergoline demonstrated higher rates of prolactin normalization than bromocriptine in head-to-head comparisons. We **are very uncertain** about the effect of switching from bromocriptine to cabergoline specifically in patients who have developed resistance to bromocriptine, as the evidence is not derived from studies directly in this population. |
| Wang AT et al., 2012[2] | Systematic review and meta-analysis | >3 000 patients diagnosed with hyperprolactinaemia (including micro- and macro-prolactinomas) | Not serious | Serious | Not serious | Not serious | Very low | This systematic review and meta-analysis reported that pooled data from six observational studies and three RCTs showed cabergoline was superior to bromocriptine in resolving persistent hyperprolactinaemia, amenorrhoea/oligomenorrhoea, and galactorrhoea, with significantly fewer and milder side-effects (e.g., nausea, hypotension).  The certainty of the evidence was rated down by one level due to **serious indirectness**:  The evidence is indirect regarding the **population**. The meta-analysis compared cabergoline to bromocriptine as initial or primary therapies in a general population of hyperprolactinaemic/prolactinoma patients. It does not provide direct evidence on the effectiveness of switching therapy in the specific subgroup of patients who have already been treated with and developed resistance to bromocriptine.  **Very low certainty evidence** indicates that in a broad population of patients with hyperprolactinaemia or prolactinomas, cabergoline demonstrated superior efficacy and better tolerability than bromocriptine in comparative studies. We **are very uncertain** about the effect of switching from bromocriptine to cabergoline specifically in patients who have developed resistance to bromocriptine, as the evidence is not derived from studies directly in this population. |

References:

1. Huang, H.Y., Lin, S.J., Zhao, W.G. et al. Cabergoline versus bromocriptine for the treatment of giant prolactinomas: A quantitative and systematic review. Metab Brain Dis 33, 969-976 (2018).
2. Wang AT, Mullan RJ, Lane MA, et al. Treatment of hyperprolactinemia: a systematic review and meta-analysis. Syst Rev. 2012;1:33. Published 2012 Jul 24.

**13b: For patients resistant to cabergoline, discussion by a multidisciplinary team is recommended to choose among surgical treatment, increasing the drug dose to the maximum tolerated level, radiotherapy, or comprehensive treatment including temozolomide (TMZ) (Very low certainty evidence).**

| Study ID | Study Design | Population | Risk of Bias | Indirectness | Inconsistency | Publication Bias | Certainty of Evidence | Findings |
| --- | --- | --- | --- | --- | --- | --- | --- | --- |
| Losa et al., 2016[1] | Retrospective multicentre survey | 31 patients with aggressive pituitary adenomas or carcinomas resistant to conventional therapies | Serious | Serious | Not serious | Not serious | Very low | This retrospective multicentre survey reported that temozolomide (TMZ) was used as salvage therapy for tumours refractory to standard treatments, including dopamine agonists such as cabergoline. Among the five patients with prolactin-secreting adenomas resistant to dopamine agonists, TMZ resulted in tumour shrinkage in two cases, stable disease in two, and progression in one. The authors emphasised multidisciplinary comprehensive management, noting all patients had undergone prior surgery (100%) and/or radiotherapy (87.1%) before receiving TMZ. Treatment options for recurrent disease included repeat surgery, radiotherapy, and various medical therapies, aligning with the principle of individualised multimodal therapy.  The certainty of the evidence was rated down by two levels:  **Risk of Bias**: The evidence is derived from a retrospective, uncontrolled case series with a very small sample size specific to prolactinomas. This design carries an extremely high risk of selection, reporting, and confounding bias, severely limiting the reliability of the findings.  **Indirectness**: The evidence is indirect regarding the **population and intervention**. The study population primarily consisted of patients with aggressive pituitary adenomas or carcinomas, which are distinct from the more common cabergoline-resistant prolactinomas. The interventions were applied in a salvage, multimodal setting after multiple prior treatments, not as direct comparisons of the specific management options outlined in the recommendation for a less advanced, resistant population.  **Very low certainty evidence** indicates that in a highly selected population of patients with aggressive, resistant pituitary tumours, including a few with prolactinomas, temozolomide (TMZ) therapy was associated with some tumour responses. We **are very uncertain** about the efficacy and safety of TMZ, dose escalation, repeat surgery, or radiotherapy specifically for patients with cabergoline-resistant prolactinomas, or about the optimal process for multidisciplinary decision-making in this context. |

References:

1. M. Losa, F. Bogazzi, S. Cannavo, et al. Temozolomide therapy in patients with aggressive pituitary adenomas or carcinomas, J Neurooncol, 126 (2016) 519-525.

**Recommendation 14: Patients receiving long-term, high-dose cabergoline therapy are recommended to undergo periodic echocardiography to assess cardiac valve status. (Evidence grade: Low, Weak Recommendation)**

| Study ID | Study Design | Population | Risk of Bias | Indirectness | Inconsistency | Publication Bias | Certainty of Evidence | Findings |
| --- | --- | --- | --- | --- | --- | --- | --- | --- |
| Caputo C et al., 2015[1] | Meta-analysis + retrospective study | patients with invasive giant prolactinomas classified as Knosp grade III or IV | Not serious | Not serious | Not serious | Not serious | Low | This meta-analysis and retrospective study reported a very low overall incidence of valvular lesions (0.11%). The risk of cardiac valve disease was increased only in patients receiving high-dose (>3 mg/week) or long-term (>5 years) cabergoline, supporting echocardiographic screening in this high-risk subgroup.  The overall certainty of the evidence regarding the association between cabergoline and valvular risk was rated as **low**. This initial rating reflects that the evidence is primarily derived from observational studies (included in the meta-analysis), which are subject to confounding and bias.  **Low certainty evidence** from a meta-analysis suggests that the risk of cardiac valve disease in prolactinoma patients treated with cabergoline **may be** very low overall but **may be** increased in those receiving high-dose (>3 mg/week) or long-term (>5 years) therapy. The need for routine echocardiographic screening in all patients is unclear, but it **may be** considered in this high-risk subgroup. |

References:

1. Caputo C, Prior D, Inder W J. The need for annual echocardiography to detect cabergoline-associated valvulopathy in patients with prolactinoma: a systematic review and additional clinical data[J]. Lancet Diabetes Endocrinol, 2015, 3 (11): 906-13. doi: 10.1016/s2213-8587(14)70212-8.

**Recommendation 15: For microadenoma patients receiving DA therapy, after PRL levels normalize, continuing the current dose for 3–6 months is suggested (Evidence grade: Low, Weak Recommendation). Subsequently, when PRL levels remain within the reference range, gradual dose reduction can be considered, monitoring PRL levels, and titrating the dose down to the minimum necessary to maintain normal PRL (Evidence grade: Very Low, Weak Recommendation). For macroadenoma patients, drug dose reduction should be assessed in conjunction with tumor shrinkage (Evidence grade: Low, Weak Recommendation).**

**Recommendation 16: For prolactinoma patients on low-maintenance dose DA therapy for more than 2 years, with normal PRL levels and post-treatment imaging showing significant tumor shrinkage or no significant residual lesion, drug withdrawal can be considered. (Evidence grade: Very Low, Weak Recommendation)**

**Recommendation 17: Female prolactinoma patients who reach the above withdrawal criteria after menopause are encouraged to attempt drug withdrawal and follow-up. If pituitary lesion enlargement is found during follow-up, DA therapy can be restarted after evaluation. (Evidence grade: Low, Weak Recommendation)**

**15a: For microadenoma patients receiving DA therapy, after PRL levels normalize, continuing the current dose for 3-6 months is suggested (Low certainty evidence).**

| Study ID | Study Design | Population | Risk of Bias | Indirectness | Inconsistency | Publication Bias | Certainty of Evidence | Findings |
| --- | --- | --- | --- | --- | --- | --- | --- | --- |
| Xia MY, et al. 2018[1] | Systematic review + Meta analysis | Patients with hyperprolactinaemia receiving dopamine-agonist therapy | Not serious | Not serious | Not serious | Not serious | Low | This systematic review and meta-analysis reported that the success rate of stopping therapy after≥24 months of cabergoline treatment was significantly higher than after 3-6 months (48.7% vs. 20%). The authors noted that while 3-6 months of consolidation therapy may reduce early relapse, the evidence better supports prolonging treatment beyond 2 years, especially with cabergoline.  The overall certainty of the evidence regarding the optimal consolidation therapy duration was rated as **low**. This initial rating reflects that the evidence is derived from a systematic review and meta-analysis of observational studies and a limited number of trials, which are subject to potential confounding and heterogeneity. No further upgrading or downgrading was applied based on the provided information.  **Low certainty evidence** suggests that continuing dopamine agonist therapy for 3-6 months after prolactin normalization **may** reduce early relapse compared to stopping immediately, but longer treatment durations (≥24 months) **may** lead to higher success rates for sustained remission after drug withdrawal. |
| Dekkers et al., 2010[2] | Systematic review + Meta analysis | Hyperprolactinaemic patients treated with dopamine agonists | Not serious | Not serious | Not serious | Not serious | Low | This systematic review and meta-analysis noted that all included studies required patients to have received dopamine-agonist therapy for at least 3 months with normalised prolactin (PRL) levels before withdrawal, as an inclusion criterion. The authors concluded that maintaining the original dose for 3-6 months therefore aligns with published standards and constitutes a reasonable transition before dose tapering.  The overall certainty of the evidence regarding the benefit of a 3-6 month consolidation phase was rated as **low**. This initial rating reflects that the evidence supporting this specific duration is indirect, derived from the common inclusion criteria of withdrawal studies rather than from direct comparative trials evaluating different consolidation lengths.  **Low certainty evidence** suggests that continuing dopamine agonist therapy at the current dose for 3-6 months after prolactin normalisation **may be** a reasonable and commonly applied practice before attempting dose reduction or withdrawal, as it aligns with the baseline criteria used in withdrawal studies. |

**15b: For microadenoma patients receiving DA therapy…Subsequently, when PRL levels remain within the reference range, gradual dose reduction can be considered, monitoring PRL levels, and titrating the dose down to the minimum necessary to maintain normal PRL (Very low certainty evidence).**

| Study ID | Study Design | Population | Risk of Bias | Indirectness | Inconsistency | Publication Bias | Certainty of Evidence | Findings |
| --- | --- | --- | --- | --- | --- | --- | --- | --- |
| Xia MY, et al. 2018[1] | Systematic review + Meta analysis | Patients with hyperprolactinaemia receiving dopamine-agonist therapy | Not serious | Not serious | Not serious | Not serious | Low | This systematic review and meta-analysis of patients with hyperprolactinaemia receiving dopamine-agonist therapy identified “low-dose cabergoline maintenance (≤0.5 mg/week)” as an independent positive predictor for successful therapy withdrawal (success rate 51.5% vs. 21.5%) and emphasised gradual tapering rather than abrupt withdrawal.  The overall certainty of the evidence regarding the optimal dose reduction strategy was rated as **low**. This initial rating reflects that the evidence is derived from a systematic review and meta-analysis of observational studies, which are subject to potential confounding and cannot establish causality.  **Low certainty evidence** suggests that in patients with hyperprolactinaemia, a strategy of gradual dose reduction to a low maintenance dose (e.g., cabergoline≤0.5 mg/week) **may** be associated with higher rates of successful long-term remission after drug withdrawal compared to abrupt cessation or higher maintenance doses. |
| Dekkers et al., 2010[2] | Systematic review + Meta analysis | Hyperprolactinaemic patients treated with dopamine agonists | Not serious | Not serious | Not serious | Not serious | Low | This systematic review and meta-analysis reported that longer treatment duration (>24 months) was associated with a higher probability of sustained normoprolactinaemia after discontinuation (meta-regression P = 0.015). The review also cited a specific study (Colao et al., 2007) in which patients were reduced to the lowest effective dose for 12 months before attempted withdrawal, a strategy consistent with stepwise dose titration.  The overall certainty of the evidence regarding the optimal strategy for dose reduction and withdrawal was rated as **low**. This initial rating reflects that the evidence is derived from a systematic review and meta-analysis of observational studies, and the association between longer treatment and better outcomes may be influenced by confounding factors.  **Low certainty evidence** suggests that in patients with hyperprolactinaemia, longer durations of dopamine agonist therapy (>24 months) **may be** associated with higher rates of sustained remission after drug withdrawal. A strategy of gradual dose reduction to the lowest effective dose for a period before attempting full withdrawal **may be** used, although the optimal duration of this low-dose maintenance phase is unclear. |
| Hu J 2015[3] | Systematic review + Meta analysis | Prolactinoma patients who received cabergoline and achieved successful drug withdrawal | Not serious | Serious | Not serious | Not serious | Very low | This systematic review and meta-analysis reported that meta-regression showed dose reduction to the minimum effective dose was significantly linked to successful withdrawal (P = 0.006). Subgroup analysis indicated a relapse rate of 50% (95% CI 35–64%) in patients tapered to the minimum dose, compared to 73% (95% CI 65–81%) in those not tapered.  The certainty of the evidence was rated down by one level due to **serious indirectness**:  The evidence is indirect regarding the **population**. The meta-analysis included prolactinoma patients of various sizes (micro- and macroadenomas) who were eligible for drug withdrawal. The findings on dose reduction cannot be directly applicable to the specific subpopulation of interest: patients with microprolactinomas.  **Very low certainty evidence** indicates that in patients with prolactinomas who initially responded to cabergoline and underwent withdrawal, those whose dose was reduced to a minimum before cessation experienced a lower relapse rate than those who did not undergo such tapering. We **are very uncertain** about the effect of this gradual dose reduction strategy specifically for patients with microadenomas, as the evidence is not derived from studies focused exclusively on this subgroup. |

**15c:** **For macroadenoma patients, drug dose reduction should be assessed in conjunction with tumor shrinkage (Low certainty evidence).**

| Study ID | Study Design | Population | Risk of Bias | Indirectness | Inconsistency | Publication Bias | Certainty of Evidence | Findings |
| --- | --- | --- | --- | --- | --- | --- | --- | --- |
| Xia MY, et al. 2018[1] | Systematic review + Meta analysis | Patients with hyperprolactinaemia receiving dopamine-agonist therapy | Not serious | Not serious | Not serious | Not serious | Low | This systematic review and meta-analysis of patients with hyperprolactinaemia receiving dopamine-agonist therapy reported that patients with >50% tumour-volume reduction had significantly higher withdrawal-success rates (49.4% vs. 22.1%), and multivariate analysis identified tumour shrinkage as an independent predictor.  The overall certainty of the evidence regarding tumour shrinkage as a predictor for successful withdrawal was rated as **low**. This initial rating reflects that the evidence is derived from a systematic review and meta-analysis of observational studies, which are subject to potential confounding.  **Low certainty evidence** suggests that in patients with prolactinomas, substantial tumour shrinkage (>50%) during dopamine agonist therapy **may be** associated with a higher likelihood of successful drug withdrawal. |
| Dekkers et al., 2010[2] | Systematic review + Meta analysis | Hyperprolactinaemic patients treated with dopamine agonists | Not serious | Not serious | Not serious | Not serious | Low | This systematic review and meta-analysis reported that the literature consistently designates≥50% tumour-volume reduction as a key predictor of successful drug discontinuation. The review cited a specific study (Colao et al., 2007) in which≥50% MRI-documented shrinkage was a mandatory prerequisite for attempted withdrawal.  The overall certainty of the evidence regarding the predictive value of tumour shrinkage for withdrawal success was rated as **low**. This initial rating reflects that the evidence is derived from a systematic review and meta-analysis of observational studies, and the association may be influenced by confounding factors.  **Low certainty evidence** suggests that in patients with prolactinomas, achieving substantial tumour shrinkage (≥50%) during dopamine agonist therapy **may be** associated with a higher probability of successful drug withdrawal, and is commonly used as a criterion before attempting dose reduction or cessation. |

**16: For prolactinoma patients on low-maintenance dose DA therapy for more than 2 years, with normal PRL levels and post-treatment imaging showing significant tumor shrinkage or no significant residual lesion, drug withdrawal can be considered. (Very low certainty evidence)**

| Study ID | Study Design | Population | Risk of Bias | Indirectness | Inconsistency | Publication Bias | Certainty of Evidence | Findings |
| --- | --- | --- | --- | --- | --- | --- | --- | --- |
| Xia MY, et al. 2018[1] | Systematic review + Meta analysis | Patients with hyperprolactinaemia receiving dopamine-agonist therapy | Not serious | Serious | Not serious | Not serious | Very low | This systematic review and meta-analysis reported that low-dose cabergoline maintenance (≤0.5 mg/week) was identified as an independent predictor of successful withdrawal (success rate 51.5% vs. 21.5%). Treatment duration >2 years (≥24 months) with cabergoline was also associated with significantly higher withdrawal success rates (48.7% vs. 20.8%). Furthermore, patients achieving >50% tumour volume reduction had a 49.4% success rate, and this degree of shrinkage was an independent predictor.  The certainty of the evidence was rated down by one level due to **serious indirectness**: The evidence is indirect regarding the **population**. It does not provide direct evidence on the outcomes of withdrawing therapy specifically in the narrowly defined population of interest.  **Very low certainty evidence** indicates that in patients with hyperprolactinaemia who are candidates for drug withdrawal, treatment with low-dose cabergoline, for a duration longer than 2 years, and with substantial tumour shrinkage are factors associated with higher success rates in the studies reviewed. We **are very uncertain** about the effect of withdrawing therapy specifically in patients who meet all three criteria simultaneously, as the evidence is not derived from studies prospectively evaluating this precise population and management strategy. |
| Dekkers et al., 2010[2] | Systematic review + Meta analysis | Hyperprolactinaemic patients treated with dopamine agonists | Not serious | Serious | Not serious | Not serious | Very low | This systematic review and meta-analysis reported that treatment duration >24 months was significantly associated with sustained normoprolactinaemia after withdrawal (meta-regression P = 0.015). All included studies required on-treatment prolactin normalisation as a prerequisite for discontinuation. The review highlighted that in one key study (Colao et al., 2007), ≥50% MRI-documented tumour reduction was mandatory for attempted withdrawal, and a protocol of tapering to the minimum effective dose followed by 12 months of stable low-dose therapy was used.  The certainty of the evidence was rated down by one level due to **serious indirectness**:  The evidence is indirect regarding the **population**. It does not provide direct evidence on the outcomes of withdrawing therapy in the precise population defined by the recommendation.  **Very low certainty evidence** indicates that in studies of hyperprolactinaemic patients attempting drug withdrawal, longer treatment duration (>2 years), on-treatment prolactin normalisation, and substantial tumour shrinkage were commonly used as eligibility criteria or were associated with better outcomes. We **are very uncertain** about the success rate and safety of drug withdrawal specifically in prolactinoma patients who meet the composite criteria of long-term (>2 years), low-dose maintenance therapy with normal PRL and significant imaging response, as the evidence is not derived from studies prospectively evaluating this specific scenario |

**17: Female prolactinoma patients who reach the above withdrawal criteria after menopause are encouraged to attempt drug withdrawal and follow-up. If pituitary lesion enlargement is found during follow-up, DA therapy can be restarted after evaluation. (Low certainty evidence, weak recommendation).**

| Study ID | Study Design | Population | Risk of Bias | Indirectness | Inconsistency | Publication Bias | Certainty of Evidence | Findings |
| --- | --- | --- | --- | --- | --- | --- | --- | --- |
| Xia MY, et al. 2018[1] | Systematic review + Meta analysis | Patients with hyperprolactinaemia receiving dopamine-agonist therapy | Not serious | Not serious | Not serious | Not serious | Low | This systematic review and meta-analysis noted that the review did not stratify outcomes by age, sex, or menopausal status, and data specific to drug discontinuation in post-menopausal women were lacking. All included studies mandated follow-up of at least 6 months (median 12–57 months), and patients with recurrence (tumour growth or rising prolactin) resumed DA therapy, consistent with clinical practice.  The overall certainty of the evidence supporting the recommendation for post-menopausal women specifically was rated as **low**.  **Low certainty evidence** suggests that in studies of dopamine agonist withdrawal, patients who experienced recurrence (tumour growth or rising prolactin) were typically retreated with DA. However, the specific success rates, risks, and optimal follow-up strategy for drug withdrawal **may** differ in post-menopausal women with prolactinomas, as the evidence is not stratified for this subgroup. |
| Dekkers et al., 2010[2] | Systematic review + Meta analysis | Hyperprolactinaemic patients treated with dopamine agonists | Not serious | Not serious | Not serious | Not serious | Low | This systematic review and meta-analysis reported a high relapse rate (79%) following drug withdrawal. The authors found no evidence of negative long-term impacts from discontinuation attempts. The review did not perform a subgroup analysis based on menopausal status, thus not providing specific evidence on the safety or success of withdrawal for post-menopausal women.  **Low certainty evidence** suggests that in hyperprolactinemic patients, attempting dopamine agonist withdrawal **may** be associated with a high rate of recurrence, but therapy **may** be safely restarted if relapse occurs. |

References:

1. Xia M Y, Lou X H, Lin S J, et al. Optimal timing of dopamine agonist withdrawal in patients with hyperprolactinemia: a systematic review and meta-analysis[J]. Endocrine, 2018, 59 (1): 50-61. doi: 10.1007/s12020-017-1444-9.
2. Dekkers O M, Lagro J, Burman P, et al. Recurrence of hyperprolactinemia after withdrawal of dopamine agonists: systematic review and meta-analysis[J]. J Clin Endocrinol Metab, 2010, 95 (1): 43-51. doi: 10.1210/jc.2009-1238.
3. Hu J, Zheng X, Zhang W, et al. Current drug withdrawal strategy in prolactinoma patients treated with cabergoline: a systematic review and meta-analysis[J]. Pituitary, 2015, 18 (5): 745-51. doi: 10.1007/s11102-014-0617-2.

**Recommendation 18: After drug withdrawal according to criteria, continued follow-up for related symptoms (menstrual cycle, galactorrhea, sexual function, etc.) is recommended. PRL levels should be checked every 3 months in the first year after withdrawal, then annually, or whenever symptoms appear. Pituitary MRI should be repeated if clinical symptoms appear alongside persistently elevated PRL. Patients who relapse after withdrawal can restart DA therapy. For those without clinical symptoms and no tumor growth on imaging, observation and follow-up can be chosen. (Evidence grade: Very Low, Weak Recommendation)**

| Study ID | Study Design | Population | Risk of Bias | Indirectness | Inconsistency | Publication Bias | Certainty of Evidence | Findings |
| --- | --- | --- | --- | --- | --- | --- | --- | --- |
| Espinosa-Cárdenas E et al. 2020[1] | Retrospective cohort study | prolactinoma patients who discontinued cabergoline after≥2 years of treatment | Serious | Serious | Not serious | Not serious | Very low | This single-center retrospective cohort study reported on a post-withdrawal surveillance and management protocol. The protocol mandated prolactin measurement every 3-6 months for 1-3 years and MRI every 6 months in the first year, then annually. The study found that, over a median 30-month follow-up, all patients who did not restart therapy were asymptomatic (e.g., no menstrual disturbance, galactorrhea, or sexual dysfunction) and showed no further prolactin rise or tumor enlargement. The authors reported that the decision to restart therapy was individualized, based on the presence of clinical symptoms (e.g., menstrual disturbance, sexual dysfunction) combined with elevated prolactin levels, sometimes confirmed by MRI. Among 34 patients with biochemical relapse, only 8 resumed cabergoline; symptoms improved and no tumor progression occurred after restart. The study concluded that observation without treatment was acceptable for patients without clinical symptoms or significant tumor growth.  The certainty of the evidence was rated down by two levels to very low due to:  **Risk of Bias**: The retrospective, single-center, observational design carries a high risk of confounding, selection bias, and information bias, limiting confidence in the causal relationship between the follow-up strategy and the reported outcomes.  **Indirectness**: The study **population** comprised patients who had already met the study's specific withdrawal criteria (≥2 years of cabergoline treatment). This provides only indirect evidence for the applicability of the findings to the broader population addressed in the recommendation, which includes patients who withdraw based on other sets of "criteria" and may have different baseline risks.  **Very low certainty evidence** indicates that in a cohort of prolactinoma patients meeting criteria for cabergoline withdrawal, a structured follow-up protocol based on symptom assessment, periodic prolactin testing, and scheduled MRI were implemented. We **are very uncertain** about the effect of this specific follow-up strategy on critical outcomes (e.g., prevention of clinical harm, optimal timing for restarting therapy) compared to other approaches, or about its applicability to all patient subgroups. |
| Sosa-Eroza E et al. 2023[2] | Systematic review | prolactinoma patients treated with dopamine agonists | Serious | Serious | Not serious | Not serious | Very low | This systematic review reported on strategies for post-withdrawal management. The authors documented that: 1) Monitoring practices included assessment for symptoms such as amenorrhea, galactorrhea, and loss of libido or erectile dysfunction. In a subgroup of 26 patients who experienced biochemical relapse but did not restart therapy, management decisions were based on the absence of clinical symptoms. 2) A proposed monitoring schedule involved measuring prolactin levels every 3 months in the first year after drug withdrawal, followed by annual checks or assessments triggered by symptom emergence, based on the observation that prolactin elevation often precedes detectable tumor growth. 3) The review noted that tumor regrowth is uncommon and is usually associated with a significant increase in prolactin; therefore, the authors suggested that repeat pituitary MRI be considered when clinical symptoms are present alongside persistently elevated prolactin levels. 4) The literature indicated that restarting dopamine agonist therapy is an established approach for managing symptomatic relapse, and that a second attempt at drug withdrawal has been reported with subsequent relapse rates similar to the first withdrawal. 5) For patients without clinical symptoms and without evidence of significant tumor growth on imaging, the review reported that an observational approach has been used, and that biochemical relapse in isolation has not been uniformly linked to an immediate requirement for treatment re-initiation.  The certainty of the evidence was rated down by two levels to **Very Low** due to the following:  **Risk of Bias**: The evidence is derived from a systematic review of predominantly observational studies, which inherently carry a high risk of selection, performance, and detection bias.  **Indirectness**: The studied **populations** and management strategies were heterogeneous and not explicitly aligned with the structured, criteria-driven withdrawal and monitoring steps outlined in the recommendation. This creates uncertainty in applying the findings directly to the recommended clinical algorithm.  **Very low certainty evidence** indicates that in prolactinoma patients after dopamine agonist withdrawal, monitoring for specific symptoms and prolactin levels is reported in the literature, and that restarting therapy is a documented response to symptomatic relapse. We **are very uncertain** about the effect of following a specific monitoring schedule (e.g., PRL every 3 months then annually) or a symptom-driven MRI strategy on clinical outcomes, compared to alternative follow-up approaches. |

Reference:

1. Espinosa-Cárdenas E, Sánchez-García M, Ramírez-Rentería C, et al. High biochemical recurrence rate after withdrawal of cabergoline in prolactinomas: is it necessary to restart treatment? [J]. Endocrine, 2020, 70 (1): 143-9. doi: 10.1007/s12020-020-02388-0.
2. Sosa-Eroza E, Espinosa-Cárdenas E. Long-term Discontinuation of Dopamine Agonist Treatment in Patients with Prolactinomas Revisited[J]. Arch Med Res, 2023, 54 (8): 102893. doi: 10.1016/j.arcmed.2023.102893.

**3.2 Surgical Management**

**Recommendation 19: Decisions regarding surgical treatment should be made after comprehensive evaluation and fully respecting the patient's preferences. (Good Practice Statement)**

**Recommendation 20: Surgical procedures should be performed by an experienced pituitary surgery team. (Good Practice Statement)**

**Rationale for Good Practice Statement:**

1. Clarity and feasibility: Six key variables (tumour size/morphology, prolactin level, drug response, systemic condition, patient preference, fertility wishes) must be simultaneously weighed; the process—MDT meeting minutes→shared decision-making with the patient→written treatment plan—is explicitly defined.

2. Necessity: Surgical decisions reached after MDT discussion are more readily accepted by patients.

3. Substantial and unequivocal net benefit: The MDT-plus-shared-decision model uses existing hospital resources without additional cost.

4. Prohibitive opportunity cost for direct evidence: Integrating multiple dimensions of extreme patient heterogeneity renders an RCT virtually unfeasible.

5. Clear evidence chain: Tumour burden and drug response determine surgical necessity; systemic status and fertility needs dictate timing and modality; patient values influence acceptance of these trade-offs; the MDT model integrates this information simultaneously and reduces decision bias.

**Recommendation 21: For well-defined microadenomas or macroadenomas (Knosp grade 0 or 1), surgical treatment can be considered as a first-line treatment option. (Evidence grade: Moderate, Weak Recommendation)**

| Study ID | Study Design | Population | Risk of Bias | Indirectness | Inconsistency | Publication Bias | Certainty of Evidence | Findings |
| --- | --- | --- | --- | --- | --- | --- | --- | --- |
| Baussart et al. 2021[1] | Retrospective cohort study | Patients with prolactinoma microadenomas | Not serious | Not serious | Not serious | Not serious | Low | The study reported that among patients with Knosp grade 0-1 tumors (well-defined margins), surgery performed by an experienced team resulted in a remission rate of 88% and a complication rate of 4%.  The certainty of the evidence is rated as **Low**.  **Low certainty evidence** suggests that surgery performed by an experienced team **may** achieve high remission and low complication rates in patients with well-defined (Knosp grade 0-1) prolactin microadenomas. This indicates surgical treatment could be a first-line option for this specific population. |
| Giese et al. 2021[2] | Retrospective cohort study | Prolactinoma patients undergoing first-time transsphenoidal microsurgery | Not serious | Not serious | Not serious | Not serious | Low | The study reported that for both micro- and macroprolactinomas with Knosp grades 0–1 (well-defined margins), surgical outcomes were excellent (remission rates: 92.1% for microadenomas, 70.4% for macroadenomas) with minimal complications, and that patient preference was noted as a legitimate indication for this approach.  The certainty of the evidence is rated as **Low**.  **Low certainty evidence** suggests that for patients with well-defined (Knosp grade 0-1) prolactinomas, transsphenoidal surgery may yield high remission rates with minimal complications. This supports considering surgical treatment as a first-line option for this population, with decision-making incorporating patient preference. |
| Force et al. 2022[3] | Retrospective cohort study | Prolactinoma patients undergoing endoscopic endonasal transsphenoidal surgery | Not serious | Not serious | Not serious | Not serious | Low | The study reported high early remission rates: 91.3% (21/23) for microadenomas (Knosp grade 0) and 65.4% (17/26) for macroadenomas (Knosp grades 0-2), with the rate for Knosp grade 0 macroadenomas being 64.3% (9/14). The authors noted a clear correlation between lower Knosp grade (0-2, well-defined) and higher early remission rates compared to invasive (Knosp 3A/4) tumors, with a low overall complication rate (e.g., 1.7% permanent diabetes insipidus, no CSF leaks) and short hospital stay.  The certainty of the evidence is rated as **Low**.  **Low certainty evidence** suggests that endoscopic surgery **may** achieve high early remission rates and a low complication profile in patients with well-defined (Knosp grade 0-1) prolactinomas, particularly for microadenomas. This supports considering surgical treatment as a first-line option for this population. |
| Zamanipoor et al. 2020[4] | Systematic review + Meta analysis | Patients diagnosed with prolactinoma who received either dopamine agonist therapy or transsphenoidal surgery | Not serious | Not serious | Not serious | Not serious | Moderate | The analysis reported an approximate long-term remission rate of 83% following surgery for microadenomas, compared to a 36% long-term remission rate following dopamine agonist discontinuation in the medication group.  The certainty of this evidence was upgraded from an initial rating of **Low** (due to the risk of bias inherent in observational studies) to **Moderate**, based on the following GRADE criterion: The evidence derives from non-randomized comparisons. The large magnitude of effect observed (absolute risk difference ~47% in favor of surgery) is considered robust. Any plausible uncontrolled confounding or selection bias in these studies (e.g., surgeons potentially selecting healthier patients or those with more favorable tumor anatomy for surgery, which would bias results against surgery if such patients were actually assigned to the medication group) **would likely have underestimated** the true treatment benefit of surgery. Therefore, the true effect is likely to be at least as large as, if not larger than, the estimate provided by this evidence.  Moderate certainty evidence suggests that for patients with prolactinoma, surgery **probably** results in a higher long-term remission rate compared to treatment with dopamine agonists followed by withdrawal. |
| Kreutzer et al. 2008[5] | Retrospective cohort study | Prolactinoma patients treated with transsphenoidal surgery | Not serious | Not serious | Not serious | Not serious | Low | The study reported that long-term remission after surgery for microadenomas was 84.8%, with a recurrence rate of 7.1% and minimal complications. For purely intrasellar microadenomas, postoperative remission was 87.2% with 5.6% recurrence. The study also noted that non-invasive (purely intrasellar) macroadenomas achieved 72.5% postoperative remission, and that surgery achieved 80% efficacy in cystic prolactinomas, a subtype reported to respond poorly to dopamine agonists.  The certainty of the evidence is rated as **Low**.  **Low certainty evidence** suggests that transsphenoidal surgery **may** achieve high long-term remission rates with low recurrence in patients with well-defined (purely intrasellar or non-invasive) prolactinomas, particularly microadenomas. This supports considering surgical treatment as a first-line option for this specific patient population. |

References:

1. Baussart B, Villa C, Jouinot A, et al. Pituitary surgery as alternative to dopamine agonists treatment for microprolactinomas: a cohort study[J]. Eur J Endocrinol, 2021, 185 (6): 783-91. doi: 10.1530/eje-21-0293.
2. Giese S, Nasi-Kordhishti I, Honegger J. Outcomes of Transsphenoidal Microsurgery for Prolactinomas - A Contemporary Series of 162 Cases[J]. Exp Clin Endocrinol Diabetes, 2021, 129 (3): 163-71. doi: 10.1055/a-1247-4908.
3. Force B K, Staggers K, Sebastian S, et al. Endoscopic Endonasal Transsphenoidal Surgery for Patients with Prolactinomas: Indications and Outcomes[J]. World Neurosurg, 2022, 168 e626-e35. doi: 10.1016/j.wneu.2022.10.043.
4. Zamanipoor Najafabadi A H, Zandbergen I M, de Vries F, et al. Surgery as a Viable Alternative First-Line Treatment for Prolactinoma Patients. A Systematic Review and Meta-Analysis[J]. J Clin Endocrinol Metab, 2020, 105 (3): e32-41. doi: 10.1210/clinem/dgz144.
5. Kreutzer J, Buslei R, Wallaschofski H, et al. Operative treatment of prolactinomas: indications and results in a current consecutive series of 212 patients[J]. Eur J Endocrinol, 2008, 158 (1): 11-8. doi: 10.1530/eje-07-0248.

**Recommendation 22: For patients intolerant of or resistant to DA therapy, surgical treatment is recommended. (Evidence grade: Very Low, Weak Recommendation)**

**Recommendation 23: For patients with prolactinoma apoplexy leading to rapid visual deterioration, surgical treatment is preferentially recommended. (Evidence grade: Very Low, Weak Recommendation)**

**22: For patients intolerant of or resistant to DA therapy, surgical treatment is recommended. (Very low certainty evidence)**

| Study ID | Study Design | Population | Risk of Bias | Indirectness | Inconsistency | Publication Bias | Certainty of Evidence | Findings |
| --- | --- | --- | --- | --- | --- | --- | --- | --- |
| Primeau V 2012[1] | Retrospective cohort study | Patients with benign prolactinomas undergoing transsphenoidal surgery | Serious | Not serious | Not serious | Not serious | Very low | The study reported that among 63 operated patients, 21% had surgery for dopamine agonist (DA) intolerance and 41% for DA resistance, confirming these as common indications. In a subgroup of 15 patients with preoperative DA resistance, postoperative DA therapy resulted in a significant decrease in prolactin levels, with 47% achieving normalization on lower DA doses compared to preoperative requirements. The authors noted a postoperative recurrence rate of 34% but suggested that surgery remains an important alternative to avoid the long-term risks associated with high-dose DA therapy.  The certainty of the evidence was rated down by one level from an initial rating of **Low** (due to the observational study design) to **Very Low**, due to the following:  The retrospective, single-arm cohort design carries a serious **risk of bias**, particularly in patient selection, the lack of a control group for comparison, and potential biases in outcome measurement and reporting.  **Very low certainty evidence** indicates that in patients’ intolerant of or resistant to DA therapy, surgery is a reported clinical intervention. We **are very uncertain** about the effect of surgery on restoring DA responsiveness or on long-term outcomes for these specific indications. |

**Recommendation 23: For patients with prolactinoma apoplexy leading to rapid visual deterioration, surgical treatment is preferentially recommended. (Very low certainty evidence)**

| Study ID | Study Design | Population | Risk of Bias | Indirectness | Inconsistency | Publication Bias | Certainty of Evidence | Findings |
| --- | --- | --- | --- | --- | --- | --- | --- | --- |
| Primeau V 2012[1] | Retrospective cohort study | Patients with benign prolactinomas undergoing transsphenoidal surgery | Serious | Not serious | Not serious | Not serious | Very low | Among 63 operated patients, 16% (10 cases) underwent surgery for acute complications, most commonly tumor apoplexy or acute visual deterioration. This subgroup had larger tumors and more severe symptoms. The authors stated that surgery is the intervention for acute neurological deficit such as visual loss, aiming to rapidly relieve compression to prevent irreversible damage, with a postoperative remission rate of 10% in this subgroup.  The certainty of the evidence is rated as **Very Low**. The initial rating for observational studies is Low. It was downgraded by one level due to **serious risk of bias** inherent in the retrospective cohort design (e.g., selection bias, lack of a control group).  **Very low certainty evidence** indicates that in patients with prolactinoma apoplexy leading to rapid visual deterioration, surgery is performed as a clinical intervention. We are very uncertain about the comparative effectiveness of surgery versus conservative management for this specific emergency indication, or about its precise effect on the prevention of irreversible neurological damage. |
| Lam et al., 2012[2] | Systematic review | Patients with pituitary adenoma presenting non-surgical cerebrospinal-fluid rhinorrhoea | Serious | Not serious | Not serious | Not serious | Very low | The review of 52 CSF-leak-related cases reported that surgical intervention achieved resolution in 88% of cases.  The certainty of the evidence is rated as **Very Low**. The initial rating for systematic reviews of observational studies is Low. It was downgraded by one level due to **serious risk of bias** in the included studies (e.g., retrospective designs, potential selection bias).  **Very low certainty evidence** indicates that surgery is an effective intervention for resolving CSF rhinorrhea in patients with pituitary adenoma. We **are very uncertain** about the effect of surgical treatment for patients with prolactinoma apoplexy leading to rapid visual deterioration, based on this indirect evidence. |

References:

1. Primeau V, Raftopoulos C, Maiter D. Outcomes of transsphenoidal surgery in prolactinomas: improvement of hormonal control in dopamine agonist-resistant patients[J]. Eur J Endocrinol, 2012, 166 (5): 779-86. doi: 10.1530/eje-11-1000.
2. Lam G, Mehta V, Zada G. Spontaneous and medically induced cerebrospinal fluid leakage in the setting of pituitary adenomas: review of the literature[J]. Neurosurg Focus, 2012, 32 (6): E2. doi: 10.3171/2012.4. Focus 1268.

**Recommendation 24: If CSF rhinorrhea is suspected during DA treatment, nasal endoscopy can be performed, or, if available, testing of nasal discharge for β2-transferrin or β-trace protein is suggested (Evidence grade: Very Low, Weak Recommendation). When tumor-induced or pharmacologically induced CSF rhinorrhea occurs, immediate surgical repair is indicated. (Good Practice Statement)**

**24a: If CSF rhinorrhea is suspected during DA treatment, nasal endoscopy can be performed, or, if available, testing of nasal discharge for β2-transferrin or β-trace protein is suggested**

| Study ID | Study Design | Population | Risk of Bias | Indirectness | Inconsistency | Publication Bias | Certainty of Evidence | Findings |
| --- | --- | --- | --- | --- | --- | --- | --- | --- |
| Suliman et al. 2007[1] | Retrospective cohort study | Patients with invasive giant prolactinomas and non-functioning pituitary adenomas | Serious | Serious | Not serious | Not serious | Very low | The study reported the use of laboratory confirmation for cerebrospinal fluid (CSF) rhinorrhea, specifically using β-transferrin testing.  The certainty of the evidence is rated as **Very Low**. The initial rating for observational studies is Low. It was downgraded by two levels due to the following:  **Risk of Bias**: The retrospective study design carries a high risk of bias in patient selection and outcome assessment.  **Indirectness**: The evidence provides only indirect support for the recommendation due to **population and outcome** differences: The study focused on patients with invasive giant prolactinomas, which is a distinct and more severe subgroup compared to the broader population of prolactinoma patients receiving DA treatment who are at risk for CSF rhinorrhea. And it reported on the use of β-transferrin testing, but did not provide comparative data on its diagnostic accuracy (e.g., sensitivity, specificity) versus clinical suspicion alone or other tests (nasal endoscopy, β-trace protein) in this specific diagnostic scenario.  **Very low certainty evidence** indicates that β-transferrin testing was used in a study to confirm CSF rhinorrhea in patients with giant prolactinomas. We **are very uncertain** about the diagnostic accuracy of β-transferrin testing, nasal endoscopy, or β-trace protein testing for detecting suspected CSF rhinorrhea during dopamine agonist treatment, or about the effect of using these tests on patient management decisions. |
| Česák et al. 2018[2] | Retrospective case series + systematic literature review | Patients with giant prolactinomas | Serious | Serious | Not serious | Not serious | Very low | The authors identified β-2 transferrin as a specific marker for detecting cerebrospinal fluid (CSF) rhinorrhea and recommended preoperative high-resolution CT or MRI cisternography for localization. They noted nasal endoscopy as a useful adjunct. The review stated that once CSF rhinorrhea is confirmed, endoscopic endonasal surgical repair is the first-line treatment, with non-operative management reserved for specific situations.  The certainty of the evidence is rated as **Very Low**. It was downgraded by two levels due to the following:  **Risk of Bias**: The evidence is derived from a retrospective case series and a review of predominantly observational literature, which carries a high risk of selection and reporting bias.  **Indirectness**: The evidence provides only indirect support for the recommendation due to **population** differences: The study and review focused exclusively on giant prolactinomas, a severe and uncommon subtype. This population differs substantially from the broader population of prolactinoma patients receiving DA treatment in whom CSF rhinorrhea may be suspected.  **Very low certainty evidence** indicates that β-2 transferrin testing, nasal endoscopy, and imaging are reported as diagnostic approaches for CSF rhinorrhea in patients with giant prolactinomas. We **are very uncertain** about the diagnostic accuracy of these modalities for detecting suspected CSF rhinorrhea specifically during dopamine agonist treatment in the broader prolactinoma population, or about the effect of implementing such a diagnostic pathway on clinical outcomes. |

References:

1. Suliman S G, Gurlek A, Byrne J V, et al. Nonsurgical cerebrospinal fluid rhinorrhea in invasive macroprolactinoma: incidence, radiological, and clinicopathological features[J]. J Clin Endocrinol Metab, 2007, 92 (10): 3829-35. doi: 10.1210/jc.2007-0373.
2. Česák T, Poczos P, Adamkov J, et al. Medically induced CSF rhinorrhea following treatment of macroprolactinoma: case series and literature review[J]. Pituitary, 2018, 21 (6): 561-70. doi: 10.1007/s11102-018-0907-1.

**24b: When tumor-induced or pharmacologically induced CSF rhinorrhea occurs, immediate surgical repair is indicated. (Good Practice Statement)**

This portion qualifies as a Good Practice Statement (GPS) because it addresses a critical and time-sensitive clinical scenario where direct comparative evidence from trials is neither feasible nor ethical to obtain. The recommendation reflects a high degree of expert consensus that delaying surgical intervention in cases of tumor- or drug-induced CSF rhinorrhea would lead to unacceptable risks of serious complications, such as meningitis or intracranial infection. Therefore, despite the absence of high-quality direct evidence, the benefit of immediate surgical repair is considered overwhelmingly clear and necessary to prevent patient harm.

**Recommendation 25: For women with macroadenomas who have fertility desires, debulking surgery can be an alternative to DA therapy to reduce the risk of symptomatic tumor enlargement during future pregnancy. (Evidence grade: Low, Weak Recommendation).**

| Study ID | Study Design | Population | Risk of Bias | Indirectness | Inconsistency | Publication Bias | Certainty of Evidence | Findings |
| --- | --- | --- | --- | --- | --- | --- | --- | --- |
| Andereggen et al. 2017[1] | Retrospective cohort study | Premenopausal women with newly diagnosed prolactinoma who have not received prior dopamine-agonist therapy | Not serious | Not serious | Not serious | Not serious | Low | The study reported that among patients with macroadenomas, 40% of those who underwent surgery required chronic DA therapy postoperatively, compared to 91% of those in the initial medical treatment group. The surgical cohort experienced no permanent complications, with manageable peri-operative events. The mean patient age was 34 years, and 77% presented with amenorrhea. The study noted that macroadenoma recurrence rates did not differ significantly between the surgical and medical groups, and postoperative recurrences were managed with DA therapy.  The certainty of the evidence is rated as **Low**.  **Low certainty evidence** suggests that for premenopausal women with prolactin macroadenomas, cytoreductive surgery **may** reduce the need for long-term dopamine agonist therapy compared to initial medical management. This indicates that surgery could be an alternative for patients with fertility desires to potentially lower the risk of symptomatic tumor enlargement during a future pregnancy. |
| Yan Z 2015[2] | Retrospective cohort study | 99 women of reproductive age with pituitary prolactin-secreting adenomas | Not serious | Not serious | Not serious | Not serious | Low | The study reported that 76.5% of patients with microadenomas resumed regular menses after surgery. Among patients who had been infertile before the operation, 82.3% conceived and delivered successfully.  The certainty of the evidence is rated as **Low**.  **Low certainty evidence** suggests that in women of reproductive age with prolactin-secreting microadenomas, surgery **may** be associated with the resumption of regular menses and successful pregnancy outcomes. We **are very uncertain** about the effect of debulking surgery for macroadenomas on fertility and pregnancy-related outcomes, or about its comparative effect against dopamine agonist therapy for reducing the risk of symptomatic tumor enlargement during future pregnancy. |

References:

1. Andereggen L, Frey J, Andres R H, et al. 10-year follow-up study comparing primary medical vs. surgical therapy in women with prolactinomas[J]. Endocrine, 2017, 55 (1): 223-30. doi: 10.1007/s12020-016-1115-2.
2. Yan Z, Wang Y, Shou X, et al. Effect of transsphenoidal surgery and standard care on fertility related indicators of patients with prolactinomas during child-bearing period[J]. Int J Clin Exp Med, 2015, 8 (11): 21557-64.

**3.3 Radiotherapy**

**Recommendation 26: Radiotherapy can be considered for prolactinomas under the following circumstances: (1) Resistance and/or intolerance to DA therapy; (2) Contraindications to general anesthesia for surgery, inability to achieve surgical resection, postoperative residue, or postoperative recurrence; (3) Refractory or metastatic pituitary prolactinomas. (Evidence grade: Very Low, Weak Recommendation).**

| Study ID | Study Design | Population | Risk of Bias | Indirectness | Inconsistency | Publication Bias | Certainty of Evidence | Findings |
| --- | --- | --- | --- | --- | --- | --- | --- | --- |
| Ježková J et al. 2019[1] | Retrospective cohort study | 28 prolactinoma patients treated with Gamma Knife radiosurgery (GKRS) | Serious | Serious | Not serious | Not serious | Very low | Among patients treated with Gamma Knife radiosurgery (GKRS), 60.7% (17 patients) underwent GKRS due to dopamine agonist (DA) resistance, and 17.9% (5 patients) due to DA intolerance. Overall, 82.1% of patients achieved normoprolactinaemia post-GKRS. The study addressed locally invasive disease but did not include patients with distant metastases, noting insufficient evidence for that specific subgroup.  The certainty of the evidence is rated as **Very Low**. The initial rating for observational studies is Low. It was downgraded by two levels due to the following:  **Risk of Bias**: The evidence stems from a retrospective cohort study with a small sample size (n=28), which carries a high risk of selection and reporting bias.  **Indirectness**: The evidence provides only indirect support for the broad recommendation due to **population** difference:  Population: The study focused on a mixed group of patients primarily with DA resistance or intolerance. It provides limited direct evidence for other specific circumstances outlined in the recommendation, such as patients with surgical contraindications, postoperative residue, or true refractory or metastatic disease.  **Very low certainty evidence** indicates that in patients with DA-resistant or intolerant prolactinomas, GKRS is reported as an intervention associated with hormonal normalization in a case series. We **are very uncertain** about the comparative effectiveness or safety of radiotherapy versus other therapeutic strategies for these indications, its efficacy in patients with contraindications to surgery or postoperative residue/recurrence, or its role in treating refractory or metastatic disease. |
| Ježková J et al. 2009[2] | Retrospective cohort study | 35 prolactinoma patients | Serious | Serious | Not serious | Not serious | Very low | The study explicitly included patients with dopamine agonist (DA) resistance (45.7%) and intolerance (31.4%). An overall biochemical success rate of 80% was reported, and the tumor control rate was 97.1%. The study population included patients with prior surgery (28.6%) and a majority with macroadenomas, some with cavernous sinus invasion.  The certainty of the evidence is rated as **Very Low**. It was downgraded by two levels due to the following:  **Risk of Bias**: The evidence is derived from a retrospective cohort study with a small sample size, which carries a high risk of selection, performance, and detection bias.  **Indirectness**: The evidence provides only indirect support for the broad recommendation due to **population** difference: The study did not explicitly enroll patients with “contraindications to general anesthesia” or “inability to achieve surgical resection” as primary indications. Its applicability to these specific subpopulations is indirect, inferred from the inclusion of patients with prior surgery, large tumors, and invasive features. The evidence does not address refractory or metastatic prolactinomas.  **Very low certainty evidence** indicates that in patients with DA-resistant or intolerant prolactinomas, including those with prior surgery or invasive tumors, radiosurgery is reported as an intervention associated with biochemical control and tumor stabilization. We **are very uncertain** about the effect of radiotherapy for patients with general anesthesia contraindications, truly non-resectable tumors, or refractory/metastatic disease, based on this indirect evidence. |

References:

1. Ježková J, Hána V, Kosák M, et al. Role of gamma knife radiosurgery in the treatment of prolactinomas[J]. Pituitary, 2019, 22 (4): 411-21. doi: 10.1007/s11102-019-00971-x.
2. Jezková J, Hána V, Krsek M, et al. Use of the Leksell gamma knife in the treatment of prolactinoma patients[J]. Clin Endocrinol (Oxf), 2009, 70 (5): 732-41. doi: 10.1111/j.1365-2265.2008.03384.x.

**Recommendation 27: Consider discontinuing DA 1-2 months before radiotherapy; if DA needs to be restarted after radiotherapy, it is suggested to begin 1 month after radiotherapy. (Evidence grade: Very Low, Weak Recommendation)**

| Study ID | Study Design | Population | Risk of Bias | Indirectness | Inconsistency | Publication Bias | Certainty of Evidence | Findings |
| --- | --- | --- | --- | --- | --- | --- | --- | --- |
| Pouratian N et al. 2006[1] | Retrospective case series | Patients with refractory prolactinoma who had failed or were intolerant to medical and/or surgical therapy | Serious | Not serious | Not serious | Not serious | Very low | The study reported that among patients using dopamine agonists (DA) at the time of Gamma Knife radiosurgery (GKRS), 7.7% (1/13) achieved endocrine remission, compared to 50% (5/10) of those not using DA at the time of GKRS.  The certainty of the evidence is rated as **Very Low**. It was downgraded by one level due to serious **risk of bias**. The retrospective case series design, with a very small sample size and lack of control for potential confounders (e.g., differences in tumor characteristics or prior treatment intensity between the groups), carries a high risk of producing a biased estimate of the association between DA use and radiotherapy outcomes.  **Very low certainty evidence** indicates that in a small series of patients with refractory prolactinoma, the use of dopamine agonists during radiosurgery was associated with a lower rate of endocrine remission. We **are very uncertain** about the optimal timing for discontinuing or restarting DA in relation to radiotherapy, or about the causal effect of DA on radiotherapy efficacy. |
| Cohen-Inbar et al. 2015[2] | Retrospective single-center study | Patients with refractory prolactinoma resistant to both medical and surgical treatment | Serious | Not serious | Not serious | Not serious | Very low | The overall endocrine remission rate was 50%. There was a trend toward lower remission rates in patients concurrently using DA during GKRS (38.1% vs. 50%, p = 0.498). The median time to endocrine remission was 20 months in the concurrent DA group versus 14 months in the non-concurrent group.  The certainty of the evidence is rated as **Very Low**. The initial rating for observational studies is Low. It was downgraded by one level due to **serious risk of bias**. The retrospective study design, with its inherent limitations in controlling for confounding factors and the non-randomized comparison of groups, carries a high risk of producing a biased estimate of the association between DA timing and radiotherapy outcomes.  **Very low certainty evidence** indicates that in a cohort of patients with refractory prolactinoma, the concurrent use of dopamine agonists during radiosurgery was associated with a non-significant trend toward lower remission rates and a longer time to remission. We **are very uncertain** about the optimal timing for discontinuing or restarting DA in relation to radiotherapy based on this evidence. |
| Hung et al. 2019[3] | Retrospective cohort study | Patients with refractory prolactinoma unresponsive to both medical and surgical interventions | Serious | Not serious | Serious | Not serious | Very low | Univariate analysis showed an association between dopamine agonist (DA) use at the time of stereotactic radiosurgery (SRS) and reduced endocrine remission rates (54% vs. 73%). However, multivariate analysis did not confirm this association as statistically significant.  The certainty of the evidence is rated as **Very Low**. The initial rating for observational studies is Low. It was downgraded by one level due to **serious risk of bias**. The retrospective study design is susceptible to residual confounding and bias, despite attempts at multivariate adjustment.  **Very low certainty evidence** indicates that in patients with refractory prolactinoma, an initial univariate association between DA use during SRS and lower remission rates was not sustained in a multivariate analysis controlling for other factors. We **are very uncertain** about the effect of discontinuing or restarting DA around the time of radiotherapy on endocrine outcomes |

References:

1. Pouratian N, Sheehan J, Jagannathan J, et al. Gamma knife radiosurgery for medically and surgically refractory prolactinomas[J]. Neurosurgery, 2006, 59 (2): 255-66; discussion -66. doi: 10.1227/01. Neu.0000223445.22938. Bd.
2. Cohen-Inbar O, Xu Z, Schlesinger D, et al. Gamma Knife radiosurgery for medically and surgically refractory prolactinomas: long-term results[J]. Pituitary, 2015, 18 (6): 820-30. doi: 10.1007/s11102-015-0658-1.
3. Hung Y C, Lee C C, Yang H C, et al. The benefit and risk of stereotactic radiosurgery for prolactinomas: an international multicenter cohort study[J]. J Neurosurg, 2020, 133 (3): 717-26. doi: 10.3171/2019.4. Jns183443.

# **Part 4. Male and Refractory Prolactinoma**

**Recommendation 28: For male patients with prolactinomas who develop bromocriptine resistance, switching to cabergoline, combined with surgical debulking and/or radiotherapy, is recommended, along with close follow-up. (Evidence grade: Very Low, Weak Recommendation)**

| Study ID | Study Design | Population | Risk of Bias | Indirectness | Inconsistency | Publication Bias | Certainty of Evidence | Findings |
| --- | --- | --- | --- | --- | --- | --- | --- | --- |
| Delgrange E 1997[1] | Retrospective cohort study | 96 patients with prolactinoma (45 males, 51 females) | Not serious | Serious | Not serious | Not serious | Very low | The study reported a significantly higher rate of bromocriptine resistance in male patients (30%) compared to female patients (5%). Among resistant male patients, tumors were more frequently invasive macroadenomas and exhibited a higher Ki-67 index. One resistant male patient developed a malignant prolactinoma with spinal metastasis.  The certainty of the evidence is rated as **Very Low**. It was downgraded by one level due to **serious indirectness**. The study provides indirect support for the recommendation due to a **population and intervention** mismatch: While the study describes the clinical characteristics (high resistance rate, invasiveness) of male prolactinomas, it does not evaluate the outcomes of patients who were specifically managed with the recommended strategy of switching to cabergoline combined with surgery/radiotherapy. And the study does not evaluate the effectiveness of the specific interventions recommended (switching to cabergoline, combined therapy). It primarily describes the natural history and pathology of bromocriptine-resistant tumors in males.  **Very low certainty evidence** indicates that in male patients with prolactinoma, bromocriptine resistance is associated with more invasive tumors and higher proliferative activity. We **are very uncertain** about the effect of switching to cabergoline, combined with surgical debulking and/or radiotherapy, on clinical outcomes for this specific subgroup based on this indirect evidence. |

References:

1. Delgrange E, Trouillas J, Maiter D, et al. Sex-related difference in the growth of prolactinomas: a clinical and proliferation marker study[J]. J Clin Endocrinol Metab, 1997, 82 (7): 2102-7. doi: 10.1210/jcem.82.7.4088.

**Recommendation 29: For patients with refractory or metastatic pituitary prolactinomas, a comprehensive treatment plan formulated by a multidisciplinary team is recommended. (Good Practice Statement).**

**Rationale as a Good Practice Statement:**

1. Clarity and Feasibility:

•Defined target population: Patients meeting criteria for “refractory” disease (invasive imaging features, resistance to standard therapies, rapid growth, high proliferative markers) or with documented distant metastases.

•Defined workflow: A multidisciplinary team (MDT) conducts a comprehensive assessment and decides on management strategies, such as cabergoline dose escalation, repeat surgery, radiotherapy, or initiation of temozolomide chemotherapy.

•Defined monitoring: New neurological deficits, back pain, or discordance between prolactin level and tumour size trigger evaluation for metastatic foci (brain, spine, liver, lymph nodes, bone).

2. Necessity:

•Disease characteristics: Pituitary prolactinomas are highly invasive, prone to metastasis, and have a high failure rate with monotherapy, exceeding the capacity of any single specialty.

•Avoidance of delay: Without MDT coordination, off-label use of temozolomide may be delayed due to procedural barriers or lack of experience, allowing disease progression.

3. Substantial and Certain Net Benefit:

•Survival benefit: In a retrospective cohort of 94 patients, 62% achieved remission or stable disease with temozolomide; concurrent radiotherapy further increased the response rate.

•Manageable risk: MGMT <50% and functioning tumours predict better response; MSH6 deficiency predicts resistance—both readily incorporated into MDT decision-making.

•Cost-effectiveness: A single MDT meeting can finalise the treatment plan, eliminating multiple referrals and redundant investigations.

4. Prohibitive Opportunity Cost of Collecting Direct Evidence

•RCT not feasible: Aggressive/metastatic cases are rare and heterogeneous; randomisation poses ethical obstacles; existing very-low-quality evidence already indicates MDT-based combined therapy as the best practical strategy.

•Systematic review prohibitively costly: Need to require integration of surgical, radiotherapeutic, chemotherapeutic, and molecular data beyond guideline time-frame and resources.

5. Explicit Evidence Chain

•Refractory prolactinomas are highly invasive and progress rapidly; single-modality treatment is insufficient. Retrospective data show that MDT-coordinated regimens (repeat surgery±radiotherapy±temozolomide) yield remission or stable disease in 62% of patients; MGMT and MSH6 status predict response and resistance. An MDT can complete treatment pathway design and ethical approval in one session, preventing therapeutic delay.

**Recommendation 30:** **The alkylating chemotherapeutic agent temozolomide (TMZ) is recommended as the first-line chemotherapy regimen for refractory or metastatic pituitary prolactinomas. Treatment response should be assessed at 3 months, and the treatment course should be at least 6 months. (Evidence grade: Very Low, Weak Recommendation).**

| Study ID | Study Design | Population | Risk of Bias | Indirectness | Inconsistency | Publication Bias | Certainty of Evidence | Findings |
| --- | --- | --- | --- | --- | --- | --- | --- | --- |
| McCormack A 2018[1] | Cross-sectional survey study | Patients with aggressive pituitary tumors (APT) and pituitary carcinoma (PC) | Serious | Serious | Not serious | Not serious | Very low | Among 166 patients, 157 received temozolomide (TMZ) as first-line chemotherapy. The maximal radiological response was achieved within 6 months for 59% of patients, with efficacy assessable at 3 months. The median treatment duration was 9 months, and patients treated for≥6 months had better outcomes. In a subgroup of 38 prolactinoma patients, TMZ demonstrated an efficacy rate of 45%.  The certainty of the evidence is rated as **Very Low**. The initial rating for this type of evidence is Low. It was downgraded by two levels due to the following:  **Risk of Bias**: The cross-sectional survey design is highly susceptible to selection and reporting bias, lacks a control group, and cannot establish causality or precise effect estimates.  **Indirectness**: The evidence provides indirect support for the recommendation due to **population** differences. The study population comprised a broad mix of aggressive pituitary tumors and carcinomas of various subtypes. While it includes a prolactinoma subgroup, the findings are not specific to the population defined in the recommendation, which may have a different prognosis and response profile compared to other aggressive tumor types or carcinomas.  **Very low certainty evidence** indicates that temozolomide (TMZ) is used as first-line chemotherapy in the management of aggressive pituitary tumors and carcinomas, including prolactinomas, with radiological response assessable at 3 months and a treatment course typically extending to at least 6 months. We **are very uncertain** about the effect of TMZ on specific outcomes for patients with refractory or metastatic prolactinomas, compared to other management strategies. |

Reference:

1. McCormack A, Dekkers O M, Petersenn S, et al. Treatment of aggressive pituitary tumours and carcinomas: results of a European Society of Endocrinology (ESE) survey 2016[J]. Eur J Endocrinol, 2018, 178 (3): 265-76. doi: 10.1530/eje-17-0933.

# **Part 5. Pregnancy**

**Recommendation 31:** **Women with microprolactinomas taking DAs do not need to discontinue medication when attempting conception (Evidence grade: Very Low, Weak Recommendation). For women with macroadenomas, it is suggested to reduce the tumor size to that of a microadenoma before attempting conception (Evidence grade: Very Low, Weak Recommendation). For patients with resistant or persistently growing macroadenomas, surgical treatment followed by conception attempt is recommended. (Evidence grade: Very Low, Weak Recommendation)**

**31a:** **Women with microprolactinomas taking DAs do not need to discontinue medication when attempting conception (Very low certainty evidence).**

| Study ID | Study Design | Population | Risk of Bias | Indirectness | Inconsistency | Publication Bias | Certainty of Evidence | Findings |
| --- | --- | --- | --- | --- | --- | --- | --- | --- |
| Stalldecker 2010[1] | Retrospective cohort study | 90 women who became pregnant while on cabergoline (47 microadenomas, 34 macroadenomas) | Serious | Serious | Not serious | Not serious | Very low | All subjects were exposed to cabergoline at conception, with most continuing the drug through the first trimester. The study reported a miscarriage rate of 7.2%, a preterm delivery rate of 8.8%, and a major congenital anomaly rate of 3.6%. The authors noted no significant increase in these adverse outcomes compared to the general population, but did not stratify the analysis by adenoma size.  The certainty of the evidence is rated as **Very Low**. It was downgraded by two levels due to the following:  **Risk of Bias**: The retrospective study design is prone to selection and recall bias, and the lack of a direct, comparable control group limits the strength of the safety conclusions.  **Indirectness**: The evidence provides indirect support for the recommendation mainly due to **population** difference: The study included a mixed population of women with both microadenomas and macroadenomas and did not present separate safety analyses for the microadenoma subgroup. Therefore, the findings are not directly applicable to the specific population of interest (women with microprolactinomas).  **Very low certainty evidence** indicates that in a cohort of women who conceived while taking cabergoline for prolactinoma, the observed rates of adverse pregnancy and childhood outcomes were not significantly elevated. We **are very uncertain** about the safety of continuing dopamine agonists specifically for women with microprolactinomas when attempting conception, based on this indirect and limited evidence. |
| Holmgren 1986[2] | Retrospective cohort study | 35 women treated with bromocriptine (9 microadenomas, 26 macroadenomas) | Serious | Serious | Not serious | Not serious | Very low | Tumor-related complications during pregnancy occurred in 20% of women, all of whom had received less than 12 months of bromocriptine therapy prior to conception. Lactation was not associated with adverse effects on tumor growth. The study concluded that bromocriptine should be administered for at least one year before conception, with monthly monitoring of serum prolactin and visual fields during pregnancy.  The certainty of the evidence is rated as **Very Low**. It was downgraded by two levels due to the following:  **Risk of Bias**: The retrospective study design is subject to significant recall and selection bias, and the small sample size limits the reliability of the findings.  **Indirectness**: The evidence provides indirect support for the recommendation mainly due to **population and intervention** differences: The study included a mixed cohort of women with microadenomas and macroadenomas, and the results were not stratified by tumor size. Therefore, the findings are not specific to the population of interest (women with microprolactinomas). And the study evaluated bromocriptine, which is a first-generation dopamine agonist. This provides indirect evidence for the more commonly used contemporary agents (e.g., cabergoline) mentioned in the recommendation.  **Very low certainty evidence** indicates that in a cohort of women with prolactinomas who conceived while taking bromocriptine, tumor-related complications were observed, particularly in those with shorter pre-pregnancy treatment duration. We **are very uncertain** about the safety of continuing dopamine agonist therapy without interruption for women with microprolactinomas when attempting conception, based on this outdated and indirect evidence. |
| Sant’ Anna BG 2020[3] | Retrospective cohort study | 194 women (43.6 % microadenomas, 56.4 % macroadenomas) | Serious | Serious | Serious | Not serious | Very low | Cabergoline was discontinued upon pregnancy confirmation in 89% of cases. The study reported a higher miscarriage rate in patients who continued cabergoline after pregnancy diagnosis (38%) compared to those who stopped (7.5%). Among live births, rates of preterm delivery, low birth weight, congenital malformations, and neuropsychological developmental disorders were reported. The authors concluded that cabergoline use to achieve pregnancy appears safe, but continuation after confirmation may be associated with increased miscarriage risk.  The certainty of the evidence is rated as **Very Low.** It was downgraded by three levels due to the following:  **Risk of Bias**: The retrospective observational design carries a high risk of confounding by indication and other biases; the decision to continue medication was not randomized and associated with other risk factors.  **Indirectness**: The evidence provides indirect support for the recommendation due to **population and intervention** differences: The study included a mixed population of microadenomas and macroadenomas without separate analysis for microadenomas. The safety signal for miscarriage was observed in the overall cohort, not specifically in the microadenoma subgroup. The recommendation addresses medication use when attempting conception. This study primarily assesses the risk of continuing medication after pregnancy is confirmed, which is a different clinical scenario with potentially different risk-benefit considerations.  **Inconsistency**: The study's finding of a higher miscarriage rate with continued cabergoline use is inconsistent with other observational studies that have not shown this association, contributing to uncertainty.  **Very low certainty evidence** indicates that in a cohort of women with prolactinomas, cabergoline continuation after pregnancy diagnosis was associated with a higher observed miscarriage rate compared to discontinuation. We **are very uncertain** about the safety of continuing dopamine agonists when attempting conception specifically in women with microprolactinomas, or about the causal nature of the observed association, based on this indirect and inconsistent evidence. |
| Lebbe 2010[4] | Retrospective controlled study | 72 women (45 microadenomas, 15 macroadenomas, 12 hyperprolactinaemia without adenoma) | Serious | Not serious | Not serious | Not serious | Very low | The study reported spontaneous abortion, medically indicated termination for fetal malformations, and neonatal malformation rates that did not differ significantly from controls. Among a subgroup of 37 women who underwent MRI surveillance during pregnancy, tumor enlargement occurred in 46% (17 women), of whom 14% (5 women) required reintroduction of cabergoline. Postnatal childhood development was reported as normal.  The certainty of the evidence is rated as **Very Low**. The initial rating for observational studies is Low. It was downgraded by one level due to serious **risk of bias:** The retrospective study design is susceptible to selection bias and residual confounding. The evidence is further limited by the small sample size and the fact that it primarily evaluates outcomes after medication discontinuation upon pregnancy, rather than the safety of continuing medication when attempting conception.  **Very low certainty evidence** indicates that in a cohort of women, primarily with microprolactinomas, who discontinued cabergoline upon pregnancy, the observed rates of adverse pregnancy and neonatal outcomes were not significantly elevated compared to controls. We **are very uncertain** about the safety of continuing dopamine agonists when attempting conception, or about the comparative risks of continuation versus discontinuation, based on this limited evidence. |

**31b: For women with macroadenomas, it is suggested to reduce the tumor size to that of a microadenoma before attempting conception (Very low certainty evidence)**

| Study ID | Study Design | Population | Risk of Bias | Indirectness | Inconsistency | Publication Bias | Certainty of Evidence | Findings |
| --- | --- | --- | --- | --- | --- | --- | --- | --- |
| Ono 2010[5] | Prospective cohort study | 85 women who conceived while receiving cabergoline (56 microadenomas, 29 macroadenomas) | Not serious | Serious | Not serious | Not serious | Very low | The study protocol required women with macroadenomas to achieve tumor shrinkage before attempting pregnancy. Treatment was continued until prolactin normalized and/or tumor volume was reduced and ovulation resumed, and was then stopped at gestational week 4. Among 93 pregnancies, 83 live births were reported with no observed tumor enlargement or maternal-neonatal complications in this cohort.  The certainty of the evidence is rated as **Very Low**. It was downgraded by one level due to serious **indirectness**. The evidence provides indirect support for the recommendation due to **intervention** differences: The study evaluated a management protocol that combined pre-conception cabergoline therapy for tumor shrinkage with discontinuation of medication at gestational week 4. This bundled intervention does not isolate the effect of the pre-conception tumor size reduction alone, which is the core of the recommendation. The contribution of early drug discontinuation to the observed outcomes cannot be separated.  **Very low certainty evidence** indicates that in a cohort of women with macroadenomas who underwent pre-conception tumor shrinkage with cabergoline and then discontinued the drug early in pregnancy, no pregnancy-related complications or tumor enlargement were observed. We **are very uncertain** about the effect of reducing tumor size to that of a microadenoma before conception on pregnancy outcomes, compared to other management strategies, based on this indirect evidence. |

**31c: For patients with resistant or persistently growing macroadenomas, surgical treatment followed by conception attempt is recommended (Very low certainty evidence).**

| Study ID | Study Design | Population | Risk of Bias | Indirectness | Inconsistency | Publication Bias | Certainty of Evidence | Findings |
| --- | --- | --- | --- | --- | --- | --- | --- | --- |
| Ono 2010[5] | Prospective cohort study | 85 women who conceived while receiving cabergoline (56 microadenomas, 29 macroadenomas) | Not serious | Serious | Not serious | Not serious | Very low | The study stated that for patients with macroadenomas resistant to or progressing despite dopamine agonists, conventional practice recommends attempting pregnancy only after surgical debulking. The authors noted that this recommendation is based on very-low-quality evidence derived from uncontrolled observational data.  The certainty of the evidence is rated as **Very Low**. It was downgraded by one level due to serious **indirectness.** The study does not provide direct evidence on the outcomes of patients who underwent the recommended surgical strategy. Instead, it cites this strategy as an existing practice point, which itself is based on indirect, uncontrolled observational data. Therefore, this serves as indirect evidence of clinical practice rather than direct evidence of efficacy.  **Very low certainty evidence** indicates that in clinical practice, surgical debulking prior to conception has been considered for patients with dopamine agonist-resistant or growing macroadenomas. We **are very uncertain** about the effect of this surgical strategy on fertility or pregnancy outcomes compared to other approaches, based on this indirect and low-quality evidence. |

References:

1. Stalldecker G, Mallea-Gil MS, Guitelman M, et al. Effects of cabergoline on pregnancy and embryo-fetal development: retrospective study on 103 pregnancies and a review of the literature. *Pituitary*. 2010;13(4):345-350. doi:10.1007/s11102-010-0243-6
2. Holmgren U, Bergstrand G, Hagenfeldt K & Werner S. Women with prolactinoma– effect of pregnancy and lactation on serum prolactin and on tumour growth. Acta Endocrinologica 1986 111 452–459.
3. Sant’ Anna BG, Musolino NRC, Gadelha MR, et al. A Brazilian multicentre study evaluating pregnancies induced by cabergoline in patients harboring prolactino mas. Pituitary 2020;23(2):120–8. 29.
4. Lebbe M, Hubinont C, Bernard P, et al. Outcome of 100 pregnancies initiated un der treatment with cabergoline in hyperprolactinaemic women. Clin Endocrinol (Oxf) 2010;73(2):236–42.
5. Ono M, Miki N, Amano K, et al. Individualized high-dose cabergoline therapy for hyperprolactinemic infertility in women with micro- and macroprolactinomas. *J Clin Endocrinol Metab*. 2010;95(6):2672-2679. doi:10.1210/jc.2009-2605

**Recommendation 32: For pregnant women with microprolactinomas, discontinuation of DA therapy is recommended upon confirmation of pregnancy. (Evidence grade: Very Low, Weak Recommendation)**

| Study ID | Study Design | Population | Risk of Bias | Indirectness | Inconsistency | Publication Bias | Certainty of Evidence | Findings |
| --- | --- | --- | --- | --- | --- | --- | --- | --- |
| Sant’ Anna BG 2020[1] | Retrospective cohort study | 194 women (43.6 % microadenomas, 56.4 % macroadenomas) | Serious | Serious | Not serious | Not serious | Very low | Cabergoline was discontinued upon pregnancy confirmation in 89% of cases. The study reported a higher miscarriage rate in patients who continued cabergoline after pregnancy diagnosis (38%) compared to those who stopped (7.5%). The authors noted that no subgroup analysis was performed specifically for women with microadenomas.  The certainty of the evidence is rated as **Very Low**. The initial rating for observational studies is Low. It was downgraded by two levels due to the following:  **Risk of Bias**: The retrospective observational design carries a high risk of confounding (e.g., the decision to continue medication may be linked to other risk factors for miscarriage).  **Indirectness**: The evidence provides indirect support for the recommendation due to **population** differences. The study did not analyze outcomes separately for women with microadenomas; the observed association is based on the overall cohort, which included a majority of macroadenomas (56.4%). Therefore, the findings are not directly applicable to the specific population of the recommendation.  **Very low certainty evidence** indicates that in a mixed cohort of pregnant women with prolactinomas, continued cabergoline use after pregnancy confirmation was associated with a higher observed miscarriage rate compared to discontinuation. We **are very uncertain** about the effect of discontinuing dopamine agonist therapy specifically for pregnant women with microprolactinomas, or about the causal nature of the observed association. |

Reference:

1. Sant’ Anna BG, Musolino NRC, Gadelha MR, et al. A Brazilian multicentre study evaluating pregnancies induced by cabergoline in patients harboring prolactino mas. Pituitary 2020;23(2):120–8. 29.

**Recommendation 33: For pregnant women with macroadenomas that were not effectively controlled before pregnancy, continuation of DA therapy throughout the pregnancy can be considered. (Evidence grade: Very Low, Weak Recommendation)**

| Study ID | Study Design | Population | Risk of Bias | Indirectness | Inconsistency | Publication Bias | Certainty of Evidence | Findings |
| --- | --- | --- | --- | --- | --- | --- | --- | --- |
| Stalldecker G 2010[1] | Retrospective cohort study | 90 women with prolactinoma (47 microadenomas, 34 macroadenomas) who conceived while receiving cabergoline | Serious | Serious | Not serious | Not serious | Very low | Among 103 pregnancies, approximately 97% of women continued cabergoline during the first trimester. The study reported no increase in major maternal or fetal complications compared to the general population, with isolated cases of mild neurodevelopmental abnormalities and congenital malformations. The findings were not stratified by adenoma size.  The certainty of the evidence is rated as **Very Low**. The initial rating for observational studies is Low. It was downgraded by two levels due to the following:  **Risk of Bias**: The retrospective study design is susceptible to selection and information bias, and the lack of a direct, comparable control group limits the strength of the safety conclusions.  **Indirectness**: The evidence provides indirect support for the recommendation due to **population** differences. The study cohort included both microadenomas and macroadenomas, and outcomes were not reported separately for the subgroup of interest: women with macroadenomas that were not effectively controlled before pregnancy. The safety of continuation may differ in this higher-risk subgroup compared to the overall mixed population.  **Very low certainty evidence** indicates that in a cohort of pregnant women with prolactinomas who continued cabergoline into the first trimester, major adverse outcomes were not increased. We **are very uncertain** about the safety of continuing dopamine agonist therapy throughout pregnancy specifically for women with poorly controlled macroadenomas, based on this indirect evidence. |

Reference:

1. Stalldecker G, Mallea-Gil MS, Guitelman M, et al. Effects of cabergoline on pregnancy and embryo-fetal development: retrospective study on 103 pregnancies and a review of the literature. *Pituitary*. 2010;13(4):345-350. doi:10.1007/s11102-010-0243-6

**Recommendation 34: Routine monitoring of serum PRL levels is not recommended for pregnant women with microadenomas during pregnancy (Evidence grade: Very Low, Weak Recommendation). For pregnant women with macroadenomas, if serum PRL levels rise suddenly during pregnancy (increase >50% from baseline), it may suggest tumor growth, but this must be interpreted in conjunction with clinical symptoms (Evidence grade: Very Low, Weak Recommendation).**

| Study ID | Study Design | Population | Risk of Bias | Indirectness | Inconsistency | Publication Bias | Certainty of Evidence | Findings |
| --- | --- | --- | --- | --- | --- | --- | --- | --- |
| Auriemma R S 2013[1] | Observational survey | 91 hyperprolactinaemic women who conceived after cabergoline-induced ovulation (76 microadenomas, 10 macroadenomas, 5 non-tumoural hyperprolactinaemia) | Serious | Serious | Not serious | Not serious | Very low | Key findings: No hormonal evaluations were performed during pregnancy or lactation. Macroadenoma patients underwent visual-field testing every 3 months, and MRI was performed only if clinical symptoms appeared. The study reported a high rate of live births (88.1%) and no neonatal malformations. Postpartum, 68% of patients remained in remission without restarting medication, and no radiological tumor enlargement was observed.  The certainty of the evidence is rated as **Very Low**. It was downgraded by two levels due to the following:  **Risk of Bias**: The survey methodology and observational design are prone to selection and recall bias, and the lack of systematic monitoring precludes an assessment of the relationship between PRL changes and tumor behavior.  **Indirectness**: The evidence provides indirect support for the recommendation due to **outcome** difference: The study did not evaluate the diagnostic performance of serial PRL measurements (e.g., sensitivity, specificity for tumor growth). It only reports outcomes under a practice of not monitoring PRL. Therefore, it provides indirect evidence against the utility of monitoring but does not directly validate the specific diagnostic threshold (>50% rise) mentioned in the recommendation  **Very low certainty evidence** indicates that in a cohort where routine serum prolactin monitoring was not performed during pregnancy for microadenomas, and where monitoring for macroadenomas was symptom-driven, pregnancy outcomes were favorable and no tumor expansion was detected. We **are very uncertain** about the diagnostic accuracy of a >50% rise in PRL for predicting tumor growth in pregnant women with macroadenomas, or about the effect of omitting routine PRL monitoring on clinical outcomes. |

Reference:

1. Auriemma R S, Perone Y, Di Sarno A, et al. Results of a single-center observational 10-year survey study on recurrence of hyperprolactinemia after pregnancy and lactation[J]. J Clin Endocrinol Metab, 2013, 98 (1): 372-9. Doi: 10.1210/jc.2012-3039.

**Recommendation 35: If significant tumor growth is suspected during pregnancy, accompanied by new symptoms such as headache, visual loss, or visual field defects, non-contrast MRI is recommended, and DA therapy should be restarted (Evidence grade: Very Low, Weak Recommendation). For cases unresponsive to medication or presenting with acute visual loss, impaired consciousness, or other critical conditions, surgery is a reasonable option. (Evidence grade: Very Low, Weak Recommendation)**

| Study ID | Study Design | Population | Risk of Bias | Indirectness | Inconsistency | Publication Bias | Certainty of Evidence | Findings |
| --- | --- | --- | --- | --- | --- | --- | --- | --- |
| Primeau V 2012[1] | Retrospective cohort study | Patients with benign prolactinoma undergoing transsphenoidal surgery | Serious | Not serious | Not serious | Not serious | Very low | Among 63 patients, 16% (10 cases) underwent emergency surgery for acute complications, most commonly tumor apoplexy or acute visual deterioration. This subgroup had larger tumors and more severe symptoms. The authors stated that surgery is the intervention for acute neurological deficit, with the primary goal of salvaging visual and neurological function.  The certainty of the evidence is rated as **Very Low**. The initial rating for observational studies is Low. It was downgraded by one level due to serious **risk of bias** inherent in the retrospective cohort design.  **Very low certainty evidence** indicates that in a non-pregnant cohort, surgery is performed for acute complications of prolactinoma such as apoplexy or visual loss. We **are very uncertain** about the effectiveness and optimal timing of restarting dopamine agonist therapy versus proceeding directly to surgery for managing symptomatic tumor growth during pregnancy, based on this indirect evidence. |
| Smith TR 2015[2] | Retrospective cohort study | Sixty-six prolactinoma patients treated by endoscopic endonasal transsphenoidal surgery | Serious | Serious | Not serious | Not serious | Very low | The study described one pregnant patient who underwent surgery after declining dopamine agonist therapy. Headache and visual disturbance were common preoperative symptoms and showed improvement postoperatively. Among patients with DA intolerance or resistance, surgery achieved normoprolactinaemia in a proportion of cases without subsequent DA.  The certainty of the evidence is rated as **Very Low**. The initial rating for observational studies is Low. It was downgraded by two levels due to the following:  **Risk of Bias**: The retrospective study design carries a high risk of selection and information bias.  Indirectness: The evidence provides only indirect support for the recommendation due to **population and intervention** differences: The primary evidence is derived from a general, predominantly non-pregnant surgical cohort. The single pregnant case provides minimal direct evidence. The management of symptomatic tumor growth during pregnancy involves distinct physiological and risk considerations not addressed in the general cohort. The study does not evaluate the specific intervention of restarting DA therapy for symptomatic growth in pregnancy, which is the first step recommended. It provides evidence only for the alternative pathway (surgery) after DA is declined or fails.  **Very low certainty evidence** indicates that in a general prolactinoma cohort, surgery is an intervention for symptomatic patients, including rare cases in pregnancy, and can be effective after DA failure. We **are very uncertain** about the effect of restarting DA therapy versus proceeding to surgery for managing symptomatic tumor growth during pregnancy, or about the optimal sequence of interventions, based on this indirect evidence. |

References:

1. Primeau V, Raftopoulos C, Maiter D. Outcomes of transsphenoidal surgery in prolactinomas: improvement of hormonal control in dopamine agonist-resistant patients[J]. Eur J Endocrinol, 2012, 166 (5): 779-86. doi: 10.1530/eje-11-1000.
2. Smith TR, Hulou MM, Huang KT, et al. Current indications for the surgical treatment of prolactinomas. *J Clin Neurosci*. 2015;22(11):1785-1791. doi:10.1016/j.jocn.2015.06.001

**Recommendation 36: Breastfeeding is encouraged postpartum. If DA drug therapy is required due to the medical condition, breastfeeding should be discontinued. (Evidence grade: Very Low, Weak Recommendation)**

| Study ID | Study Design | Population | Risk of Bias | Indirectness | Inconsistency | Publication Bias | Certainty of Evidence | Findings |
| --- | --- | --- | --- | --- | --- | --- | --- | --- |
| Auriemma R S 2013[1] | Retrospective cohort study | Hyperprolactinaemic women who conceived after cabergoline-induced ovulation (76 microadenomas, 10 macroadenomas, 5 non-tumoural hyperprolactinaemia) | Serious | Serious | Not serious | Not serious | Very low | Among 88 of 91 patients who breastfed for 1-6 months, no tumor enlargement or symptomatic worsening was observed. Relapse rates did not differ by breastfeeding duration. In all 29 patients who required re-initiation of cabergoline postpartum, therapy was started only after complete cessation of breastfeeding.  The certainty of the evidence is rated as **Very Low**. It was downgraded by two levels due to the following:  **Risk of Bias:** The retrospective survey design is prone to selection and recall bias.  **Indirectness:** The evidence provides indirect support for the recommendation due to **intervention and outcome** differences: The study reports on a practice where medication was withheld during breastfeeding. It provides no evidence on the safety of the converse scenario—continuing medication during breastfeeding—which is the specific risk the recommendation aims to mitigate. The evidence supports one half of the recommendation (breastfeeding without medication appears safe in this cohort) but is indirect for the other (discontinuing breastfeeding if medication is needed, as this was the universal practice in the study). And the study did not assess infant outcomes or drug exposure through breast milk, which are critical considerations for the part of the recommendation concerning medication use during lactation.  **Very low certainty evidence** indicates that in this cohort, breastfeeding was not associated with observed tumor growth or increased relapse risk, and dopamine agonist therapy was reinstated only after breastfeeding was stopped. We **are very uncertain** about the general safety of continuing dopamine agonist therapy during breastfeeding, or about the comparative risks and benefits of discontinuing breastfeeding to resume treatment versus other management strategies. |

Reference:

1. Auriemma R S, Perone Y, Di Sarno A, et al. Results of a single-center observational 10-year survey study on recurrence of hyperprolactinemia after pregnancy and lactation[J]. J Clin Endocrinol Metab, 2013, 98 (1): 372-9. doi: 10.1210/jc.2012-3039.
